# Explicit Hydration of the Beryllium Trifluoride Anion with One to Three Water Molecules: BeF_3_^–^(H_2_O)_*n*=1–3_

**DOI:** 10.1021/acs.jpca.6c01068

**Published:** 2026-04-21

**Authors:** Kayleigh R. Autry, Gregory S. Tschumper

**Affiliations:** † Department of Chemistry and Biochemistry, 8063 The University of Alabama, Tuscaloosa, Alabama 35487, United States; ‡ Department of Chemistry, Missouri University of Science and Technology, Rolla, Missouri 65409, United States

## Abstract

This work investigates the microhydration of the beryllium trifluoride anion (BeF_3_^–^) with up to three water molecules (BeF_3_^–^(H_2_O)_*n*_ where *n* = 1–3). Full geometry optimizations and harmonic vibrational frequencies were computed on various BeF_3_^–^(H_2_O)_*n*_ stationary points using density functional theory methods (B3LYP-D3BJ, B3LYP, ωB97XD, and M06-2X) as well as the CCSD­(T) and MP2 ab initio methods with a triple-ζ correlation-consistent basis set augmented with diffuse functions on all non-hydrogen atoms (haTZ). One BeF_3_^–^(H_2_O)_1_, five BeF_3_^–^(H_2_O)_2_ and 11 BeF_3_^–^(H_2_O)_3_ minima were identified, most of which have not been reported in the literature to date. Two of the new BeF_3_^–^(H_2_O)_3_ configurations have lower electronic energies than the previously reported trihydrate structure by nearly 1 kcal mol^–1^, but the previous structure has the lowest energy when harmonic zero-point vibrational energies are included. For the mono- and dihydrates, the water molecule(s) prefer to bind directly to the beryllium trifluoride anion via double ionic hydrogen bonds (DIHBs) to form planar structures with C_2*v*_ symmetry. With the introduction of a third water molecule, the hydration pattern associated with the lowest-energy structure (with C_3_ symmetry) changes from solely DIHBs to a hydrogen-bonded network that also includes water–water interactions. The CCSD­(T)/haTZ electronic dissociation energies for the BeF_3_^–^(H_2_O)_*n*_ global minima are 16.0, 30.1, and 43.6 kcal mol^–1^ for *n* = 1, 2, and 3, respectively, and these values decrease by about 5% when a counterpoise procedure is applied. The CCSD­(T)/haTZ harmonic OH stretching frequencies of H_2_O decrease appreciably when donating hydrogen bonds to BeF_3_^–^ and/or H_2_O molecules. The magnitude of these shifts is strongly dependent on the hydration motif. For the symmetric structures exhibiting only DIHB contacts, the largest shifts at this level of theory are −168 cm^–1^ for *n* = 1 (C_2*v*_), −138 cm^–1^ for *n* = 2 (C_2*v*_), and −122 cm^–1^ for *n* = 3 (D_3*h*_). In contrast, the C_3_ trihydrate global minimum also has hydrogen bonding between the water molecules, and the largest shift exceeds 200 cm^–1^. The magnitude can even exceed 300 cm^–1^ in structures with an H atom that does not participate in hydrogen bonding.

## Graphical abstract

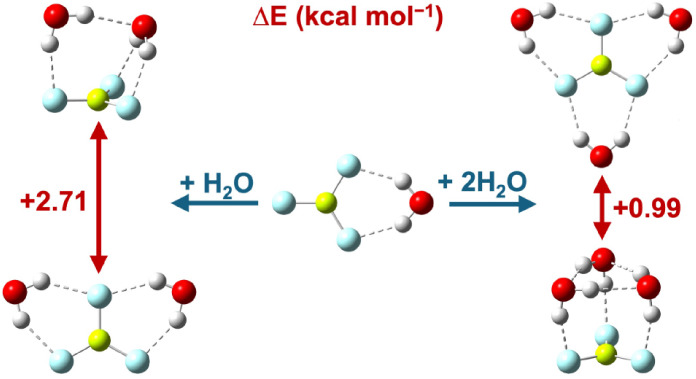

## 1 Introduction

The beryllium trifluoride anion (BeF_3_^–^) is a standard phosphoryl $\left({\mathrm{PO}}_{3}^{-}\right)$ analog used in studying protein phosphorylation due to its trigonal planar geometry and single negative charge.^1–5^ In addition to its utility in the field of biochemistry, the BeF_3_^–^ ion is also of considerable interest in chemistry as a superhalogen. Superhalogens are atomic clusters that mimic halogen atoms while having appreciably stronger vertical detachment energies (VDEs) and electron affinities (EAs).^6,7^ Due to their significant electron binding energies, superhalogens possess strong oxidizing capacity which has led to their growing interest in many areas, such as the development of a new class of salts referred to as “supersalts” ^8,9^ and production of stable ionic liquids (ILs).^10,11^ For example, the hexafluorophosphate anion (PF_6_^–^), the pentafluorosilicate anion (SiF_5_^–^) and the tetrafluoroborate anion (BF_4_^–^) are superhalogens with VDEs greater than 9 eV and their neutrals have EAs exceeding 6 eV.^6,9–13^ Both BF_4_^–^ and PF_6_^–^ are popular anions currently in use to make in ILs. Similarly, the isolated BeF_3_^–^ ion has been reported to have a VDE of 7.406 eV at CCSD­(T)/6-311++G­(d) and an EA of approximately 6.5 eV using density functional theory methods with a triple-ζ basis set.^9,11,14^ It has even been postulated to be a viable anion in creating stable ILs.^11^

Compared to the octahedral PF_6_^–^, trigonal bipyramidal SiF_5_^–^ and tetrahedral BF_4_^–^ ions, the trigonal planar BeF_3_^–^ ion has been studied far less. The D_3*h*_ structure and harmonic vibrational frequencies of BeF_3_^–^ have been reported with different ab initio and DFT methods,^6,7,9,11,14–18^ in addition to some available experimental data. Experimentally, the BeF_3_^–^ ion has been observed when laser-ablated Be atoms, cations, and electrons were reacted with F_2_ diluted in neon gas and a weak infrared absorption band at 1061.4 cm^–1^ was tentatively assigned to BeF_3_^–^.^18^ Their observations were then compared to computational results where the BeF_3_^–^ ion was determined to be stable to halide ion elimination by 87 kcal mol^–1^ with strong stretching absorptions computed at 1049.6 cm^–1^ at the B3LYP/6-311++G­(3df,3pd) level of theory. Additionally, the BeF_3_^–^ ion has been identified experimentally in aqueous solutions using ^19^F nuclear magnetic resonance spectroscopy.^19–24^

The hydration of anions, such as halides and superhalides, is an important area of study as it can help provide valuable information to better understand not only the structure of aqueous solutions and the formation of hydrogen bonds, but can also provide further insight into the effects of these interactions in biological and chemical processes where water might be present.^25–31^ As such, theoretical microhydration studies have shown to be an important tool for examining these interactions on a small scale. The microhydration of anions like PF_6_^–^, SiF_5_^–^ and BF_4_^–^ have been theoretically characterized using sophisticated electronic structure theory methods.^30–33^ The microhydration of BeF_3_^–^ was only recently considered. Structures and binding energies for the minima configurations of BeF_3_^–^ complexed with 1–5 water molecules were previously reported using MP2 and the aug-cc-pVTZ basis set where diffuse functions were truncated for all hydrogen atoms.^34^ A single minimum was reported for the BeF_3_^–^(H_2_O)_1_ monohydrate (1A – C_2*v*_ in Figure 1), while two minima were identified for the BeF_3_^–^(H_2_O)_2_ dihydrate (2A – C_2*v*_ and 2D – C_2*v*_ in Figure 2) along with one additional minimum for each of the larger BeF_3_^–^(H_2_O)_*n*_ hydrates, where *n* = 3, 4, 5. The data from that study indicated that the binding energy per hydrogen bond for each BeF_3_^–^(H_2_O)_*n*_ complex is remarkably consistent (approximately 7.1 ± 0.9 kcal mol^–1^ for *n* = 1 to *n* = 5).

**Figure 1:**
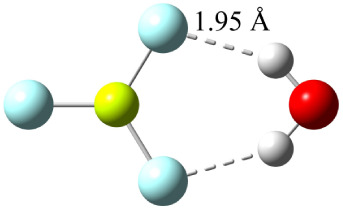
BeF_3_^–^(H_2_O)_1_ global minimum of C_2*v*_ symmetry (denoted as 1A – C_2*v*_) along with its symmetry unique R­(OH···F) distances optimized at CCSD­(T)/haTZ.

**Figure 2:**
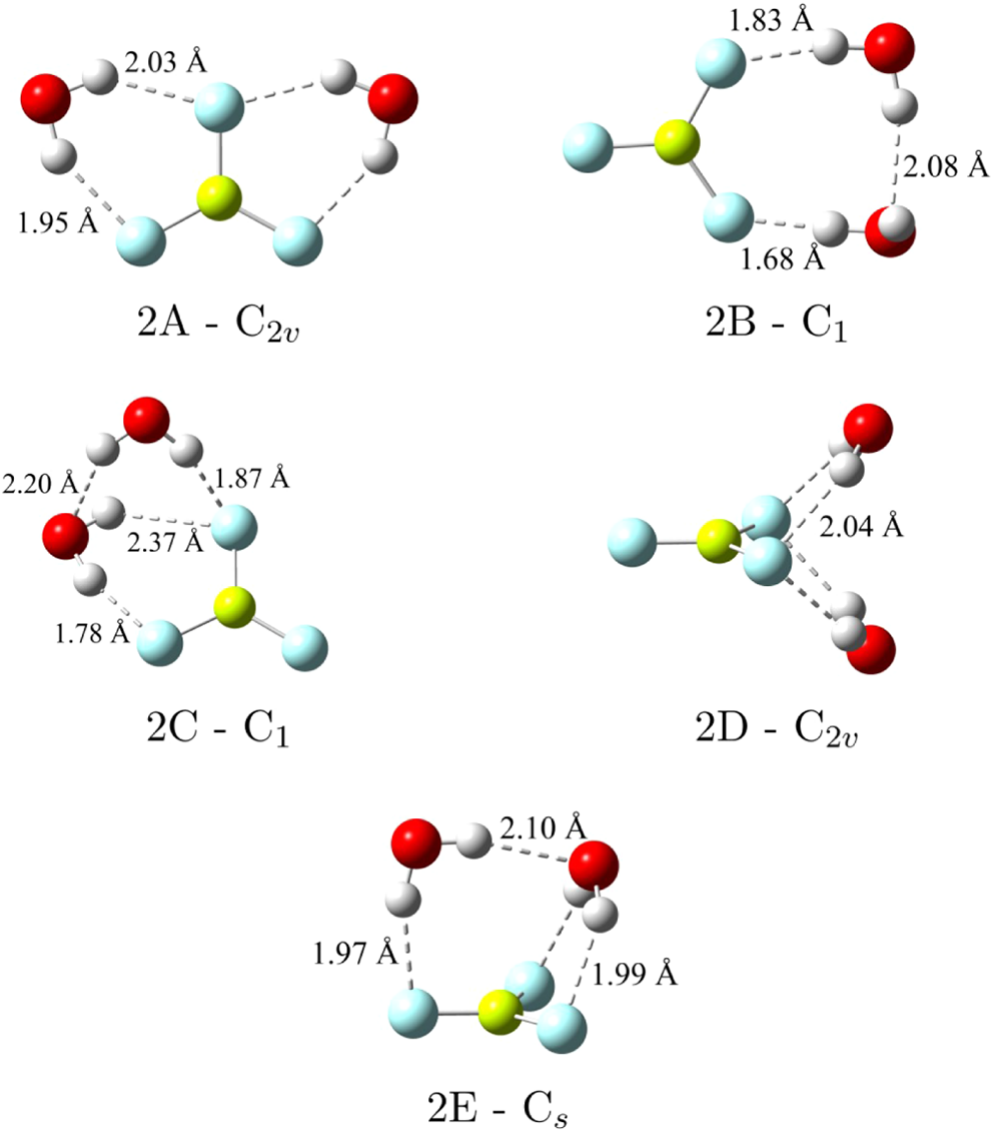
BeF_3_^–^(H_2_O)_2_ low-lying minima and their corresponding point-group symmetries along with their symmetry unique R­(OH···F) and R­(OH···O) distances optimized at CCSD­(T)/haTZ.

The present study is intended to build on this recent work by performing a more thorough search of all possible BeF_3_^–^(H_2_O)_*n*=1–3_ configurations to include not only solvent–solute interactions, but also to examine potential solvent–solvent interactions that were not considered. The consideration of solvent–solvent interactions was found to be essential when characterizing the hydration of PF_6_^–^.^31^ The electronic relative and dissociation energies for the BeF_3_^–^(H_2_O)_*n*_ structures will be analyzed as *n* increases from 1 to 3 water molecules using rigorous ab initio methods as well as benchmarking common density functional theory (DFT) methods all while using correlation-consistent basis sets. This study will also present the shifts in the harmonic symmetric and antisymmetric OH stretching frequencies of a water molecule induced by the explicit solvation of the BeF_3_^–^ ion, along with the zero-point vibrational energy corrections to the relative energies of the di- and trihydrate isomers.

## 2 Computational Details

Full geometry optimizations were performed on various BeF_3_^–^(H_2_O)_*n*=1–3_ configurations using the ab initio MP2 ^35^ and CCSD­(T)^36–38^ methods along with the following density functional theory (DFT) methods: B3LYP,^39^ B3LYP-D3BJ,^39,40^ ωB97XD,^41^ and M06-2X.^42^ A correlation-consistent triple-ζ basis set (cc-pVTZ) was used for all hydrogen atoms while diffuse functions were included for all heavier atoms (aug-cc-pVTZ for Be, F, and O) and is abbreviated as haTZ from here on out.^43,44^ Harmonic vibrational frequencies were computed at each level of theory in order to confirm whether the optimized structures corresponded to a minimum or saddle-point on each respective potential energy surface.

Due to the computational expense of the CCSD­(T)/haTZ frequencies for the three water clusters, our integrated QM:QM and many-body expansion scheme known as the *N*-body:Many-body (Nb:Mb) method,^45–52^ where *N* = 2, was also utilized. This 2b:Mb method has been shown to reliably provide CCSD­(T) quality structures, dissociation energies and vibrational frequencies of similar clusters at a significantly reduced scaling. The 2b:Mb scheme uses a higher level method (CCSD­(T) in this case) to compute the 1- and 2-body interactions within the cluster while then using a low level method (MP2) to compute all of the higher-order contributions. It will be further denoted CCSD­(T):MP2. For each BeF_3_^–^(H_2_O)_2_ configuration, the difference between the conventional CCSD­(T) harmonic frequencies and those computed at CCSD­(T):MP2 was examined and reported in the Supporting Information.

Additional geometry optimizations were carried out on the isolated BeF_3_^–^ and H_2_O fragments to evaluate the electronic dissociation energies (*D*_e_) of the complex based on eq 1 following the supermolecular approach of comparing the sum of the optimized monomer energies to the total energy of the optimized cluster.

1 $$D_{e}=E\left[{\mathrm{BeF}}_{3}^{-}\right]+nE\left[H_{2}O\right]-E[{\mathrm{BeF}}_{3}^{-}\left(H_{2}O{)}_{n}\right]$$

In doing so, an inconsistency^53^ commonly referred to as basis set superposition error (BSSE)^54^ is introduced by the use of finite basis sets. To examine the impact of this inconsistency, the counterpoise (CP) procedure developed by Jansen and Ros^55^ as well as Boys and Bernardi^56^ was applied to the lowest-energy minima for the mono-, di-, and trihydrate following the protocol detailed in prior work.^57^ For 3 or more fragments, there has not been uniquely defined approaches extended beyond the aforementioned method which is outlined in eq 2 below.

2 $$D_{e}^{\mathrm{CP}}=\overset{n+1}{\underset{i=1}{\sum }}[E({\mathrm{fragment}}_{i}{)}_{\mathrm{cluster}\;\mathrm{basis}}^{\mathrm{cluster}\;\mathrm{geom}}-E\left({\mathrm{fragment}}_{i}{)}_{\mathrm{monomer}\;\mathrm{basis}}^{\mathrm{cluster}\;\mathrm{geom}}\right]+D_{e}$$

All optimizations and harmonic frequencies were performed using analytic gradients and Hessians. MP2 and DFT computations were carried out with Gaussian16 ^58^ and all CCSD­(T) computations were done with CFOUR.^59,60^ The frozen-core approximation was employed for all MP2 and CCSD­(T) computations, where the 1*s*-like orbitals of the Be, O and F atoms were excluded from the post-HF procedures. In all cases, pure angular momentum functions (5*d*, 7*f*, etc.) were used in place of their Cartesian counterparts.

## 3 Results and Discussion

### 3.1 Monohydrate Structure and Energetics

The 1A – C_2*v*_ monohydrate structure (shown in Figure 1) was previously identified as a minimum for the BeF_3_^–^(H_2_O)_1_ system at the MP2/haTZ level of theory,^34^ and it is the only minimum identified for this system with the DFT and ab initio procedures employed in the present study. As seen in Figure 1, the hydrogen bond distances between the hydrogens of the water molecule and two fluorine atoms of BeF_3_^–^ (OH···F) are 1.95 Å at CCSD­(T)/haTZ. The distance between the F atoms participating in the hydrogen bonds is ≈2.5 Å, which is consistent with distance-based guidelines for double ionic hydrogen bond (DIHB) motifs typically observed in polyatomic anions where the hydrogen bond acceptors are separated by at least 2.2 Å.^61^ This DIHB motif is structurally analogous to the global minimum monohydrate configurations of BF_4_^–^,^32^ SiF_5_^–^,^33^ and PF_6_^–^,^31^ where the participating F atoms are all approximately 2.3 Å apart. For anions with symmetry equivalent F atoms, the OH···F bond distances consistently increase with the number of fluorine atoms. The CCSD­(T)/haTZ DIHB lengths are 1.95 Å for BeF_3_^–^, 2.03 Å for BF_4_^–^ and 2.10 Å for PF_6_^–^. In SiF_5_^–^, where the axial and equatorial F atoms are not equivalent, two distinct DIHB motifs were identified for the monohydrate. The lowest-energy SiF_5_^–^ monohydrate structure has an asymmetric DIHB motif with one shorter (1.88 Å) and one longer (2.31 Å) OH···F contact, while the C_2*v*_ local minimum forms slightly longer symmetric DIHBs (2.15 Å) with a pair of equatorial F atoms.

The electronic dissociation energy (*D*_e_) computed for the 1A – C_2*v*_ minimum was found to be between 14.69 and 17.05 kcal mol^–1^ depending on the method used, as seen in Table 1, which is approximately 3 times larger than the *D*_e_ for the water dimer^62–64^ computed using similar methods and basis sets. The CCSD­(T)/haTZ dissociation energies of the BF_4_^–^, SiF_5_^–^ and PF_6_^–^ monohydrate (global) minima were reported to be 13.17, 12.21, and 10.67 kcal mol^–1^, respectively.^31–33^ The corresponding value for the BeF_3_^–^(H_2_O)_1_ complex (16.01 kcal mol^–1^) extends the previously noted trend that *D*_e_ decreases as the number of F atoms increases for these monohydrate systems. This observation is consistent with the expectation that the excess charge becomes more delocalized as the number of F atoms in these ions increases, leading to weaker interactions with H_2_O.

**Table 1:** Dissociation Energies (*D*_e_ in kcal mol^–1^) of the BeF_3_^–^(H_2_O)_1_, BeF_3_^–^(H_2_O)_2_, and BeF_3_^–^(H_2_O)_3_ Global Minima Computed with and without the Counterpoise (CP) Procedure Using Various Methods and the haTZ Basis Set

	1A – C_2*v*_		2A – C_2*v*_		3A – C_3_	
Method	*D*_e_	$D_{e}^{\mathrm{CP}}$	*D*_e_	$D_{e}^{\mathrm{CP}}$	*D*_e_	$D_{e}^{\mathrm{CP}}$
CCSD(T)	16.01	15.30	30.13	28.76	43.55	40.96
CCSD(T):MP2	16.01^a^	15.30^a^	30.11	28.74	43.70	41.10
MP2	15.73	15.01	29.53	28.17	42.72	40.20
B3LYP-D3BJ	16.05	15.83	30.16	29.72	44.62	43.97
B3LYP	14.69	14.47	27.44	27.01	38.94	38.30
ωB97XD	15.82	15.61	29.64	29.24	43.98	43.36
M06-2X	17.05	16.84	32.01	31.61	46.04	45.45

a CCSD­(T):MP2 ≡ CCSD­(T) for monomers and dimers.

Compared to the CCSD­(T) dissociation energy of 16.01 kcal mol^–1^, the results obtained with the B3LYP-D3BJ method were essentially identical. The ωB97XD and MP2 *D*_e_ values are also reasonably similar, falling 0.19 and 0.29 kcal mol^–1^ below the CCSD­(T) result. In contrast, dissociation energies computed with the B3LYP and M06-2X methods exhibited larger deviations (exceeding ±1 kcal mol^–1^) from the CCSD­(T)/haTZ value. The CP procedure decreases the CCSD­(T) dissociation energy of 1A – C_2*v*_ by 5%, resulting in a *D*_e_ of 15.30 kcal mol^–1^.

### 3.2 Dihydrate Structures and Energetics

Five low-lying BeF_3_^–^(H_2_O)_2_ stationary points (seen in Figure 2) have been characterized as minima using the CCSD­(T) and CCSD­(T):MP2 methods with the haTZ basis set. The 2A – C_2*v*_ and 2D – C_2*v*_ structures have been previously identified and only exhibit water–anion (OH···F) interactions.^34^ To the best of our knowledge, the other three configurations have been characterized here for the first time (2B – C_1_, 2C – C_1_ and 2E – C_*s*_), and all three structures also have water–water (OH···O) interactions. The BF_4_^–^, SiF_5_^–^ and PF_6_^–^ dihydrate global minima have one H_2_O donating two hydrogen bonds to a pair of F atoms and a second H_2_O forming two additional hydrogen bonds bridging a third F atom to the O atom of the other water molecule over the triangular face of the ion. Although BeF_3_^–^(H_2_O)_2_ has an analogous bridged structure (2E – C_*s*_ in the bottom of Figure 2), the 2A – C_2*v*_ configuration was confirmed to be the global minimum for the BeF_3_^–^(H_2_O)_2_ system at all levels of theory by nearly 3 kcal mol^–1^. The geometry of the 2E – C_*s*_ configuration is presumably less energetically favorable due to the BeF_3_^–^ ion having a planar rather than puckered triangular face (as in BF_4_^–^ SiF_5_^–^ and PF_6_^–^), which leads to a larger FF distance to be bridged by the pair of water molecules (e.g., ≈2.6 Å for BeF_3_^–^ vs ≈2.3 Å for BF_4_^–^ and PF_6_^–^ for the CCSD­(T)/haTZ optimized structures).^31–33^

Compared to the monohydrate, the addition of the second water molecule to form 2A – C_2*v*_ resulted in a lengthening of the OH···F distance by nearly 0.1 Å for the H atoms donating a hydrogen bond to the same F atom. At CCSD­(T)/haTZ, the *D*_e_ for the 2A – C_2*v*_ structure is 30.13 kcal mol^–1^ (Table 1), a near two-fold increase relative to that of the monohydrate. This trend was also observed for the dihydrates of the other anions with *D*_e_ values of 26.43, 24.13, and 22.52 kcal mol^–1^ for BF_4_^–^, SiF_5_^–^ and PF_6_^–^, respectively.^31–33^ As with the monohydrate (1A – C_2*v*_), when applying the CP procedure, the CCSD­(T)/haTZ *D*_e_ of the BeF_3_^–^ dihydrate global minimum (2A – C_2*v*_) decreases by 5% from 30.13 to 28.76 kcal mol^–1^.

As shown in Table 2, all dihydrate structures reported fall within 3.5 kcal mol^–1^ of the 2A – C_2*v*_ global minimum regardless of the method used to compute their relative electronic energies (Δ*E*’s). The new 2B – C_1_ local minimum is the second-lowest energy structure lying 1.61 kcal mol^–1^ higher than the 2A – C_2*v*_ global minimum at CCSD­(T)/haTZ and is isoenergetic with the new 2C – C_1_ minimum. 2B – C_1_ is the only dihydrate to have a free H atom that does not participate in a hydrogen bond, resulting in three rather than a total of four OH···F/OH···O contacts as in the other BeF_3_^–^(H_2_O)_2_ configurations. The 2B – C_1_ configuration is similar to the global minimum reported for the dihydrate of single halide ions (*X* ^–^(H_2_O)_2_ where *X* = F, Cl, Br, I).^27,52^ While both 2B – C_1_ and 2C – C_1_ were identified with the CCSD­(T) and CCSD­(T):MP2 methods, only 2B – C_1_ was found with MP2, B3LYP-D3BJ and B3LYP as the 2C – C_1_ configuration collapsed to 2B – C_1_. As such, *n*/*a* is reported in each instance in Table 2. Conversely, the ωB97XD and M06-2X methods were able to identify the 2C – C_1_ minimum. However, the 2B – C_1_ structure was not found to be a stationary point (as indicated by *n*/*a* in Table 2) on the ωB97XD and M06-2X PESs as it would collapse to the 2C – C_1_ configuration. Both 2B – C_1_ and 2C – C_1_ are more than 1 kcal mol^–1^ lower in energy than the previously reported 2D – C_2*v*_ structure which, like the 2A – C_2*v*_ global minimum, does not exhibit a water–water OH···O interaction. The 2E – C_*s*_ structure is the highest energy dihydrate minima identified with a Δ*E* of 2.76 kcal mol^–1^ at CCSD­(T)/haTZ and is merely 0.06 kcal mol^–1^ higher in energy than the 2D – C_2*v*_ minimum.

**Table 2:** Relative Electronic Energies (Δ*E* in kcal mol^–1^) for the BeF_3_^–^(H_2_O)_2_ Minima Computed Using Various Methods and the haTZ Basis Set

Structures	CCSD(T)	CCSD(T):MP2	MP2	B3LYP-D3BJ	B3LYP	ωB97XD	M06-2X
2A – C_2*v*_	0.00	0.00	0.00	0.00	0.00	0.00	0.00
2B – C_1_	1.61	1.59	1.35	1.05	0.83	*n*/*a* ^a^	*n*/*a* ^a^
2C – C_1_	1.62	1.57	*n*/*a* ^b^	*n*/*a* ^b^	*n*/*a* ^b^	1.08	1.65
2D – C_2*v*_	2.70	2.65	2.78	2.77	3.20	2.50	2.57
2E – C_*s*_	2.76	2.71	2.83	2.57	3.24	2.44	2.68

a Collapsed to 2C – C_1_.

b Collapsed to 2B – C_1_.

For every BeF_3_^–^(H_2_O)_2_ configuration, the CCSD­(T):MP2 Δ*E*’s were computed to be approximately 0.05 kcal mol^–1^ lower than those obtained with the full CCSD­(T) method. The MP2, B3LYP and M06-2X methods all predict the same energetic ordering established at CCSD­(T) for all of the dihydrate structures with B3LYP-D3BJ and ωB97XD predicting the 2E – C_*s*_ to be nearly 0.2 and 0.1 kcal mol^–1^ below 2D – C_2*v*_, respectively. Compared to the CCSD­(T) Δ*E* values, MP2 relative energies are within 0.08 kcal mol^–1^ for 2D – C_2*v*_ and 2E – C_*s*_ with a larger deviation of 0.26 kcal mol^–1^ for the 2B – C_1_ structure. Similarly, M06-2X observed modest differences of at most 0.13 kcal mol^–1^ for the 2D – C_2*v*_ minimum and below 0.08 kcal mol^–1^ for 2C – C_1_ and 2E – C_*s*_. B3LYP-D3BJ, B3LYP, and ωB97XD Δ*E* values differ on average by 0.4 kcal mol^–1^ from CCSD­(T), with B3LYP providing the largest discrepancies for all configurations (aside from 2C – C_1_) and an absolute maximum deviation of 0.78 kcal mol^–1^ for 2B – C_1_.

### 3.3 Trihydrate Structure and Energetics

Upon the addition of a third water molecule, a total of 11 minima were identified (Figure 3) using ab initio methods, with only the 3C – D_3*h*_ structure having been reported previously.^34^ All 10 local minima have CCSD­(T) relative electronic energies within 5 kcal mol^–1^ of the C_3_ global minimum as reported in Table 3. Unlike with the dihydrate, the global minimum configuration for the trihydrate involves solvent–solvent interactions as the water molecules form a ring above the BeF_3_^–^ ion while also donating a hydrogen to each fluorine atom below (structure 3A – C_3_). This hydration pattern for the global minimum, where a cyclic water trimer donates three hydrogen bonds to a triangular face of the anion, is also observed for BF_4_^–^.^32^ Qualitatively similar (H_2_O)_3_ motifs have been reported for the trihydrates of atomic anions^27,52^ such as Cl^–^ and other polyatomic anions^65–68^ such as ${\mathrm{NO}}_{2}^{-}$ , ${\mathrm{NO}}_{3}^{-}$ , ${\mathrm{CO}}_{3}^{-}$ , ${\mathrm{CO}}_{3}^{2-}$ and CH_3_COO^–^. The *D*_e_ for 3A – C_3_ is 43.55 kcal mol^–1^ using conventional CCSD­(T) and 43.70 kcal mol^–1^ using the 2b:Mb method, thus increasing by a factor of ≈2.7 relative to the monohydrate. At CCSD­(T):MP2/haTZ, the *D*_e_ of the BF_4_^–^(H_2_O)_3_ analog was reported to be 41.24 kcal mol^–1^. The CP procedure lowered the 3A – C_3_ *D*_e_ slightly more than computed for the mono- and dihydrate global minima, resulting in a *D*_e_ of 40.96 kcal mol^–1^.

**Figure 3:**
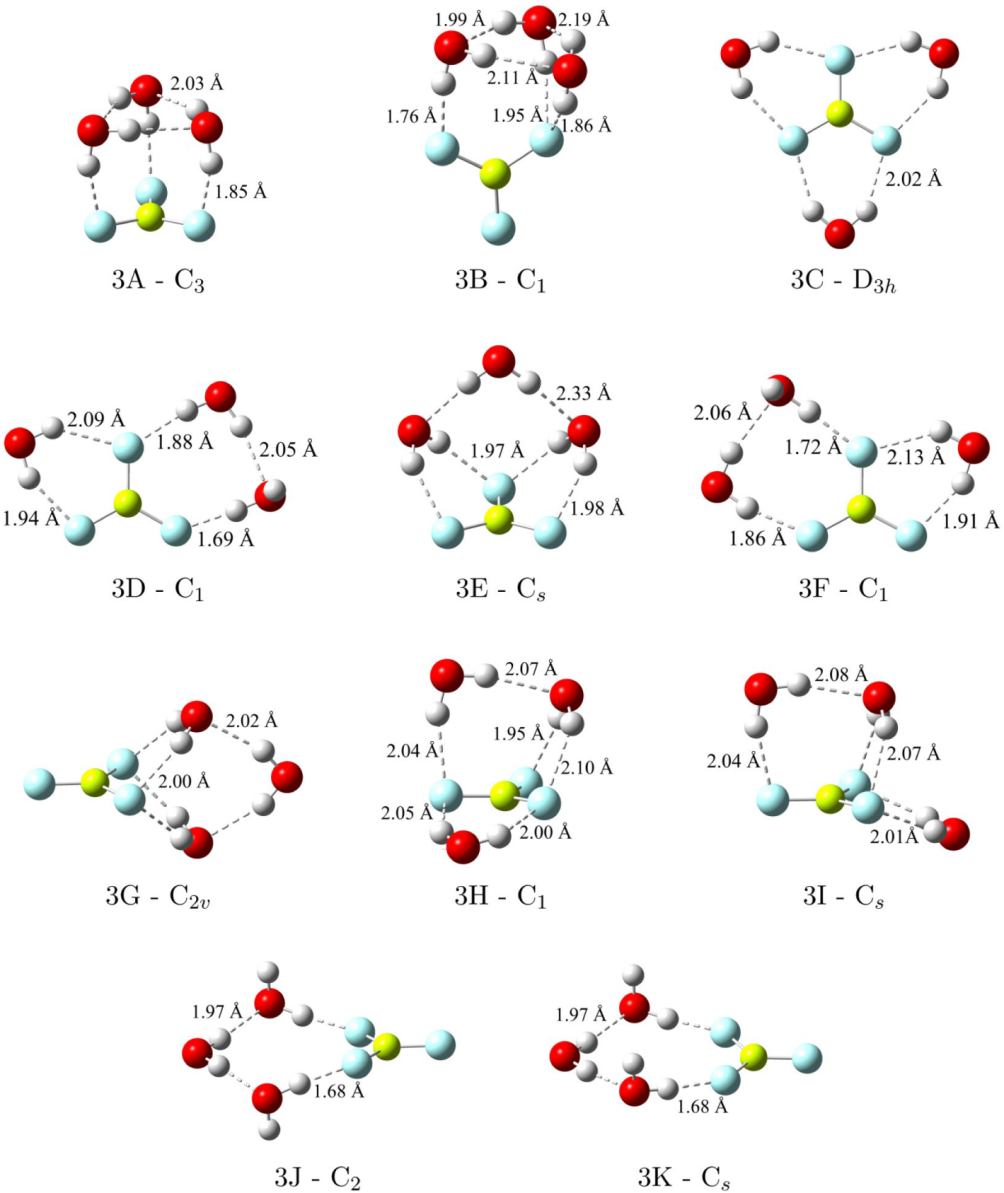
BeF_3_^–^(H_2_O)_3_ minima and their corresponding point-group symmetries along with their symmetry unique R­(OH···F) and R­(OH···O) distances optimized at CCSD­(T)/haTZ.

**Table 3:** Relative Electronic Energies (Δ*E* in kcal mol^–1^) for the BeF_3_^–^(H_2_O)_3_ Minima Computed Using Various Methods and the haTZ Basis Set

Structures	CCSD(T)	CCSD(T):MP2	MP2	B3LYP-D3BJ	B3LYP	ωB97XD	M06-2X
3A – C_3_	0.00	0.00	0.00	0.00	0.00	0.00	0.00
3B – C_1_	0.70	0.71	0.62	1.06	0.41	0.72	0.59
3C – D_3*h*_	0.99	1.18	1.09	2.08	0.49	2.28	0.93
3D – C_1_	1.86	2.02	1.67	2.39	0.55	*n*/*a* ^a^	*n*/*a* ^a^
3E – C_*s*_	1.91	2.06	1.97	2.52	1.74	2.56	2.02
3F – C_1_	2.01	2.16	1.82	2.54	0.70	*n*/*a* ^a^	*n*/*a* ^a^
3G – C_2*v*_	2.30	2.38	2.34	2.97	2.26	2.59	2.01
3H – C_1_	3.52	3.65	3.68	4.39	*n*/*a* ^b^	4.41	3.40
3I – C_*s*_	3.68	3.79	3.85	4.56	3.86	4.45	3.42
3J – C_2_	4.30	4.48	3.80	*n*/*a* ^c^	2.00	*n*/*a* ^c^	*n*/*a* ^c^
3K – C_*s*_	4.50	4.68	3.98	4.67	2.18	5.23	6.61

a Collapsed to C_1_ configurations not found to be stationary points at other levels of theory.

b Collapsed to 3D – C_1_.

c Collapsed to 3G – C_2*v*_.

The 3B – C_1_ configuration has the same ring of water molecules as seen in 3A – C_3_, but now only two of the fluorine atoms form intermolecular contacts with the solvent. One fluorine accepts a single hydrogen bond and the other accepts two. This new configuration is merely 0.70 kcal mol^–1^ higher in energy than 3A – C_3_. 3C – D_3*h*_ shares the same hydrogen bonding topology as seen for the mono- and dihydrate minima (1A – C_2*v*_ and 2A – C_2*v*_), but it no longer has the lowest CCSD­(T)/haTZ electronic energy (with Δ*E* ≈ 1 kcal mol^–1^). The 3E – C_*s*_ structure resembles the 2A – C_2*v*_ configuration but now the two water molecules have raised out of the plane of the BeF_3_^–^ ion and are bridged by the third H_2_O molecule over the top of the anion. This C_*s*_ trihydrate structure has a CCSD­(T) Δ*E* of 1.91 kcal mol^–1^ with the 3D – C_1_ and 3F – C_1_ structures having very similar relative energies of 1.86 and 2.01 kcal mol^–1^, respectively. Both 3D – C_1_ and 3F – C_1_ are extensions of 2B – C_1_ that add a third H_2_O along an adjacent edge of the BeF_3_^–^ anion. The higher-energy arrangement (3F – C_1_) has the H_2_O fragment donating a single hydrogen bond located between the two double-donor H_2_O molecules, but the CCSD­(T)/haTZ electronic energy difference is only 0.15 kcal mol^–1^. As seen with the dihydrate, both ωB97XD and M06-2X result in the 3D – C_1_ and 3F – C_1_ configurations collapsing to different C_1_ structures (variants of the 2C – C_1_ configuration) and is thus indicated in Table 3 by *n*/*a*. These C_1_ structures were not found to be stationary points with any other method.

The 3G – C_2*v*_ can be constructed from the 2D – C_2*v*_ configuration by adding a third water molecule that forms a bridge between the two water molecules to form two OH···O bonds, and it has a CCSD­(T)/haTZ Δ*E* value of 2.30 kcal mol^–1^. With CCSD­(T)/haTZ values of 3.52 and 3.68 kcal mol^–1^, respectively, the 3H – C_1_ and 3I – C_*s*_ minima lie more than 1 kcal mol^–1^ higher in energy than 3G – C_2*v*_. Although both of these trihydrate structures are essentially derivatives of the 2E – C_*s*_ dihydrate structure, we were not able to locate a stationary point for the 3H – C_1_ structure at the B3LYP/haTZ level of theory because the optimizations collapse to 3D – C_1_. While the 3J – C_2_ and 3K – C_*s*_ configurations do not have a BeF_3_^–^ dihydrate analog, they are structurally similar to the C_*s*_ ring local minimum reported for the Cl^–^ trihydrate.^27,52^ Although the CCSD­(T):MP2/haTZ computations indicate 3J – C_2_ has one very small imaginary frequency (1.8*i* cm^–1^), it is included in our analysis because that is plausibly a numerical artifact of the finite difference procedure used to compute the harmonic vibrational frequencies at that level of theory. Similarly, a small imaginary vibrational frequency (5.0*i* cm^–1^) is obtained for 3K – C_*s*_, but this feature likely arises from the choice of numerical integration grid. Optimizations of the 3J – C_2_ structure with the B3LYP-D3BJ, ωB97XD, and M06-2X DFT methods and haTZ basis set collapsed to the 3G – C_2*v*_ structure as indicated in the penultimate row of data in Table 3.

Although some model chemistries employed in this study failed to identify all 11 BeF_3_^–^ trihydrate minima, the CCSD­(T):MP2, B3LYP-D3BJ, ωB97XD and M06-2X methods predicted the same energetic ordering for the BeF_3_^–^(H_2_O)_3_ structures as those obtained from CCSD­(T) computations. Compared to CCSD­(T), the CCSD­(T):MP2 2b:Mb method predicted slightly higher relative electronic energies (by 0.13 kcal mol^–1^ on average). As seen for the dihydrate, Δ*E* values computed using MP2 and M06-2X were within 0.20 and 0.44 kcal mol^–1^ of CCSD­(T) values on average. The same trends observed for the B3LYP-D3BJ, B3LYP, and ωB97XD BeF_3_^–^(H_2_O)_2_ Δ*E* values were also seen with the BeF_3_^–^(H_2_O)_3_ minima, where absolute deviations of 0.64–0.94 kcal mol^–1^ and differences of up to 2.32 kcal mol^–1^ were observed.

### 3.4 Vibrational Frequencies

The symmetric (a_1_) and antisymmetric (b_2_) OH harmonic stretching frequencies for a single isolated water molecule are reported in the first two rows of Tables 4 and 5 at each level of theory tested here. When water molecules bind to the BeF_3_^–^ ion, the formation of OH···F and OH···O hydrogen bonds shift the corresponding OH stretching vibrational frequencies (Δω) of the water molecule to lower energy. The irreducible representations associated with the OH stretching vibrations of the water molecules within each hydrated complex are listed in the “Irreps” columns of Tables 4 and 5 with the irreducible representations for the complex listed to the left and reference mode for an isolated H_2_O monomer to the right. For the BeF_3_^–^(H_2_O)_2_ and BeF_3_^–^(H_2_O)_3_ complexes, where the stretching frequencies no longer directly correlate to the a_1_ and b_2_ irreducible representations, the stretching modes are primarily dominated by a synchronous or asynchronous OH stretching motion that can be used to determine the appropriate H_2_O reference mode for calculating Δω.

**Table 4:** Shifts in the Harmonic OH Stretching Frequencies (Δω in cm^–1^) Induced by Hydrogen Bonding in the BeF_3_^–^(H_2_O)_*n*=1,2_ Minima Relative to the Symmetric (a_1_) and Antisymmetric (b_2_) OH Stretches for an Isolated Water Molecule (ω in cm^–1^) Computed with Various Methods and the haTZ Basis Set

Structures	Irreps^a^		CCSD(T)	CCSD(T):MP2	MP2	B3LYP-D3BJ	B3LYP	ωB97XD	M06-2X
H_2_O	–	a_1_	3814	3814^b^	3825	3801	3801	3882	3872
	–	b_2_	3924	3924^b^	3952	3904	3904	3989	3976
1A – C_2*v*_	a_1_	a_1_	–90	–90^b^	–109	–115	–113	–121	–102
	b_1_	b_2_	–168	–168^b^	–193	–200	–196	–205	–184
2A – C_2*v*_	b_2_	a_1_	–80	–80	–98	–109	–109	–115	–91
	a_1_	a_1_	–78	–77	–96	–106	–106	–112	–89
	b_2_	b_2_	–138	–137	–158	–158	–153	–163	–153
	a_1_	b_2_	–131	–130	–150	–151	–146	–156	–145
2B – C_1_	a	a_1_	–326	–324	–374	–403	–400	–	–
	a	a_1_	–157	–159	–181	–167	–191	–	–
	a	b_2_	–151	–153	–182	–189	–186	–	–
	a	b_2_	–52	–53	–52	–42	–41	–	–
2C – C_1_	a	a_1_	–208	–203	–	–	–	–300	–177
	a	a_1_	–122	–122	–	–	–	–183	–111
	a	b_2_	–126	–128	–	–	–	–154	–151
	a	b_2_	–94	–95	–	–	–	–81	–133
2D – C_2*v*_	b_1_	a_1_	–68	–68	–82	–85	–82	–90	–78
	a_1_	a_1_	–62	–61	–75	–77	–75	–82	–69
	a_2_	b_2_	–130	–130	–148	–151	–147	–157	–144
	b_2_	b_2_	–116	–115	–133	–136	–133	–142	–127
2E – C_*s*_	a′	a_1_	–121	–122	–140	–148	–140	–151	–136
	a′	a_1_	–108	–108	–125	–130	–127	–134	–121
	a″	b_2_	–171	–170	–193	–197	–190	–201	–189
	a′	b_2_	–152	–154	–175	–185	–177	–188	–168

a Irreducible representations of the OH stretching mode in the complex (left) and reference mode in H_2_O (right).

b CCSD­(T):MP2 ≡ CCSD­(T) for monomers and dimers.

**Table 5:** Shifts in the Harmonic OH Stretching Frequencies (Δω in cm^–1^) for the BeF_3_^–^(H_2_O)_3_ Minima Relative to the OH Symmetric and Antisymmetric Stretching Frequencies an Isolated H_2_O Computed with Various Methods and the haTZ Basis Set

Structures	Irreps^a^		CCSD(T):MP2	MP2	B3LYP-D3BJ	B3LYP	ωB97XD	M06-2X
H_2_O	–	a_1_	3814^b^	3825	3801	3801	3882	3872
	–	b_2_	3924^b^	3952	3904	3904	3989	3976
3A – C_3_	a	a_1_	–196	–223	–235	–220	–232	–209
	e	a_1_	–170	–193	–206	–200	–211	–183
	e	a_1_	–170	–193	–206	–200	–211	–183
	a	b_2_	–212	–243	–254	–244	–257	–230
	e	b_2_	–210	–240	–253	–238	–252	–228
	e	b_2_	–210	–240	–253	–238	–252	–228
3B – C_1_	a	a_1_	–229	–263	–285	–282	–279	–250
	a	a_1_	–156	–183	–209	–199	–192	–173
	a	a_1_	–143	–164	–174	–168	–185	–150
	a	b_2_	–183	–206	–206	–196	–215	–199
	a	b_2_	–175	–200	–201	–191	–209	–187
	a	b_2_	–147	–165	–170	–163	–163	–162
3C – D_3*h*_	e′	a_1_	–58	–73	–77	–75	–83	–68
	e′	a_1_	–58	–73	–77	–75	–83	–68
	$a_{1}^{\prime }$	a_1_	–53	–68	–72	–70	–78	–63
	$a_{2}^{\prime }$	b_2_	–122	–141	–146	–142	–151	–136
	e′	b_2_	–112	–131	–135	–132	–141	–126
	e′	b_2_	–112	–131	–135	–132	–141	–126
3D – C_1_	a	a_1_	–284	–328	–358	–353	–	–
	a	a_1_	–119	–140	–149	–145	–	–
	a	a_1_	–78	–98	–111	–115	–	–
	a	b_2_	–151	–179	–192	–186	–	–
	a	b_2_	–114	–131	–127	–120	–	–
	a	b_2_	–50	–50	–41	–40	–	–
3E – C_*s*_	a″	a_1_	–137	–160	–174	–164	–175	–147
	a″	a_1_	–102	–120	–135	–125	–135	–118
	a′	a_1_	–84	–102	–108	–102	–110	–98
	a″	b_2_	–162	–189	–197	–188	–200	–178
	a″	b_2_	–155	–180	–182	–178	–187	–165
	a′	b_2_	–149	–172	–173	–169	–178	–162
3F – C_1_	a	a_1_	–259	–302	–329	–327	–	–
	a	a_1_	–130	–150	–163	–159	–	–
	a	a_1_	–89	–113	–129	–138	–	–
	a	b_2_	–149	–177	–188	–183	–	–
	a	b_2_	–103	–118	–110	–101	–	–
	a	b_2_	–50	–51	–41	–40	–	–
3G – C_2*v*_	a_1_	a_1_	–122	–145	–158	–152	–154	–125
	b_2_	a_1_	–102	–119	–136	–127	–134	–115
	a_1_	a_1_	–78	–94	–96	–94	–103	–87
	b_2_	b_2_	–166	–194	–196	–191	–201	–176
	a_2_	b_2_	–156	–178	–180	–175	–188	–172
	b_1_	b_2_	–139	–160	–162	–158	–170	–151
3H – C_1_	a	a_1_	–116	–139	–153	–	–155	–128
	a	a_1_	–97	–115	–128	–	–131	–105
	a	a_1_	–60	–76	–81	–	–85	–68
	a	b_2_	–139	–157	–158	–	–165	–158
	a	b_2_	–131	–149	–144	–	–151	–142
	a	b_2_	–117	–136	–139	–	–143	–129
3I – C_*s*_	a′	a_1_	–106	–123	–133	–127	–139	–115
	a′	a_1_	–83	–97	–99	–95	–104	–93
	a′	a_1_	–63	–78	–81	–79	–87	–73
	a′	b_2_	–143	–165	–173	–166	–179	–158
	a″	b_2_	–132	–151	–153	–148	–159	–147
	a″	b_2_	–119	–137	–139	–134	–144	–130
3J – C_2_	b	a_1_	–307	–353	–	–378	–	–
	a	a_1_	–288	–334	–	–357	–	–
	a	a_1_	–143	–173	–	–182	–	–
	b	b_2_	–186	–221	–	–231	–	–
	b	b_2_	–49	–50	–	–38	–	–
	a	b_2_	–49	–49	–	–38	–	–
3K – C_*s*_	a″	a_1_	–309	–356	–388	–380	–378	–346
	a′	a_1_	–289	–335	–364	–357	–356	–325
	a′	a_1_	–143	–172	–186	–181	–187	–150
	a″	b_2_	–184	–219	–238	–229	–239	–201
	a″	b_2_	–46	–47	–36	–37	–41	–45
	a′	b_2_	–46	–46	–36	–36	–40	–44

a Irreducible representations of the OH stretching mode in the complex (left) and reference mode in H_2_O (right).

b CCSD­(T):MP2 ≡ CCSD­(T) for monomers and dimers.

In Table 4, the CCSD­(T)/haTZ symmetric OH stretching frequency for the 1A – C_2*v*_ monohydrate structure is 90 cm^–1^ lower than what was computed for the isolated H_2_O monomer, while the magnitude of the shift associated with the antisymmetric frequency is nearly two times larger (168 cm^–1^). The latter CCSD­(T) result for BeF_3_^–^ continues the pattern observed for the monohydrates of BF_4_^–^, SiF_5_^–^ and PF_6_^–^, in which the magnitude of the maximum frequency shift (Δω­(OH)_max_ = 110, 88, and 72 cm^–1^, respectively) decreases as the number of F atoms increases (similar to *D*_e_).^31–33^ For the monohydrate minimum, MP2 and the DFT methods predict larger shifts, ranging from −102 to −121 cm^–1^ for the symmetric mode and −184 to −205 cm^–1^ for the antisymmetric mode. Shifts computed with the M06-2X and MP2 methods agree most closely with CCSD­(T) shifts, while ωB97XD predicted shifts nearly 40 cm^–1^ larger.

As a second water molecule is introduced, the 2A – C_2*v*_ and 2D – C_2*v*_ structures that have water molecules binding to different edges of BeF_3_^–^ exhibit CCSD­(T) vibrational frequency shifts similar to the monohydrate (1A – C_2*v*_) but lower in magnitude by 10–52 cm^–1^. The CCSD­(T) Δω­(OH)_max_ of the BeF_3_^–^(H_2_O)_2_ global minimum with strictly solvent–solute contacts has a magnitude of 138 cm^–1^ and follows the general trend seen with the monohydrates, where the magnitude of this quantity decreases from PF_6_^–^ to BeF_3_^–^. The CCSD­(T) |Δω­(OH)_max_| of the dihydrates is 94 cm^–1^ for BF_4_^–^, 83 cm^–1^ for SiF_5_^–^ and 63 cm^–1^ for PF_6_^–^.^31–33^ Shifts computed for the 2B – C_1_, 2C – C_1_ and 2E – C_*s*_ configurations with water–water contacts are significantly larger than those without, specifically with respect to the synchronous OH stretching modes associated with the OH···O interaction. The 2B – C_1_ structure with one free hydrogen has the largest synchronous and smallest asynchronous Δω values (−326 and −52 cm^–1^, respectively) of all the dihydrate configurations. The 2E – C_*s*_ configuration is the only configuration where each hydrogen is bound to a different hydrogen bond acceptor (i.e., each F or O atom is accepting only one hydrogen bond) and has OH stretching frequency shifts ranging from −108 to −171 cm^–1^ at CCSD­(T)/haTZ. The maximum OH Δω values for the corresponding solvent–solvent conformations reported for the BF_4_^–^, SiF_5_^–^ and PF_6_^–^ ions range from −151 to −100 cm^–1^, following the aforementioned trend. For the BeF_3_^–^ dihydrate systems, the data Table 4 and the Supporting Information reveal that the CCSD­(T):MP2 method gives harmonic vibrational frequencies that are typically within a few cm^–1^ of the CCSD­(T) values (with 2C – C_1_ having the largest maximum absolute deviation or MAD of 5.4 cm^–1^ and average absolute deviation or AAD of 1.0 cm^–1^). As such, CCSD­(T):MP2 frequencies were computed for the BeF_3_^–^(H_2_O)_3_ complexes and used as the reference values in lieu of CCSD­(T) frequencies. The Δω values calculated with the MP2 and DFT methods were consistently larger in comparison to CCSD­(T), with AADs from 16.3 to 33.1 cm^–1^ and MADs as large as 92 cm^–1^ (ωB97XD). As with the monohydrate, the M06-2X and MP2 methods provided shifts closer to the CCSD­(T) values than the other methods (excluding 2b:Mb) and ωB97XD had the most significant deviations.

Frequency shifts for the BeF_3_^–^(H_2_O)_3_ systems are found in Table 5. All of the dihydrate structures include at least one water–water interaction with the exception of 3C – D_3*h*_, which has the smallest Δω­(OH)_max_ of −122 cm^–1^ at the CCSD­(T):MP2/haTZ level. The maximum frequency shifts tend to be significantly larger for trihydrate structures having OH···O hydrogen bonds between the water molecules. The two minima with the lowest electronic energies essentially have a water trimer interacting with a face or an edge of the BeF_3_^–^ anion, and they exhibit maximum shifts in the OH stretching frequencies of −212 cm^–1^ for 3A – C_3_ and −229 cm^–1^ for 3B – C_1_, respectively. The CCSD­(T):MP2/haTZ maximum frequency shift reported for the analogous C_3_ configuration of BF_4_^–^(H_2_O)_3_ is −186 cm^–1^.^32^ As with the 2B – C_1_ dihydrate structure, trihydrate structures with 1 or more free H atoms exhibit the largest shifts of the OH stretching frequencies. Structures 3D – C_1_ and 3F – C_1_, have 1 free H atom and Δω­(OH)_max_ values of −284 and −259 cm^–1^, respectively, at the CCSD­(T):MP2/haTZ level of theory. The higher-energy 3J – C_2_ and 3K – C_*s*_ configurations have 2 free H atoms and display the largest CCSD­(T):MP2/haTZ Δω­(OH)_*max*_ values (with magnitudes exceeding 300 cm^–1^). The remaining configurations (3E – C_*s*_, 3G – C_2*v*_, 3H – C_1_ and 3I – C_*s*_) have |Δω­(OH)_max_| values between 143 cm^–1^ and 166 cm^–1^, larger than the 3C – D_3*h*_ structure with only OH···F contacts but smaller than the configurations with free H atoms or water trimer interactions. In comparison to CCSD­(T):MP2, the OH stretching frequency shift obtained with the MP2 and DFT methods were notably larger as observed for the mono- and dihydrates. The shifts calculated for the BeF_3_^–^(H_2_O)_3_ structures using M06-2X and MP2 were more consistent with the CCSD­(T):MP2 shifts (AADs of 13.2 and 22.3 cm^–1^, respectively) than the other methods, while shifts computed with B3LYP-D3BJ had the largest MAD (79 cm^–1^).

The considerable variation in harmonic OH stretching frequencies of the di- and trihydrate systems naturally leads to different zero-point vibrational energy (ZPVE) corrections to the electronic energies of the isomers, which are shown in the first column of data in Table 6 for the CCSD­(T):MP2/haTZ level of theory. In contrast, the ZPVE from the HOH bending vibrations of the water molecules is remarkably uniform (4.90 ± 0.01 kcal mol^–1^ for BeF_3_^–^(H_2_O)_2_ and 7.35 ± 0.02 kcal mol^–1^ for BeF_3_^–^(H_2_O)_3_). Although the remaining modes have significantly lower harmonic vibrational frequencies (below 1200 cm^–1^) and account for less than a third of the total ZPVE, they exhibit the largest variation between the BeF_3_^–^(H_2_O)_*n*=2,3_ isomers for given value of *n*. For the trihydrates, the values in third column of data in Table 6 differ by as much 2.83 kcal mol^–1^ (roughly 3 times greater than the largest difference for the OH stretch ZPVE contributions from the first data column).

**Table 6:** Zero-Point Vibrational Energies (ZPVE), ZPVE Corrections (δZPVE) and ZPVE Corrected Relative Energies (Δ*E*_0_) for BeF_3_^–^(H_2_O)_*n*=2,3_ from CCSD­(T):MP2/haTZ Harmonic Vibrational Frequencies with All Values in kcal mol^–1^

Zero-Point Vibrational Energy (ZPVE)						
Structures	OH^a^	HOH^b^	Other^c^	Total	δZPVE	Δ*E*_0_
2A – C_2*v*_	21.52	4.91	10.53	36.96	0.00	0.00
2B – C_1_	21.14	4.90	11.02	37.06	+0.11	+1.70
2C – C_1_	21.34	4.90	10.87	37.11	+0.15	+1.72
2D – C_2*v*_	21.59	4.89	10.31	36.80	–0.16	+2.50
2E – C_*s*_	21.33	4.91	11.19	37.44	+0.48	+3.19
3A – C_3_	31.52	7.37	15.40	54.29	0.00	0.00
3B – C_1_	31.71	7.37	14.76	53.84	–0.45	+0.25
3C – D_3*h*_	32.45	7.34	12.54	52.33	–1.95	–0.77
3D – C_1_	32.05	7.34	13.23	52.62	–1.66	+0.35
3E – C_*s*_	32.06	7.34	13.94	53.35	–0.94	+1.12
3F – C_1_	32.07	7.34	13.17	52.58	–1.71	+0.46
3G – C_2*v*_	32.10	7.35	13.78	53.22	–1.06	+1.32
3H – C_1_	32.24	7.34	13.26	52.85	–1.44	+2.21
3I – C_*s*_	32.26	7.34	13.23	52.83	–1.46	+2.33
3J – C_2_	31.73	7.33	13.50	52.56	–1.73	+2.75
3K – C_*s*_	31.73	7.33	13.47	52.53	–1.75	+2.92

a From OH stretches (2*n* highest frequencies).

b From HOH bends (next *n* highest frequencies).

c From all other modes (6*n* + 6), all with ω < 1200 cm^–1^.

When the total CCSD­(T):MP2/haTZ ZPVE contributions are combined and used to compute corrections (δZPVE) to the relative energies with respect to 2A – C_2*v*_ and 3A – C_3_, the dominant contributions from the OH stretching frequencies and the lower energy modes (below 1200 cm^–1^) cancel to some extent. This yields ZPVE corrected relative energies (Δ*E*_0_) for the dihydrate that are typically within ±0.16 kcal mol^–1^ of Δ*E* and still within ±0.48 kcal mol^–1^ for the lone BeF_3_^–^(H_2_O)_2_ structure with a somewhat larger δZPVE correction (2E – C_*s*_).

For the trihydrate CCSD­(T):MP2/haTZ δZPVE values in Table 6, the contribution from the OH stretches consistently increases the relative energies with respect to 3A – C_3_ (by +0.19 to +0.93 kcal mol^–1^). However, that is always offset by even larger decreases from the “other” vibrational frequencies below 1200 cm^–1^ (by −0.64 to −2.86 kcal mol^–1^). As a result, every δZPVE value is negative for the BeF_3_^–^(H_2_O)_3_ isomers in the penultimate column of Table 6, ranging from −0.45 kcal mol^–1^ for 3B – C_1_ to −1.95 kcal mol^–1^ for 3C – D_3*h*_. The latter ZPVE correction is large enough to shift the CCSD­(T):MP2 energy of 3C – D_3*h*_ below that of 3A – C_3_ by −0.77 kcal mol^–1^ (last column of Table 6). Interestingly, the ZPVE correction also changes the energetic ordering of the lowest-energy trihydrate isomers of the isoelectronic ${\mathrm{NO}}_{3}^{-}$ anion.^65^ The relative energies of the other trihydrate minima are effectively compressed by δZPVE, with 3B – C_1_, 3D – C_1_ and 3F – C_1_ having Δ*E*_0_ values of only +0.25, +0.35 and +0.46 kcal mol^–1^, respectively. Δ*E*_0_ ranged from +1.12 to +2.92 kcal mol^–1^ for the remaining BeF_3_^–^(H_2_O)_3_ structures. (See Supporting Information for MP2 and DFT δZPVE and Δ*E*_0_ values which mirror the trends for the CCSD­(T):MP2/haTZ data presented here.).

## 4 Conclusions

The beryllium trifluoride anion (BeF_3_^–^) was microhydrated with one to three water molecules (BeF_3_^–^(H_2_O)_*n*=1–3_). The CCSD­(T) and MP2 ab initio methods as well as four DFT approaches (B3LYP-D3BJ, B3LYP, ωB97XD and M06-2X) were used in conjunction with a correlation-consistent triple-ζ basis set augmented with diffuse functions on all non-hydrogen atoms (haTZ). The 2-body:Many-body QM:QM technique denoted CCSD­(T):MP2 was found to accurately reproduce the conventional CCSD­(T) relative and dissociation electronic energies as well as harmonic vibrational frequencies for the di- and trihydrate systems at significantly reduced computational cost. Three new minima were identified for BeF_3_^–^(H_2_O)_2_ along with ten new minima for BeF_3_^–^(H_2_O)_3_, and several of these new hydration motifs for BeF_3_^–^ resemble those reported for the di- and trihydrates of the isoelectronic ${\mathrm{NO}}_{3}^{-}$ and ${\mathrm{CO}}_{3}^{2-}$ anions.^65,66^ The BeF_3_^–^ mono- and dihydrate global minima (1A – C_2*v*_ and 2A – C_2*v*_) and the trihydrate local minimum (3C – D_3*h*_) have been previously identified^34^ at the MP2/haTZ level of theory, and form double ionic hydrogen bonds (DIHBs) between the water molecule(s) and the planar BeF_3_^–^ ion.

The hydration motif for the global minimum of BeF_3_^–^(H_2_O)_2_ is fundamentally different than for the lowest-energy dihydrate structures of three closely related anions (BF_4_^–^, SiF_5_^–^ and PF_6_^–^), which exhibit a hydrogen bond between the pair of H_2_O solvent molecules. For the dihydrates of BF_4_^–^, SiF_5_^–^ and PF_6_^–^, the configurations having only DIHB contacts are higher-energy local minima.^31–33^ The addition of a third water molecule changes the hydration pattern around BeF_3_^–^ in the global minimum from solely solvent–solute (water–ion) interactions to a hydrogen-bonding network that also includes solvent–solvent (water–water) contacts. The newly identified BeF_3_^–^(H_2_O)_3_ configuration with the lowest CCSD­(T)/haTZ electronic energy (3A – C_3_) has a cyclic water trimer over the triangular face of BeF_3_^–^ that forms three OH···F hydrogen bonds with the ion. This hydration motif is the same as that recently reported for for BF_4_^–^(H_2_O)_3_.^32^ When harmonic ZPVE is included, however, the planar isomer with 3 identical DIHBs (3C – D_3*h*_) becomes the lowest-energy structure, further highlighting the competition between solvent–solute and solvent–solvent interactions.

The CCSD­(T)/haTZ electronic dissociation energy for the BeF_3_^–^ monohydrate global minimum is approximately 16 kcal mol^–1^, extending the trend in which *D*_e_ decreases as the number of F atoms increases for the monohydrates of BF_4_^–^, SiF_5_^–^ and PF_6_^–^ (approximately 13, 12, and 11 kcal mol^–1^, respectively).^31–33^ When additional water molecules hydrate BeF_3_^–^, the interaction is attenuated, and the increase in *D*_e_ is less than pairwise additive. The CCSD­(T)/haTZ dissociation energies are approximately 30 kcal mol^–1^ for *n* = 2 (2A – C_2*v*_) and 44 kcal mol^–1^ for *n* = 3 (3A – C_3_). This differs from the cooperative effects observed in the *D*_e_ values for the dihydrate global minima of BF_4_^–^, SiF_5_^–^, and PF_6_^–^ (as well the higher-order hydrates of BF_4_^–^).

The formation of hydrogen bonds in these complexes (OH···F and/or OH···O) results in a significant decrease in the harmonic OH stretching frequencies of H_2_O and is largely dependent on the hydration pattern adopted. Frequency shifts for BeF_3_^–^(H_2_O)_*n*_ were calculated with respect to the symmetric and antisymmetric stretching modes of a single H_2_O molecule. For the symmetric structures exhibiting only DIHB contacts, the largest shifts at CCSD­(T)/haTZ were −168 cm^–1^ for *n* = 1 (1A – C_2*v*_), −138 cm^–1^ for *n* = 2 (2A – C_2*v*_), and −122 cm^–1^ for *n* = 3 (3C – D_3*h*_). For the 3A – C_3_ trihydrate global minimum, where hydrogen bonding between the water molecules occurs, the largest shift exceeds 200 cm^–1^. This increase in magnitude for the frequency shifts of water–water configurations relative to the DIHB only configurations was also seen for the PF_6_^–^, SiF_5_^–^ and BF_4_^–^ ions. As with *D*_e_, these new result extend a previously observed trend that the maximum frequency shifts for *n* = 1 and *n* = 2 decrease as the number of F atoms in the ion increases from BF_4_^–^ to SiF_5_^–^ and PF_6_^–^. Shifts with magnitudes surpassing 300 cm^–1^ were calculated in the di- and trihydrate structures having at least one free H atom that does not participate in hydrogen bonding. Overall, these findings suggest that water–water interactions must be considered along with water–anion interactions when attempting to characterize the hydration of BeF_3_^–^, which has also recently been recognized for related ions such as BF_4_^–^, SiF_5_^–^ and PF_6_^–^.

## Supplementary Material



## Notes

ZPVE energetics (δZPVE and Δ*E*_0_); cartesian coordinates of optimized structures; vibrational frequencies and available IR intensities (PDF)
