# Supplementary material for: Explicit Hydration of the Beryllium Trifluoride Anion with One to Three Water Molecules: BeF3 –(H2O) n=1–3
Source: J Phys Chem A. 2026 Apr 21;130(18):3611–22. doi: 10.1021/acs.jpca.6c01068 (PMC13158993; doi:10.1021/acs.jpca.6c01068)
Supplement: Supplementary file 1 [file jp6c01068_si_001.pdf]

Supporting Information for:

Explicit Hydration of the Beryllium Trifluoride Anion with One to Three Water Molecules: $BeF_3^-(H_2O)_{n=1-3}$

Kayleigh R. Autry[†] and Gregory S. Tschumper[*,‡]

[†]Department of Chemistry and Biochemistry, The University of Alabama, Tuscaloosa, Alabama 35487 USA

[‡]Department of Chemistry, Missouri University of Science and Technology, Rolla, Missouri 65409 USA

I. ENERGETICS

TABLE S1. Harmonic ZPVE correction contribution to the relative energies (δZPVE in kcal mol^{-1}) for the $BeF_3^-(H_2O)_{n=2,3}$ minima computed using various methods and the haTZ basis set.

Structures	CCSD(T):MP2	MP2	B3LYP-D3BJ	B3LYP	ωB97XD	M06-2X
2A - C_{2v}	0.00	0.00	0.00	0.00	0.00	0.00
2B - C_1	0.11	0.13	0.10	0.14	n/a^a	n/a^a
2C - C_1	0.15	n/a^b	n/a^b	n/a^b	0.29	0.19
2D - C_{2v}	-0.16	-0.15	-0.17	-0.21	-0.24	-0.12
2E - C_s	0.48	0.48	0.45	0.41	0.57	0.41
3A - C_3	0.00	0.00	0.00	0.00	0.00	0.00
3B - C_1	-0.45	-0.45	-0.45	-0.45	-0.29	-0.34
3C - D_{3h}	-1.95	-1.92	-1.93	-1.87	-2.01	-1.52
3D - C_1	-1.66	-1.61	-1.67	-1.57	n/a^c	n/a^c
3E - C_s	-0.94	-0.93	-0.94	-0.92	-0.74	-0.73
3F - C_1	-1.71	-1.65	-1.71	-1.62	n/a^c	n/a^c
3G - C_{2v}	-1.06	-1.05	-1.05	-1.06	-1.06	-0.79
3H - C_1	-1.44	-1.41	-1.46	n/a^d	-1.37	-1.18
3I - C_s	-1.46	-1.43	-1.47	-1.48	-1.42	-1.13
3J - C_2	-1.73	-1.66	n/a^e	-1.59	n/a^e	n/a^e
3K - C_s	-1.75	-1.70	-1.81	-1.64	-1.72	-1.99

[a]Collapsed to 2C - C_1.
[b]Collapsed to 2B - C_1.
[c]Collapsed to C_1 configurations not found to be stationary points at other levels of theory.
[d]Collapsed to 3D - C_1.
[e]Collapsed to 3G - C_{2v}.

TABLE S2. Harmonic ZPVE-corrected relative energies (ΔE_0 in kcal mol^{-1}) for the $BeF_3^-(H_2O)_{n=2,3}$ minima computed using various methods and the haTZ basis set.

Structures	CCSD(T):MP2	MP2	B3LYP-D3BJ	B3LYP	ωB97XD	M06-2X
2A - C_{2v}	0.00	0.00	0.00	0.00	0.00	0.00
2B - C_1	1.70	1.47	1.16	0.96	*n/a*[a]	*n/a*[a]
2C - C_1	1.72	*n/a*[b]	*n/a*[b]	*n/a*[b]	1.37	1.84
2D - C_{2v}	2.50	2.62	2.60	2.99	2.27	2.45
2E - C_s	3.19	3.31	3.02	3.65	3.01	3.09
3A - C_3	0.00	0.00	0.00	0.00	0.00	0.00
3B - C_1	0.25	0.17	0.61	-0.04	0.43	0.26
3C - D_{3h}	-0.77	-0.83	0.15	-1.38	0.27	-0.59
3D - C_1	0.35	0.06	0.72	-1.03	*n/a*[c]	*n/a*[c]
3E - C_s	1.12	1.04	1.58	0.82	1.82	1.30
3F - C_1	0.46	0.17	0.83	-0.92	*n/a*[c]	*n/a*[c]
3G - C_{2v}	1.32	1.29	1.93	1.20	1.52	1.22
3H - C_1	2.21	2.27	2.93	*n/a*[d]	3.04	2.22
3I - C_s	2.33	2.42	3.10	2.38	3.03	2.29
3J - C_2	2.75	2.14	*n/a*[e]	0.41	*n/a*[e]	*n/a*[e]
3K - C_s	2.92	2.28	2.86	0.54	3.51	4.63

[a]Collapsed to 2C - C_1.

[b]Collapsed to 2B - C_1.

[c]Collapsed to C_1 configurations not found to be stationary points at other levels of theory.

[d]Collapsed to 3D - C_1.

[e]Collapsed to 3G - C_{2v}.

TABLE S3. Total electronic energies (in hartree) computed using various methods and the haTZ basis set.

Structures	CCSD(T)	CCSD(T):MP2	MP2	B3LYP-D3BJ	B3LYP	ωB97XD	M06-2X
H_2O	-76.341390	–	-76.328054	-76.466563	-76.465989	-76.439774	-76.429950
BeF_3^-	-314.244390	–	-314.223259	-314.732026	-314.729868	-314.626045	-314.627937
1A - C_{2v}	-390.611301	–	-390.576377	-391.224173	-391.219275	-391.091030	-391.085059
2A - C_{2v}	-466.975180	-466.975155	-466.926428	-467.713212	-467.705570	-467.552828	-467.538844
2B - C_1	-466.972613	-466.972614	-466.924283	-467.711531	-467.704251	*n/a*[a]	*n/a*[a]
2C - C_1	-466.972600	-466.972659	*n/a*[b]	*n/a*[b]	*n/a*[b]	-467.551105	-467.536210
2D - C_{2v}	-466.970877	-466.970926	-466.922003	-467.708799	-467.700471	-467.548840	-467.534753
2E - C_s	-466.970788	-466.970836	-466.921911	-467.709120	-467.700406	-467.548935	-467.534574
3A - C_3	-543.337966	-543.338200	-543.275507	-544.202823	-544.189885	-544.015454	-543.9911625
3B - C_1	-543.336844	-543.337076	-543.274513	-544.201130	-544.189239	-544.014300	-543.9902147
3C - D_{3h}	-543.336390	-543.336315	-543.273776	-544.199509	-544.189104	-544.011817	-543.9896841
3D - C_1	-543.335008	-543.334985	-543.272854	-544.199015	-544.189019	*n/a*[c]	*n/a*[c]
3E - C_s	-543.334917	-543.334911	-543.272371	-544.198806	-544.187111	-544.011377	-543.9879242
3F - C_1	-543.334767	-543.334751	-543.272612	-544.198782	-544.188781	*n/a*[c]	*n/a*[c]
3G - C_{2v}	-543.334305	-543.334402	-543.271777	-544.198083	-544.186292	-544.011332	-543.9879568
3H - C_1	-543.332361	-543.332385	-543.269642	-544.195830	*n/a*[d]	-544.008421	-543.9857455
3I - C_s	-543.332100	-543.332166	-543.269373	-544.195553	-544.183735	-544.008355	-543.9857092
3J - C_2	-543.331107	-543.331062	-543.269458	*n/a*[e]	-544.186695	*n/a*[e]	*n/a*[e]
3K - C_s	-543.330796	-543.330745	-543.269160	-544.195377	-544.186420	-544.007119	-543.9806243

[a]Collapsed to 2C - C_1.
[b]Collapsed to 2B - C_1.
[c]Collapsed to C_1 configurations not found to be stationary points at other levels of theory.
[d]Collapsed to 3D - C_1.
[e]Collapsed to 3G - C_{2v}.

TABLE S4. Harmonic ZPVE corrections (in hartree) computed using various methods and the haTZ basis set.

Structures	CCSD(T)	CCSD(T):MP2	MP2	B3LYP-D3BJ	B3LYP	ωB97XD	M06-2X
H_2O	0.021383	–	0.021430	0.021262	0.021262	0.021662	0.021569
BeF_3^-	0.008917	–	0.008815	0.008841	0.008837	0.008754	0.009185
1A - C_{2v}	0.034064	–	0.033911	0.033780	0.033702	0.034013	0.034500
2A - C_{2v}	0.058891	0.058892	0.058700	0.058432	0.058260	0.059017	0.059557
2B - C_1	0.059069	0.059061	0.058904	0.058593	0.058477	n/a^a	n/a^a
2C - C_1	0.059120	0.059137	n/a^b	n/a^b	n/a^b	0.059483	0.059863
2D - C_{2v}	0.058629	0.058643	0.058453	0.058166	0.057933	0.058642	0.059369
2E - C_s	0.059654	0.059661	0.059462	0.059151	0.058921	0.059923	0.060204
3A - C_3	–	0.086512	0.086230	0.085869	0.085495	0.086804	0.086906
3B - C_1	–	0.085792	0.085512	0.085147	0.084784	0.086342	0.086365
3C - D_{3h}	–	0.083400	0.083177	0.082798	0.082512	0.083604	0.084483
3D - C_1	–	0.083860	0.083667	0.083214	0.082994	n/a^c	n/a^c
3E - C_s	–	0.085012	0.084750	0.084376	0.084024	0.085624	0.085738
3F - C_1	–	0.083790	0.083599	0.083148	0.082919	n/a^c	n/a^c
3G - C_{2v}	–	0.084819	0.084556	0.084203	0.083812	0.085107	0.085652
3H - C_1	–	0.084219	0.083980	0.083545	n/a^d	0.084614	0.085028
3I - C_s	–	0.084191	0.083952	0.083532	0.083135	0.084534	0.085098
3J - C_2	–	0.083757	0.083589	n/a^e	0.082955	n/a^e	n/a^e
3K - C_s	–	0.083717	0.083523	0.082984	0.082887	0.084070	0.083739

[a]Collapsed to 2C - C_1.
[b]Collapsed to 2B - C_1.
[c]Collapsed to C_1 configurations not found to be stationary points at other levels of theory.
[d]Collapsed to 3D - C_1.
[e]Collapsed to 3G - C_{2v}.

II. CCSD(T) OPTIMIZED STRUCTURES

TABLE S5. Cartesian coordinates (in bohr) for the $BeF_3{}^-$ monomer at CCSD(T)/aTZ.

Be	−0.00000000	0.00000000	0.00000000
F	−0.00000000	−2.42268050	1.39873524
F	−0.00000000	0.00000000	−2.79747048
F	0.00000000	2.42268050	1.39873524

TABLE S6. Cartesian coordinates (in bohr) for the $BeF_3{}^-(H_2O)_1$ global minimum (1A - C_{2v}) at CCSD(T)/haTZ.

Be	−0.00000000	0.00000000	1.33318382
F	−0.00000000	−0.00000000	4.10433810
F	2.40293037	0.00000000	−0.12470256
F	−2.40293037	−0.00000000	−0.12470256
O	−0.00000000	0.00000000	−4.86760831
H	−1.38674499	−0.00000000	−3.66903325
H	1.38674499	0.00000000	−3.66903325

TABLE S7. Cartesian coordinates (in bohr) for the $BeF_3{}^-(H_2O)_2$ global minimum (2A - C_{2v}) at CCSD(T)/haTZ.

Be	0.00000000	0.00000000	1.09217669
F	0.00000000	0.00000000	−1.72614861
F	0.00000000	2.44034940	2.43689296
F	−0.00000000	−2.44034940	2.43689296
O	0.00000000	5.41102281	−1.99510712
H	0.00000000	5.00286506	−0.20893494
H	0.00000000	3.71488779	−2.67839265
O	−0.00000000	−5.41102281	−1.99510712
H	−0.00000000	−5.00286506	−0.20893494
H	−0.00000000	−3.71488779	−2.67839265

TABLE S8. Cartesian coordinates (in bohr) for the $BeF_3^-(H_2O)_2$ local minimum (2B - C_1) at CCSD(T)/haTZ.

Be	−2.31390927	0.02014915	0.00697094
F	−0.95866924	2.44081429	0.37119984
F	−5.06905226	−0.12801978	−0.07453118
F	−0.74694288	−2.31500652	−0.24782755
O	4.24907156	2.85990323	−0.32410481
O	4.23257001	−2.79198322	0.17767815
H	2.39471133	−2.69084252	−0.06781613
H	2.42676612	2.92442488	−0.08134402
H	4.55000337	1.06317353	−0.47308404
H	4.41865549	−2.51317535	1.96310719

TABLE S9. Cartesian coordinates (in bohr) for the $BeF_3^-(H_2O)_2$ local minimum (2C - C_1) at CCSD(T)/haTZ.

Be	−2.16346766	0.07399697	0.04627793
F	−4.77574501	0.93362477	−0.14120701
F	−0.18380431	1.60935997	1.33895359
F	−1.32733499	−2.39720212	−1.00460942
O	4.42464831	2.57181192	−1.09121254
H	2.76729139	2.65096278	−0.30441545
H	4.71564673	0.76909952	−1.09031868
O	3.47932888	−2.73085405	0.80192490
H	1.84985969	−2.91125457	−0.04622153
H	3.08492059	−1.39452274	1.97752502

TABLE S10. Cartesian coordinates (in bohr) for the $BeF_3^-(H_2O)_2$ local minimum (2D - C_{2v}) at CCSD(T)/haTZ.

F	0.00000000	−0.00000000	−4.46159800
Be	0.00000000	−0.00000000	−1.71091630
O	3.65033362	−0.00000000	3.06504450
O	−3.65033362	0.00000000	3.06504450
F	−0.00000000	−2.38247221	−0.19440682
F	0.00000000	2.38247221	−0.19440682
H	2.70660599	1.39947012	2.36125196
H	2.70660599	−1.39947012	2.36125196
H	−2.70660599	−1.39947012	2.36125196
H	−2.70660599	1.39947012	2.36125196

TABLE S11. Cartesian coordinates (in bohr) for the $BeF_3{}^{-}(H_2O)_2$ local minimum (2E - C_s) at CCSD(T)/haTZ.

O	−4.62679807	−0.90260197	0.00000000
F	0.31256029	−3.29991904	0.00000000
Be	1.60988445	−0.83601210	0.00000000
F	2.15291691	0.50123008	2.40000296
F	2.15291691	0.50123008	−2.40000296
H	−3.84270008	0.75150309	0.00000000
H	−3.18421423	−2.03348101	−0.00000000
O	−1.28926194	3.78039280	−0.00000000
H	−0.26885158	3.19714940	−1.40794561
H	−0.26885158	3.19714940	1.40794561

TABLE S12. Cartesian coordinates (in bohr) for the $BeF_3{}^{-}(H_2O)_3$ global minimum (3A - C_3) at CCSD(T)/haTZ.

Be	0.00000000	0.00000000	−2.40617285
F	2.79205893	0.02285745	−2.31356474
F	−1.41582460	2.40656524	−2.31356474
F	−1.37623434	−2.42942269	−2.31356474
O	3.19854163	0.20315694	2.93334197
O	−1.77520989	2.66843983	2.93334197
O	−1.42333174	−2.87159678	2.93334197
H	3.46102161	0.14911304	1.11340101
H	−1.85964649	2.92277612	1.11340101
H	−1.60137512	−3.07188916	1.11340101
H	1.86837720	1.45520743	3.11726457
H	−2.19443520	0.89045840	3.11726457
H	0.32605800	−2.34566583	3.11726457

TABLE S13. Cartesian coordinates (in bohr) for the $BeF_3{}^{-}(H_2O)_3$ local minimum (3B - C_1) at CCSD(T)/haTZ.

F	5.51809969	−0.78103937	0.05089412
Be	2.86265361	−0.07375102	−0.01999167
F	2.00063585	2.58912296	−0.15130985
F	0.82109471	−2.03755912	0.02780232
O	−3.57309823	−0.99258638	3.01682133
H	−3.60023575	0.70059997	2.30160449
H	−1.94324269	−1.60109220	2.43902017
O	−3.10014704	3.26120346	−0.40593160
H	−1.25596779	3.23766380	−0.31596118
H	−3.46177064	1.91173865	−1.58882169
O	−3.78497758	−1.95639681	−2.50850273
H	−2.01249983	−2.14567836	−2.05780492
H	−4.55840358	−2.07307809	−0.85539543

TABLE S14. Cartesian coordinates (in bohr) for the $BeF_3{}^{-}(H_2O)_3$ local minimum (3C - D_{3h}) at CCSD(T)/haTZ.

Be	0.00000000	−0.00000000	0.00000000
F	0.00000000	−2.42181728	−1.39823686
F	0.00000000	−0.00000000	2.79647372
F	0.00000000	2.42181728	−1.39823686
H	0.00000000	−1.39724724	−5.08061163
H	0.00000000	1.39724724	−5.08061163
O	0.00000000	0.00000000	−6.25965604
H	0.00000000	−3.70131512	3.75035742
H	0.00000000	−5.09856236	1.33025421
H	0.00000000	5.09856236	1.33025421
H	0.00000000	3.70131512	3.75035742
O	0.00000000	5.42102115	3.12982802
O	0.00000000	−5.42102115	3.12982802

TABLE S15. Cartesian coordinates (in bohr) for the $BeF_3^-(H_2O)_3$ local minimum (3D - C_1) at CCSD(T)/haTZ.

O	−6.14851955	−1.20257567	−0.29241516
F	3.49706827	−2.50896526	0.17038455
Be	1.02012205	−1.25483316	0.10752827
F	0.87816505	1.54221039	−0.03617319
F	−1.35844997	−2.72976722	0.15280646
O	−3.90568713	3.95454222	0.29101626
H	−6.27101154	−1.03381451	−2.09680652
H	−4.42916020	−1.84705317	−0.04537900
H	−2.18534226	3.32556335	0.19022636
H	−4.88672095	2.41177879	0.32772281
O	6.34789659	1.98381807	−0.27464531
H	4.66064275	2.68272167	−0.24939745
H	5.94221419	0.20419327	−0.11742707

TABLE S16. Cartesian coordinates (in bohr) for the $BeF_3^-(H_2O)_3$ local minimum (3E - C_s) at CCSD(T)/haTZ.

Be	−0.00743252	−2.39142179	0.00000000
F	1.18940365	−2.95028924	2.44709454
F	1.18940365	−2.95028924	−2.44709454
F	−2.49252055	−1.03848577	0.00000000
O	−0.58105339	1.81212391	4.05704119
H	0.46277372	0.30674743	4.12516080
H	−1.86966806	1.24256579	2.88654718
O	−0.58105339	1.81212391	−4.05704119
H	0.46277372	0.30674743	−4.12516080
H	−1.86966806	1.24256579	−2.88654718
O	1.36712700	5.23888796	0.00000000
H	0.88501422	4.21456884	−1.44091820
H	0.88501422	4.21456884	1.44091820

TABLE S17. Cartesian coordinates (in bohr) for the $BeF_3^-(H_2O)_3$ local minimum (3F - C_1) at CCSD(T)/haTZ.

O	−3.69148320	3.95495326	0.18809596
F	3.57437144	−2.45125708	0.05117698
Be	0.99711043	−1.41301771	0.04787572
F	−1.27282208	−2.99569378	0.24404607
F	0.69097223	1.39509422	−0.13146853
O	−6.20547522	−1.09236189	−0.34447316
H	−3.94300916	3.92704083	1.98699552
H	−2.07017498	3.08879845	−0.02192600
H	−4.60256548	−1.96571300	−0.14543877
H	−5.68065174	0.65653866	−0.43175444
O	6.16050927	2.18496632	−0.13581710
H	5.81876312	0.38510854	−0.06188060
H	4.44971955	2.81644446	−0.20369396

TABLE S18. Cartesian coordinates (in bohr) for the $BeF_3^-(H_2O)_3$ local minimum (3G - C_{2v}) at CCSD(T)/haTZ.

O	−3.35017798	0.00000000	−2.09388894
F	0.00000000	−2.37137863	1.39687537
Be	0.00000000	0.00000000	2.93540116
F	0.00000000	0.00000000	5.67758438
F	0.00000000	2.37137863	1.39687537
O	3.35017798	0.00000000	−2.09388894
H	−2.54398039	1.40340869	−1.23594658
H	−2.54398039	−1.40340869	−1.23594658
H	2.54398039	1.40340869	−1.23594658
H	2.54398039	−1.40340869	−1.23594658
O	0.00000000	0.00000000	−6.53944199
H	−1.41472110	0.00000000	−5.37433219
H	1.41472110	0.00000000	−5.37433219

TABLE S19. Cartesian coordinates (in bohr) for the $BeF_3^-(H_2O)_3$ local minimum (3H - C_1) at CCSD(T)/haTZ.

O	−4.08792181	−0.60631290	−2.30103776
F	1.11221630	−2.07432463	−1.50903589
Be	0.23808578	−1.18728665	1.01015996
F	1.45647227	1.05763474	2.14462109
F	−1.96479721	−2.41787729	2.18242092
O	−2.43786931	4.40232572	−0.10714138
H	−2.46550156	−1.13569808	−2.95964656
H	−4.08079020	−1.42392848	−0.65835789
H	−1.03943870	3.67109184	0.81747106
H	−3.16830273	2.95736620	−0.96165790
O	5.76546118	0.64203035	−1.16641433
H	4.79211156	1.35161397	0.20850195
H	4.51600445	−0.49378242	−1.86991563

TABLE S20. Cartesian coordinates (in bohr) for the $BeF_3^-(H_2O)_3$ local minimum (3I - C_s) at CCSD(T)/haTZ.

Be	−0.13776075	1.48406633	0.00000000
F	2.11369794	3.09053700	0.00000000
F	−1.29046806	0.53743203	2.38549429
F	−1.29046806	0.53743203	−2.38549429
O	0.87579486	−3.83427510	0.00000000
H	0.07434987	−2.99732803	−1.41654981
H	0.07434987	−2.99732803	1.41654981
O	−6.02610907	−0.50730152	0.00000000
H	−4.87039125	−0.26600487	−1.39678625
H	−4.87039125	−0.26600487	1.39678625
O	5.79923045	−0.93689324	0.00000000
H	4.90098086	0.65991248	0.00000000
H	4.43207344	−2.15246627	0.00000000

TABLE S21. Cartesian coordinates (in bohr) for the $BeF_3{}^{-}(H_2O)_3$ local minimum (3J - C_2) at CCSD(T)/haTZ.

Be	0.00000000	0.00000000	3.26952167
F	0.00000000	0.00000000	6.02378190
F	−2.33789140	0.54451381	1.80741059
F	2.33789140	−0.54451381	1.80741059
O	−4.14504587	−0.24184992	−2.82150732
O	4.14504587	0.24184992	−2.82150732
H	3.47277578	−0.15113815	−1.13857308
H	4.26155824	2.05427764	−2.80733949
H	−3.47277578	0.15113815	−1.13857308
H	−4.26155824	−2.05427764	−2.80733949
O	0.00000000	0.00000000	−6.47805123
H	−1.43104089	0.09934656	−5.33554911
H	1.43104089	−0.09934656	−5.33554911

TABLE S22. Cartesian coordinates (in bohr) for the $BeF_3{}^{-}(H_2O)_3$ local minimum (3K - C_s) at CCSD(T)/haTZ.

Be	0.06807120	3.28134130	0.00000000
F	0.04034904	6.03596365	0.00000000
F	0.05911427	1.82291381	2.40222182
F	0.05911427	1.82291381	−2.40222182
O	−0.18654865	−2.86249458	4.19952870
O	−0.18654865	−2.86249458	−4.19952870
H	0.01966744	−1.14873353	−3.52122929
H	−1.97882018	−2.99202769	−4.46012432
H	0.01966744	−1.14873353	3.52122929
H	−1.97882018	−2.99202769	4.46012432
O	0.35098039	−6.43461740	0.00000000
H	0.33564040	−5.29478095	1.43640067
H	0.33564040	−5.29478095	−1.43640067

III. CCSD(T) AND CCSD(T):MP2 VIBRATIONAL FREQUENCIES

TABLE S23. Harmonic vibrational frequencies (ω in cm^{-1}) along with their corresponding irreducible representations and infrared intensities (IR in km mol^{-1}) computed for the BeF_3^- monomer at CCSD(T)/aTZ.

Irrep.	ω	IR
a_1'	1069.1	792.3
a_1'	598.9	0.0
a_1'	321.9	33.2
a_2'	1069.1	792.3
a_2'	321.9	33.2
a_2''	533.3	427.0

TABLE S24. Harmonic vibrational frequencies (ω in cm^{-1}) along with their corresponding irreducible representations and infrared intensities (IR in km mol^{-1}) computed for the $BeF_3^-(H_2O)_1$ global minimum (1A - C_{2v}) at CCSD(T)/haTZ

Irrep.	ω	IR
a_1	3723.4	205.7
a_1	1723.8	163.2
a_1	1104.7	893.9
a_1	598.3	1.4
a_1	339.7	55.4
a_1	189.9	1.0
a_2	438.0	0.0
b_1	3755.9	144.2
b_1	1035.1	678.2
b_1	353.0	70.4
b_1	323.4	28.1
b_1	110.3	8.5
b_2	704.8	225.9
b_2	516.4	327.8
b_2	35.7	4.1

TABLE S25. Harmonic vibrational frequencies (ω in cm^{-1}) along with their corresponding irreducible representations and infrared intensities (IR in km mol^{-1}) computed for the $BeF_3^-(H_2O)_2$ global minimum (2A - C_{2v}) using the haTZ basis set.

	CCSD(T):MP2	CCSD(T)	
Irrep.	ω	ω	IR
a_1	3794.2	3793.3	118.3
a_1	3736.3	3735.7	199.7
a_1	1725.3	1725.2	69.8
a_1	1041.4	1041.3	672.1
a_1	598.4	598.4	0.8
a_1	341.5	341.2	144.3
a_1	331.4	331.5	5.1
a_1	174.3	174.4	0.2
a_1	74.8	75.0	12.2
a_2	656.0	655.7	0.0
a_2	406.2	406.3	0.0
a_2	37.4	37.9	0.0
b_1	673.5	673.7	462.8
b_1	498.1	498.5	222.8
b_1	415.1	415.4	1.1
b_1	30.7	31.1	9.9
b_2	3787.1	3786.4	128.0
b_2	3733.7	3733.4	168.5
b_2	1710.8	1710.8	253.0
b_2	1100.3	1100.3	893.3
b_2	343.7	343.7	73.9
b_2	332.7	333.1	20.8
b_2	184.7	184.7	1.4
b_2	123.1	123.2	3.8

TABLE S26. Harmonic vibrational frequencies (ω in cm^{-1}) along with their corresponding irreducible representations and infrared intensities (IR in km mol^{-1}) computed for the $BeF_3^-(H_2O)_2$ local minimum (2B - C_1) using the haTZ basis set.

	CCSD(T):MP2	CCSD(T)	
Irrep.	ω	ω	IR
a	3871.7	3872.3	36.6
a	3771.5	3772.9	258.1
a	3654.8	3656.5	366.2
a	3489.2	3487.4	627.3
a	1726.2	1726.5	96.4
a	1702.2	1701.9	48.8
a	1120.3	1120.3	912.2
a	1028.0	1028.2	767.8
a	809.5	810.6	115.2
a	695.0	694.8	163.3
a	605.7	605.7	5.8
a	588.5	587.9	10.9
a	512.9	513.2	376.2
a	489.6	489.1	56.6
a	404.5	404.4	32.0
a	349.2	349.3	24.7
a	325.5	325.7	39.8
a	208.6	208.6	14.5
a	180.7	180.6	57.5
a	152.7	152.1	43.5
a	132.5	132.0	28.4
a	52.5	53.0	2.2
a	34.1	34.5	3.1
a	19.3	20.3	1.4

TABLE S27. Harmonic vibrational frequencies (ω in cm^{-1}) along with their corresponding irreducible representations and infrared intensities (IR in km mol^{-1}) computed for the $BeF_3^-(H_2O)_2$ local minimum (2C - C_1) using the haTZ basis set.

	CCSD(T):MP2	CCSD(T)	
Irrep.	ω	ω	IR
a	3829.5	3830.7	57.4
a	3796.0	3798.2	108.6
a	3691.5	3691.8	275.6
a	3610.6	3605.2	382.1
a	1723.8	1724.3	142.3
a	1705.9	1705.2	96.2
a	1124.4	1124.2	915.5
a	1020.1	1020.5	696.0
a	752.2	754.0	216.4
a	687.2	687.6	89.2
a	599.8	599.9	4.5
a	546.6	545.8	139.4
a	503.7	504.0	253.4
a	405.2	405.5	59.3
a	351.1	351.9	74.4
a	339.7	337.4	30.5
a	332.4	332.0	48.3
a	318.2	316.9	39.1
a	199.1	199.2	3.1
a	180.0	179.7	7.6
a	109.4	108.8	5.8
a	55.3	54.3	6.2
a	40.6	39.1	2.9
a	35.7	34.0	0.9

TABLE S28. Harmonic vibrational frequencies (ω in cm^{-1}) along with their corresponding irreducible representations and infrared intensities (IR in km mol^{-1}) computed for the $BeF_3^-(H_2O)_2$ local minimum (2D - C_{2v}) using the haTZ basis set.

	CCSD(T):MP2	CCSD(T)	
Irrep.	ω	ω	IR
a_1	3752.8	3752.0	102.3
a_1	1727.7	1727.5	108.7
a_1	1130.7	1130.4	317.3
a_1	668.0	667.2	244.4
a_1	591.7	591.7	2.2
a_1	339.0	338.6	55.3
a_1	171.5	171.4	0.1
a_1	52.0	51.6	5.6
a_2	3794.7	3794.8	0.0
a_2	379.6	380.1	0.0
a_2	308.4	308.3	0.0
a_2	76.7	76.8	0.0
b_1	3745.5	3745.1	147.5
b_1	1696.0	1695.5	214.0
b_1	607.6	606.9	202.3
b_1	462.3	462.2	6.1
b_1	172.6	172.3	7.7
b_1	42.5	42.4	15.3
b_2	3809.1	3808.6	205.4
b_2	1003.8	1003.9	221.7
b_2	440.1	439.2	2.1
b_2	348.7	349.0	138.8
b_2	313.2	313.1	2.1
b_2	106.9	106.9	0.1

TABLE S29. Harmonic vibrational frequencies (ω in cm^{-1}) along with their corresponding irreducible representations and infrared intensities (IR in km mol^{-1}) computed for the $BeF_3^-(H_2O)_2$ local minimum (2E - C_s) using the haTZ basis set.

	CCSD(T):MP2	CCSD(T)	
Irrep.	ω	ω	IR
a′	3770.6	3772.0	210.1
a′	3705.3	3705.2	17.7
a′	3691.5	3692.8	222.9
a′	1725.3	1725.4	102.3
a′	1712.7	1712.5	112.6
a′	1088.6	1088.7	322.5
a′	721.0	720.8	226.8
a′	604.8	604.8	6.8
a′	529.1	528.0	148.0
a′	479.2	479.5	71.4
a′	326.8	326.8	12.2
a′	196.0	195.8	11.4
a′	165.5	165.0	74.1
a′	114.6	113.2	38.5
a′	67.1	67.0	11.3
a″	3754.7	3753.5	143.4
a″	1054.8	1054.9	282.4
a″	656.1	655.3	44.0
a″	540.0	540.1	38.9
a″	468.9	467.8	56.1
a″	357.2	357.2	16.6
a″	308.7	308.7	5.4
a″	102.3	102.2	0.1
a″	47.7	47.6	15.5

TABLE S30. Harmonic vibrational frequencies (ω in cm^{-1}) along with their corresponding irreducible representations for the $BeF_3{}^-(H_2O)_3$ global minimum (3A - C_3) at CCSD(T):MP2/haTZ.

Irrep.	ω
e	3714.7
e	3714.7
e	3644.0
e	3644.0
e	1711.2
e	1711.2
e	1073.2
e	1073.2
e	712.1
e	712.1
e	556.5
e	556.5
e	489.6
e	489.6
e	314.2
e	314.2
e	195.0
e	195.0
e	138.8
e	138.8
e	59.9
e	59.9
a	3712.5
a	3618.0
a	1731.5
a	887.6
a	721.2
a	606.0
a	562.0
a	490.1
a	200.4
a	174.1
a	52.9

TABLE S31. Harmonic vibrational frequencies (ω in cm^{-1}) along with their corresponding irreducible representations for the $BeF_3{}^-(H_2O)_3$ local minimum (3B - C_1) at CCSD(T):MP2/haTZ.

Irrep.	ω
a	3777.8
a	3748.9
a	3741.4
a	3670.2
a	3657.5
a	3584.9
a	1730.7
a	1714.5
a	1708.5
a	1135.9
a	1013.4
a	852.9
a	776.0
a	692.1
a	661.4
a	602.5
a	532.3
a	515.3
a	509.7
a	464.5
a	428.2
a	377.0
a	340.1
a	325.2
a	203.1
a	191.5
a	173.8
a	142.8
a	121.9
a	108.9
a	67.3
a	56.1
a	32.5

TABLE S32. Harmonic vibrational frequencies (ω in cm^{-1}) along with their corresponding irreducible representations for the $BeF_3{}^-(H_2O)_3$ local minimum (3C - D_{3h}) at CCSD(T):MP2/haTZ.

Irrep.	ω
a_1'	3760.3
a_1'	1725.7
a_1'	598.4
a_1'	157.9
a_1''	381.8
a_2'	3802.5
a_2'	311.4
a_2'	129.9
a_2''	644.6
a_2''	478.1
a_2''	24.1
e'	3812.0
e'	3812.0
e'	3755.9
e'	3755.9
e'	1705.3
e'	1705.3
e'	1071.6
e'	1071.6
e'	343.4
e'	343.4
e'	322.0
e'	322.0
e'	173.6
e'	173.6
e'	70.6
e'	70.6
e''	612.5
e''	612.5
e''	393.6
e''	393.6
e''	36.4
e''	36.4

TABLE S33. Harmonic vibrational frequencies (ω in cm^{-1}) along with their corresponding irreducible representations for the $BeF_3^-(H_2O)_3$ local minimum (3D - C_1) at CCSD(T):MP2/haTZ.

Irrep.	ω
a	3874.5
a	3810.2
a	3773.6
a	3735.9
a	3694.1
a	3530.1
a	1723.3
a	1712.4
a	1698.7
a	1097.3
a	1051.9
a	786.3
a	659.4
a	641.6
a	606.0
a	574.1
a	495.2
a	478.1
a	395.2
a	389.3
a	354.1
a	330.9
a	327.1
a	213.3
a	176.1
a	168.0
a	153.9
a	136.1
a	90.5
a	47.0
a	40.7
a	27.6
a	18.0

TABLE S34. Harmonic vibrational frequencies (ω in cm^{-1}) along with their corresponding irreducible representations for the $BeF_3{}^-(H_2O)_3$ local minimum (3E - C_s) at CCSD(T):MP2/haTZ.

Irrep.	ω
a′	3775.7
a′	3729.5
a′	3676.1
a′	1721.9
a′	1713.0
a′	1030.2
a′	728.7
a′	675.6
a′	598.8
a′	489.5
a′	455.3
a′	339.5
a′	323.0
a′	206.6
a′	162.4
a′	106.7
a′	59.4
a′	42.6
a″	3768.9
a″	3762.2
a″	3711.4
a″	1702.9
a″	1112.1
a″	698.2
a″	647.2
a″	509.9
a″	424.8
a″	339.2
a″	326.6
a″	189.6
a″	132.5
a″	110.4
a″	45.7

TABLE S35. Harmonic vibrational frequencies (ω in cm^{-1}) along with their corresponding irreducible representations for the $BeF_3{}^-(H_2O)_3$ local minimum ($3F$ - C_1) at CCSD(T):MP2/haTZ.

Irrep.	ω
a	3874.0
a	3821.0
a	3775.0
a	3724.7
a	3683.9
a	3554.7
a	1723.8
a	1713.1
a	1697.1
a	1119.0
a	1028.8
a	767.1
a	670.4
a	649.8
a	603.8
a	574.6
a	496.5
a	475.1
a	386.6
a	376.6
a	355.5
a	327.7
a	323.1
a	200.5
a	187.4
a	165.8
a	150.3
a	133.0
a	91.2
a	45.4
a	36.6
a	32.1
a	15.3

TABLE S36. Harmonic vibrational frequencies (ω in cm^{-1}) along with their corresponding irreducible representations for the $BeF_3{}^-(H_2O)_3$ local minimum (3G - C_{2v}) at CCSD(T):MP2/haTZ.

Irrep.	ω
a_1	3735.7
a_1	3691.7
a_1	1726.6
a_1	1722.2
a_1	1143.4
a_1	729.9
a_1	595.1
a_1	343.8
a_1	202.1
a_1	177.5
a_1	73.0
a_2	3768.1
a_2	619.3
a_2	401.7
a_2	299.0
a_2	81.8
b_1	3785.6
b_1	994.9
b_1	744.4
b_1	470.2
b_1	350.6
b_1	314.9
b_1	117.5
b_1	47.6
b_2	3758.6
b_2	3711.9
b_2	1689.2
b_2	655.9
b_2	496.6
b_2	425.7
b_2	185.3
b_2	127.7
b_2	43.6

TABLE S37. Harmonic vibrational frequencies (ω in cm^{-1}) along with their corresponding irreducible representations for the $BeF_3^-(H_2O)_3$ local minimum (3H - C_1) at CCSD(T):MP2/haTZ.

Irrep.	ω
a	3806.9
a	3793.2
a	3785.3
a	3753.4
a	3716.7
a	3697.3
a	1722.7
a	1710.8
a	1703.8
a	1094.8
a	1048.0
a	701.5
a	651.1
a	624.2
a	597.6
a	508.6
a	486.1
a	461.3
a	446.7
a	390.4
a	342.5
a	333.1
a	319.2
a	314.2
a	190.2
a	171.6
a	158.0
a	111.3
a	106.1
a	83.9
a	63.4
a	44.2
a	29.9

TABLE S38. Harmonic vibrational frequencies (ω in cm^{-1}) along with their corresponding irreducible representations for the $BeF_3^-(H_2O)_3$ local minimum (3I - C_s) at CCSD(T):MP2/haTZ.

Irrep.	ω
a′	3780.9
a′	3750.6
a′	3730.8
a′	3707.2
a′	1724.2
a′	1719.0
a′	1691.3
a′	1112.6
a′	672.2
a′	631.2
a′	597.2
a′	494.4
a′	437.2
a′	338.2
a′	177.3
a′	172.1
a′	168.6
a′	123.5
a′	67.2
a′	34.6
a″	3805.8
a″	3792.2
a″	1024.6
a″	642.4
a″	531.9
a″	446.6
a″	414.1
a″	333.4
a″	307.2
a″	305.6
a″	108.4
a″	75.5
a″	37.4

TABLE S39. Harmonic vibrational frequencies (ω in cm^{-1}) along with their corresponding irreducible representations for the $BeF_3{}^-(H_2O)_3$ local minimum (3J - C_2) at CCSD(T):MP2/haTZ.

Irrep.	ω
b	3875.2
b	3738.2
b	3507.0
b	1703.5
b	1029.2
b	773.3
b	712.7
b	513.9
b	511.7
b	394.6
b	357.7
b	203.1
b	174.6
b	143.0
b	37.1
b	32.3
b	1.8i
a	3875.7
a	3670.8
a	3525.8
a	1720.6
a	1701.2
a	1123.4
a	788.6
a	655.9
a	611.6
a	403.2
a	319.0
a	233.6
a	197.0
a	162.2
a	56.8
a	12.7

TABLE S40. Harmonic vibrational frequencies (ω in cm^{-1}) along with their corresponding irreducible representations for the $BeF_3{}^-(H_2O)_3$ local minimum (3K - C_s) at CCSD(T):MP2/haTZ.

Irrep.	ω
a′	3878.6
a′	3671.0
a′	3524.6
a′	1722.1
a′	1703.9
a′	1122.1
a′	787.6
a′	701.5
a′	613.4
a′	512.1
a′	404.2
a′	317.4
a′	232.6
a′	189.4
a′	159.5
a′	61.6
a′	37.0
a′	17.9
a″	3878.0
a″	3740.1
a″	3504.3
a″	1699.8
a″	1030.3
a″	772.5
a″	650.5
a″	516.8
a″	387.4
a″	360.7
a″	200.3
a″	154.8
a″	141.1
a″	34.7
a″	19.7

IV. MP2 OPTIMIZED STRUCTURES

TABLE S41. Cartesian coordinates (in angstroms) for the BeF_3^- monomer at MP2/aTZ.

Be	0.0000000000	0.0000000000	0.0000000000
F	0.0000000000	0.0000000000	1.4851806114
F	1.2862041387	0.0000000000	-0.7425903057
F	-1.2862041387	0.0000000000	-0.7425903057

TABLE S42. Cartesian coordinates (in angstroms) for the $BeF_3^-(H_2O)_1$ global minimum (1A - C_{2v}) at MP2/haTZ.

Be	0.0000000000	0.0000000000	0.7877335216
F	1.2752052711	0.0000000000	0.0129886271
F	0.0000000000	0.0000000000	2.2586611348
F	-1.2752052711	0.0000000000	0.0129886271
H	0.7330184977	0.0000000000	-1.8656172427
H	-0.7330184977	0.0000000000	-1.8656172427
O	0.0000000000	0.0000000000	-2.5017180252

TABLE S43. Cartesian coordinates (in angstroms) for the $BeF_3^-(H_2O)_2$ global minimum (2A - C_{2v}) at MP2/haTZ.

Be	0.0000000000	0.0000000000	0.6323466625
F	0.0000000000	1.2956336412	1.3457995988
F	0.0000000000	-1.2956336412	1.3457995988
F	0.0000000000	0.0000000000	-0.8634295686
H	0.0000000000	2.6514115956	-0.0563357312
H	0.0000000000	1.9785287342	-1.3659903409
O	0.0000000000	2.8747720771	-1.0004919737
O	0.0000000000	-2.8747720771	-1.0004919737
H	0.0000000000	-2.6514115956	-0.0563357312
H	0.0000000000	-1.9785287342	-1.3659903409

TABLE S44. Cartesian coordinates (in angstroms) for the $BeF_3^-(H_2O)_2$ local minimum (2B - C_1) at MP2/haTZ.

Be	-1.3432292018	-0.0398565018	0.0014854405
F	-0.6175178940	-1.3227518794	-0.1784634471
F	-2.8060728955	0.0353035535	0.0310934788
F	-0.5169142937	1.2041926383	0.1388314027
O	2.1535159060	-1.5008853642	0.1385798529
O	2.1133304811	1.4958916616	-0.0406550636
H	1.1374448778	1.4231076205	0.0682872634
H	1.1869014711	-1.5473556783	0.0263854190
H	2.2985599513	-0.5477603710	0.2250302609
H	2.2282045976	1.4239453208	-0.9917846074

TABLE S45. Cartesian coordinates (in angstroms) for the $BeF_3^-(H_2O)_2$ local minimum (2D - C_{2v}) at MP2/haTZ.

Be	0.1515262689	0.0000000000	0.0000000000
F	1.6112282944	0.0000000000	0.0000000000
F	-0.6550245164	-1.2638194360	0.0000000000
F	-0.6550245164	1.2638194360	0.0000000000
O	-2.3778501793	0.0000000000	1.9453572926
H	-2.0068362929	0.7397512325	1.4431088122
H	-2.0068362929	-0.7397512325	1.4431088122
O	-2.3778501793	0.0000000000	-1.9453572926
H	-2.0068362929	0.7397512325	-1.4431088122
H	-2.0068362929	-0.7397512325	-1.4431088122

TABLE S46. Cartesian coordinates (in angstroms) for the $BeF_3^-(H_2O)_2$ local minimum (2E - C_s) at MP2/haTZ.

Be	0.6628868735	0.8060187014	0.0000000000
F	0.0248041490	1.2317358206	1.2735217760
F	0.0248041490	1.2317358206	-1.2735217760
F	1.8038561693	-0.1332528931	0.0000000000
O	-2.0426623890	-0.2109081011	0.0000000000
H	-1.6321478188	0.2583963288	0.7443670684
H	-1.6321478188	0.2583963288	-0.7443670684
O	0.0225900323	-2.4435718684	0.0000000000
H	0.7643431132	-1.8179604657	0.0000000000
H	-0.7485424598	-1.8563666720	0.0000000000

TABLE S47. Cartesian coordinates (in angstroms) for the $BeF_3^-(H_2O)_3$ global minimum (3A - C_3) at MP2/haTZ.

Be	-0.0000000131	0.0000000000	-1.3724366963
F	1.4767172645	-0.1261176478	-1.3219444637
F	-0.6291375651	1.3419335005	-1.3219444637
F	-0.8475797388	-1.2158158527	-1.3219444637
O	1.6915556325	-0.0510360193	1.4597338989
O	-0.8015793467	1.4904481707	1.4597338989
O	-0.8899763251	-1.4394121514	1.4597338989
H	1.8307526255	-0.0900772942	0.4962986031
H	-0.8373671073	1.6305169402	0.4962986031
H	-0.9933855575	-1.5404396459	0.4962986031
H	1.0506391035	0.6742228454	1.5539529721
H	-1.1092136834	0.5727687425	1.5539529721
H	0.0585745405	-1.2469915880	1.5539529721

TABLE S48. Cartesian coordinates (in angstroms) for the $BeF_3^-(H_2O)_3$ local minimum (3B - C_1) at MP2/haTZ.

F	-0.3327062114	-0.1022249294	2.8786102418
Be	0.2456193214	0.1333778664	1.5602205506
F	0.7127802038	1.4724630426	1.1108010163
F	0.4079427809	-0.9858537873	0.5686647736
O	-0.1551522673	-0.3973344285	-2.1924489993
H	0.3580626817	0.4239934731	-2.0889892533
H	-0.2318990372	-0.6874866581	-1.2697129728
O	1.8151262533	1.5589268255	-1.3792632823
H	1.4383983250	1.6407366808	-0.4799776632
H	2.3370302375	0.7448305404	-1.3146364739
O	2.4922090674	-1.3588360829	-1.2415963076
H	1.9299019121	-1.3404173934	-0.4467919153
H	1.8137387329	-1.3214381493	-1.9311397145

TABLE S49. Cartesian coordinates (in angstroms) for the $BeF_3^-(H_2O)_3$ local minimum (3C - D_{3h}) at MP2/haTZ.

Be	0.0000000000	0.0000002787	0.0000000000
F	0.0000000937	1.4842633375	0.0000000000
F	-1.2854095617	-0.7421311696	0.0000000000
F	1.2854094680	-0.7421313319	0.0000000000
H	1.9670497030	1.9886234385	0.0000000000
H	2.7057231160	0.7092033705	0.0000000000
O	2.8778848933	1.6615476543	0.0000000000
H	-2.7057230264	0.7092037121	0.0000000000
H	-1.9670494519	1.9886236869	0.0000000000
H	0.7386733234	-2.6978263145	0.0000000000
H	-0.7386736641	-2.6978262213	0.0000000000
O	-0.0000002098	-3.3230948358	0.0000000000
O	-2.8778846835	1.6615480176	0.0000000000

TABLE S50. Cartesian coordinates (in angstroms) for the $BeF_3^-(H_2O)_3$ local minimum (3D - C_1) at MP2/haTZ.

O	-2.3892926809	-0.5727072982	-1.2618277576
F	1.2057544075	2.1192404306	1.2831862699
Be	0.4846764427	0.9846159848	0.6784252882
F	0.9632070677	-0.4046817886	0.9126268778
F	-0.7287189696	1.1971146982	-0.1533674592
O	-0.3197204044	-2.5632424646	-0.4294770253
H	-2.9748197042	-0.7567188548	-0.5227831749
H	-1.7857340738	0.1136592356	-0.9022927086
H	0.1935002555	-1.9026640434	0.0642236415
H	-1.0022735244	-2.0202228620	-0.8503087551
O	3.1137066003	0.5422687518	2.6520865878
H	2.6177256207	-0.1578024430	2.2069542648
H	2.6264329629	1.3145316535	2.3241959507

TABLE S51. Cartesian coordinates (in angstroms) for the $BeF_3^-(H_2O)_3$ local minimum (3E - C_s) at MP2/haTZ.

Be	0.0111138798	-1.3663418087	0.0000000000
F	0.6480372677	-1.6566140188	1.2993133565
F	0.6480372677	-1.6566140188	-1.2993133565
F	-1.3127716558	-0.6562860982	0.0000000000
O	-0.3044585812	0.8719181293	2.1421171518
H	0.2527513899	0.0784799833	2.1832706387
H	-0.9833059350	0.5594613188	1.5241039080
O	-0.3044585812	0.8719181293	-2.1421171518
H	0.2527513899	0.0784799833	-2.1832706387
H	-0.9833059350	0.5594613188	-1.5241039080
O	0.6926105952	2.7074925421	0.0000000000
H	0.4496843491	2.1580455697	-0.7620789182
H	0.4496843491	2.1580455697	0.7620789182

TABLE S52. Cartesian coordinates (in angstroms) for the $BeF_3^-(H_2O)_3$ local minimum (3F - C_1) at MP2/haTZ.

O	-3.2284885164	-0.5991260077	-0.0883462608
F	1.8484115021	-1.3536427301	0.0804040426
Be	0.5503810069	-0.6535218355	0.0531787074
F	0.4277112756	0.8091566398	-0.0590193298
F	-0.7002768194	-1.4833347993	0.1274951035
O	-2.0498570554	2.1477779402	0.1234456678
H	-3.3163670120	-0.5537187852	-1.0440084725
H	-2.3217628585	-0.9521319744	0.0344829661
H	-1.1446375981	1.7982658476	0.0615365152
H	-2.5756320526	1.3362920292	0.1702084546
O	0.8571888806	-3.9828716256	0.1791933129
H	1.4586560556	-3.2210642911	0.1458721413
H	0.0118707915	-3.5165370079	0.1966719518

TABLE S53. Cartesian coordinates (in angstroms) for the $BeF_3^-(H_2O)_3$ local minimum (3G - C_{2v}) at MP2/haTZ.

O	0.0000000000	1.7778094017	-1.0730254537
F	-1.2578395195	0.0000000000	0.7802180998
Be	0.0000000000	0.0000000000	1.5985745927
F	0.0000000000	0.0000000000	3.0536627403
F	1.2578395195	0.0000000000	0.7802180998
O	0.0000000000	-1.7778094017	-1.0730254537
H	0.7418020479	1.3513661623	-0.6166909057
H	-0.7418020479	1.3513661623	-0.6166909057
H	0.7418020479	-1.3513661623	-0.6166909057
H	-0.7418020479	-1.3513661623	-0.6166909057
O	0.0000000000	0.0000000000	-3.4219064271
H	0.0000000000	0.7481714678	-2.8035572375
H	0.0000000000	-0.7481714678	-2.8035572375

TABLE S54. Cartesian coordinates (in angstroms) for the $BeF_3^-(H_2O)_3$ local minimum (3H - C_1) at MP2/haTZ.

O	-2.1607597458	-0.2517540790	-0.1536123786
F	-0.0420737986	1.1963855700	1.2204580980
Be	0.5697049335	0.7449365127	-0.0624555855
F	1.7070080000	-0.2058396235	-0.0174376589
F	-0.0571572382	1.1552511113	-1.3357289764
O	-0.1672050007	-2.5151116980	-0.2673979553
H	-1.8275387405	0.1878452989	0.6417789028
H	-1.7003054399	0.2511523675	-0.8461401002
H	0.5999221703	-1.9280122175	-0.2051664287
H	-0.9144835089	-1.8985924653	-0.2310060816
O	2.2304321414	0.3954942723	2.7667817215
H	2.4006463795	-0.0281536378	1.9137477889
H	1.3662538478	0.7897895884	2.5778206540

TABLE S55. Cartesian coordinates (in angstroms) for the $BeF_3^-(H_2O)_3$ local minimum (3I - C_s) at MP2/haTZ.

Be	0.1099940992	1.7856437728	-1.1606211373
F	-0.6609447007	1.2889351423	-0.0146150921
F	1.5681102330	2.0926838960	-1.0602464887
F	-0.5364400376	2.1167199276	-2.4655973262
O	0.2654220594	4.6492310889	-1.3444020648
H	-0.1996801649	4.1155275963	-2.0056590671
H	1.0458874316	4.1013019907	-1.1739091729
O	1.9411533657	1.9423442627	-3.9001507758
H	0.9807334809	1.9967053325	-3.7905343024
H	2.2086936976	1.9826808205	-2.9705420489
O	-1.3634510916	3.8797571173	1.0817183308
H	-1.2441858612	2.9371050020	0.8869930838
H	-0.8755345521	4.3001936212	0.3582411513

TABLE S56. Cartesian coordinates (in angstroms) for the $BeF_3^-(H_2O)_3$ local minimum (3J - C_2) at MP2/haTZ.

Be	0.0000000000	0.0000000000	-1.8643816441
F	0.0000000000	0.0000000000	-3.3261121262
F	-0.2820228681	1.2430688470	-1.0885426503
F	0.2820228681	-1.2430688470	-1.0885426503
O	0.0233587981	2.2095710969	1.3704044253
O	-0.0233587981	-2.2095710969	1.3704044253
H	0.1381470770	-1.8448724530	0.4719157378
H	-0.9726952425	-2.3559660056	1.3787547868
H	-0.1381470770	1.8448724530	0.4719157378
H	0.9726952425	2.3559660056	1.3787547868
O	0.0000000000	0.0000000000	3.2864023108
H	-0.0817174780	0.7549978835	2.6806008301
H	0.0817174780	-0.7549978835	2.6806008301

TABLE S57. Cartesian coordinates (in angstroms) for the $BeF_3^-(H_2O)_3$ local minimum (3K - C_s) at MP2/haTZ.

Be	0.2814120816	-1.9724560881	0.0000000000
F	0.3522916908	-3.4326580276	0.0000000000
F	0.2316484061	-1.1994880086	-1.2752868387
F	0.2316484061	-1.1994880086	1.2752868387
O	-0.0385662777	1.2677569129	-2.2239098449
O	-0.0385662777	1.2677569129	2.2239098449
H	0.1157033206	0.3658249976	1.8642925789
H	-0.9849203430	1.2788991921	2.3869080138
H	0.1157033206	0.3658249976	-1.8642925789
H	-0.9849203430	1.2788991921	-2.3869080138
O	0.1200047090	3.1656363812	0.0000000000
H	0.1462618533	2.5614516233	-0.7601486056
H	0.1462618533	2.5614516233	0.7601486056

V. MP2 VIBRATIONAL FREQUENCIES

TABLE S58. Harmonic vibrational frequencies (ω in cm^{-1}) along with their corresponding irreducible representations and infrared intensities (IR in km mol^{-1}) computed for the $BeF_3{}^-$ monomer at MP2/haTZ.

Irrep.	ω	IR
A1′	591.2	0.0
A2″	531.1	119.8
E′	1054.4	360.0
E′	1054.4	360.0
E′	319.2	8.8
E′	319.2	8.8

TABLE S59. Harmonic vibrational frequencies (ω in cm^{-1}) along with their corresponding irreducible representations and infrared intensities (IR in km mol^{-1}) computed for the $BeF_3{}^-(H_2O)_1$ global minimum (1A - C_{2v}) at MP2/haTZ.

Irrep.	ω	IR
A_1	3715.1	228.6
A_1	1705.2	175.3
A_1	1090.5	430.0
A_1	590.8	0.7
A_1	336.4	24.7
A_1	186.1	9.8
A_2	443.5	0.0
B_1	707.8	183.3
B_1	514.5	73.6
B_1	36.5	1.1
B_2	3759.3	163.9
B_2	1020.9	284.5
B_2	354.0	71.1
B_2	320.2	6.0
B_2	104.2	2.3

TABLE S60. Harmonic vibrational frequencies (ω in cm^{-1}) along with their corresponding irreducible representations and infrared intensities (IR in km mol^{-1}) computed for the $BeF_3{}^-(H_2O)_2$ global minimum (2A - C_{2v}) at MP2/haTZ.

Irrep.	ω	IR
A_1	3801.6	141.5
A_1	3728.7	222.0
A_1	1706.7	73.6
A_1	1028.2	280.6
A_1	591.2	0.3
A_1	341.6	127.9
A_1	327.5	0.0
A_1	170.4	1.8
A_1	70.8	2.6
A_2	659.0	0.0
A_2	410.3	0.0
A_2	38.5	0.0
B_1	676.3	366.6
B_1	497.0	32.0
B_1	419.6	2.3
B_1	31.4	2.3
B_2	3794.2	139.6
B_2	3726.1	191.5
B_2	1692.2	271.4
B_2	1086.0	429.6
B_2	339.9	43.2
B_2	333.5	19.7
B_2	180.7	12.1
B_2	114.7	0.9

TABLE S61. Harmonic vibrational frequencies (ω in cm^{-1}) along with their corresponding irreducible representations and infrared intensities (IR in km mol^{-1}) computed for the $BeF_3{}^-(H_2O)_2$ local minimum (2B - C_1) at MP2/haTZ.

Irrep.	ω	IR
A	3899.6	48.8
A	3770.1	311.4
A	3643.6	382.4
A	3450.8	686.7
A	1709.2	95.7
A	1684.9	50.5
A	1106.4	442.3
A	1013.7	347.5
A	819.6	121.2
A	696.8	145.1
A	598.8	3.0
A	595.1	12.7
A	511.4	95.6
A	496.6	53.8
A	409.0	37.3
A	346.9	7.5
A	322.8	15.7
A	207.5	26.3
A	177.4	62.7
A	152.6	42.1
A	129.2	22.5
A	55.7	0.2
A	35.4	1.6
A	22.7	1.8

TABLE S62. Harmonic vibrational frequencies (ω in cm^{-1}) along with their corresponding irreducible representations and infrared intensities (IR in km mol^{-1}) computed for the $BeF_3{}^-(H_2O)_2$ local minimum (2D - C_{2v}) at MP2/haTZ.

Irrep.	ω	IR
A_1	3749.9	113.7
A_1	1709.1	105.4
A_1	1117.1	430.6
A_1	668.7	226.5
A_1	584.9	4.6
A_1	335.9	16.9
A_1	166.9	3.3
A_1	52.3	0.7
A_2	3804.2	0.0
A_2	383.0	0.0
A_2	308.5	0.0
A_2	67.1	0.0
B_1	3742.6	168.1
B_1	1677.5	216.8
B_1	608.6	219.7
B_1	462.3	17.1
B_1	167.0	7.4
B_1	42.7	0.7
B_2	3819.2	242.8
B_2	990.1	232.0
B_2	441.9	0.9
B_2	348.0	146.1
B_2	310.3	5.9
B_2	100.4	5.3

TABLE S63. Harmonic vibrational frequencies (ω in cm^{-1}) along with their corresponding irreducible representations and infrared intensities (IR in km mol^{-1}) computed for the $BeF_3{}^-(H_2O)_2$ local minimum (2E - C_s) at MP2/haTZ.

Irrep.	ω	IR
A′	3776.5	310.5
A′	3699.4	22.5
A′	3684.3	283.9
A′	1707.4	140.3
A′	1694.3	112.5
A′	1075.3	322.2
A′	722.7	237.0
A′	597.8	7.2
A′	528.9	154.1
A′	478.4	71.7
A′	324.3	11.8
A′	191.5	9.8
A′	160.3	7.9
A′	108.4	1.1
A′	67.8	0.4
A″	3759.0	164.7
A″	1040.8	287.6
A″	662.1	58.7
A″	541.7	56.3
A″	471.1	64.6
A″	359.8	14.2
A″	306.4	4.6
A″	94.6	0.0
A″	48.0	0.4

TABLE S64. Harmonic vibrational frequencies (ω in cm^{-1}) along with their corresponding irreducible representations and infrared intensities (IR in km mol^{-1}) computed for the $BeF_3^-(H_2O)_3$ global minimum (3A - C_3) at MP2/haTZ.

Irrep.	ω	IR
E	3712.4	336.2
E	3712.4	336.2
E	3631.1	40.5
E	3631.1	40.5
E	1693.9	45.6
E	1693.9	45.6
E	1059.2	299.1
E	1059.2	299.1
E	715.4	260.4
E	715.4	260.4
E	562.5	121.3
E	562.5	121.3
E	494.4	9.1
E	494.4	9.1
E	311.8	6.7
E	311.8	6.7
E	190.4	8.3
E	190.4	8.3
E	133.2	8.7
E	133.2	8.7
E	60.8	0.1
E	60.8	0.1
A	3709.3	695.6
A	3602.0	267.5
A	1714.7	128.0
A	897.1	2.9
A	725.4	6.2
A	599.2	3.6
A	565.6	147.2
A	488.4	100.2
A	196.2	17.4
A	168.4	3.1
A	54.2	0.3

TABLE S65. Harmonic vibrational frequencies (ω in cm^{-1}) along with their corresponding irreducible representations and infrared intensities (IR in km mol^{-1}) computed for the $BeF_3^-(H_2O)_3$ local minimum (3B - C_1) at MP2/haTZ.

Irrep.	ω	IR
A	3786.9	216.4
A	3751.9	305.9
A	3746.2	268.0
A	3660.4	194.6
A	3641.7	196.2
A	3561.4	401.7
A	1713.5	91.8
A	1697.2	67.3
A	1691.0	110.8
A	1121.5	450.7
A	999.9	328.1
A	862.1	20.8
A	783.2	154.6
A	696.3	270.2
A	658.2	112.4
A	595.3	15.5
A	537.9	22.9
A	517.7	122.2
A	508.6	77.0
A	467.9	58.4
A	430.2	14.4
A	377.4	27.2
A	336.8	16.4
A	322.5	16.3
A	198.9	20.3
A	188.8	12.3
A	169.7	11.1
A	136.7	9.4
A	114.2	1.6
A	100.9	2.4
A	69.4	0.3
A	57.6	0.5
A	33.3	1.3

TABLE S66. Harmonic vibrational frequencies (ω in cm^{-1}) along with their corresponding irreducible representations and infrared intensities (IR in km mol^{-1}) computed for the $BeF_3{}^-(H_2O)_3$ local minimum (3C - D_{3h}) at MP2/haTZ.

Irrep.	ω	IR
A_1'	3756.1	0.0
A_1'	1707.1	0.0
A_1'	591.5	0.0
A_1'	153.8	0.0
A_1''	385.6	0.0
A_2'	3810.7	0.0
A_2'	312.0	0.0
A_2'	119.3	0.0
A_2''	646.6	546.6
A_2''	477.8	3.4
A_2''	24.6	3.6
E'	3820.7	197.2
E'	3820.7	197.2
E'	3751.7	264.7
E'	3751.7	264.7
E'	1686.6	257.5
E'	1686.6	257.5
E'	1058.3	351.0
E'	1058.3	351.0
E'	339.9	67.9
E'	339.9	67.9
E'	320.0	74.4
E'	320.0	74.4
E'	169.1	5.0
E'	169.1	5.0
E'	66.8	2.8
E'	66.8	2.8
E''	614.6	0.0
E''	614.6	0.0
E''	397.6	0.0
E''	397.6	0.0
E''	37.5	0.0
E''	37.5	0.0

TABLE S67. Harmonic vibrational frequencies (ω in cm^{-1}) along with their corresponding irreducible representations and infrared intensities (IR in km mol^{-1}) computed for the $BeF_3^-(H_2O)_3$ local minimum (3D - C_1) at MP2/haTZ.

Irrep.	ω	IR
A	3901.7	55.3
A	3820.9	104.2
A	3773.2	401.2
A	3726.6	216.8
A	3685.0	258.5
A	3496.0	640.7
A	1706.3	104.4
A	1694.1	140.7
A	1681.2	60.7
A	1082.6	432.8
A	1039.3	347.4
A	795.0	133.8
A	663.1	312.7
A	645.5	35.1
A	598.9	2.3
A	579.4	4.7
A	494.6	43.7
A	483.4	51.1
A	397.6	3.8
A	394.2	37.5
A	351.5	18.0
A	330.8	73.7
A	324.3	16.5
A	211.6	42.5
A	173.3	63.6
A	165.2	8.8
A	154.2	22.9
A	130.8	14.9
A	86.5	0.8
A	48.1	1.3
A	42.7	0.7
A	28.4	1.7
A	19.2	1.1

TABLE S68. Harmonic vibrational frequencies (ω in cm^{-1}) along with their corresponding irreducible representations and infrared intensities (IR in km mol^{-1}) computed for the $BeF_3^-(H_2O)_3$ local minimum (3E - C_s) at MP2/haTZ.

Irrep.	ω	IR
A′	3780.3	212.1
A′	3722.7	178.3
A′	3664.4	326.3
A′	1704.5	91.2
A′	1695.8	206.1
A′	1016.6	265.1
A′	729.6	205.8
A′	683.4	67.0
A′	591.9	6.4
A′	488.2	100.3
A′	457.7	61.1
A′	340.2	52.5
A′	319.9	17.1
A′	202.1	8.4
A′	159.2	7.0
A′	100.0	2.0
A′	61.1	0.5
A′	44.0	1.6
A″	3771.8	287.9
A″	3763.4	240.0
A″	3704.0	1.1
A″	1684.5	80.3
A″	1098.7	353.2
A″	698.7	332.7
A″	654.0	27.4
A″	514.6	2.0
A″	427.1	0.0
A″	337.2	37.0
A″	326.4	0.4
A″	184.9	11.6
A″	126.8	2.8
A″	101.7	0.2
A″	45.7	0.4

TABLE S69. Harmonic vibrational frequencies (ω in cm^{-1}) along with their corresponding irreducible representations and infrared intensities (IR in km mol^{-1}) computed for the $BeF_3^-(H_2O)_3$ local minimum (3F - C_1) at MP2/haTZ.

Irrep.	ω	IR
A	3901.1	57.7
A	3834.4	102.2
A	3774.7	339.7
A	3711.6	218.0
A	3674.1	312.1
A	3522.3	623.4
A	1707.0	48.6
A	1694.6	200.9
A	1679.7	64.8
A	1105.4	457.5
A	1015.0	319.4
A	777.2	122.5
A	672.4	240.7
A	654.5	95.1
A	596.8	2.0
A	580.9	6.7
A	495.9	49.8
A	481.7	50.6
A	389.4	39.0
A	378.8	7.2
A	352.5	19.3
A	328.0	95.5
A	320.7	1.7
A	198.7	19.2
A	183.8	33.9
A	162.7	32.7
A	151.1	38.4
A	128.7	13.9
A	86.4	2.1
A	46.6	0.8
A	38.8	1.9
A	33.1	0.2
A	17.1	3.2

TABLE S70. Harmonic vibrational frequencies (ω in cm^{-1}) along with their corresponding irreducible representations and infrared intensities (IR in km mol^{-1}) computed for the $BeF_3^-(H_2O)_3$ local minimum (3G - C_{2v}) at MP2/haTZ.

Irrep.	ω	IR
A_1	3730.1	100.0
A_1	3679.3	372.2
A_1	1707.9	126.6
A_1	1705.2	150.6
A_1	1129.8	475.6
A_1	731.0	184.3
A_1	588.1	0.1
A_1	340.5	20.4
A_1	197.0	0.8
A_1	174.2	15.6
A_1	73.2	0.2
A_2	3773.7	0.0
A_2	628.0	0.0
A_2	406.1	0.0
A_2	298.2	0.0
A_2	71.7	0.0
B_1	3791.9	269.5
B_1	981.7	223.5
B_1	750.8	114.9
B_1	471.7	45.9
B_1	349.6	105.7
B_1	312.0	4.5
B_1	110.9	1.1
B_1	47.9	4.3
B_2	3757.8	382.8
B_2	3705.5	8.0
B_2	1670.7	161.9
B_2	658.1	337.1
B_2	497.0	13.2
B_2	430.9	21.5
B_2	179.8	13.9
B_2	121.9	0.6
B_2	43.4	1.8

TABLE S71. Harmonic vibrational frequencies (ω in cm^{-1}) along with their corresponding irreducible representations and infrared intensities (IR in km mol^{-1}) computed for the $BeF_3{}^{-}(H_2O)_3$ local minimum (3H - C_1) at MP2/haTZ.

Irrep.	ω	IR
A	3815.7	197.8
A	3803.1	201.0
A	3795.2	93.4
A	3748.2	184.9
A	3709.6	117.3
A	3685.8	214.9
A	1704.4	118.8
A	1692.6	54.3
A	1685.2	234.3
A	1080.7	343.6
A	1036.0	262.4
A	707.3	200.2
A	655.2	196.4
A	626.2	113.3
A	591.9	6.5
A	508.1	132.8
A	485.7	67.4
A	462.8	38.7
A	445.1	65.6
A	393.9	1.9
A	345.1	15.9
A	330.7	36.8
A	319.0	49.5
A	311.5	2.2
A	186.7	8.2
A	167.1	4.5
A	153.2	5.5
A	104.0	0.9
A	97.6	1.9
A	77.1	0.8
A	63.5	1.3
A	44.4	0.4
A	30.5	1.1

TABLE S72. Harmonic vibrational frequencies (ω in cm^{-1}) along with their corresponding irreducible representations and infrared intensities (IR in km mol^{-1}) computed for the $BeF_3^-(H_2O)_3$ local minimum (3I - C_s) at MP2/haTZ.

Irrep.	ω	IR
A′	3786.7	278.3
A′	3746.4	136.1
A′	3727.8	44.0
A′	3701.2	257.0
A′	1706.1	49.0
A′	1700.7	133.4
A′	1672.5	251.8
A′	1099.4	376.7
A′	672.8	284.5
A′	632.3	164.9
A′	590.6	3.2
A′	495.0	107.8
A′	437.1	53.5
A′	335.1	18.5
A′	172.3	14.2
A′	166.7	2.8
A′	164.2	2.4
A′	118.1	0.5
A′	67.8	0.1
A′	35.3	1.8
A″	3815.1	250.2
A″	3800.8	0.2
A″	1011.3	236.5
A″	647.7	89.5
A″	534.8	32.5
A″	447.4	53.0
A″	417.2	12.5
A″	334.0	68.8
A″	307.6	16.0
A″	302.7	0.1
A″	102.1	2.5
A″	64.1	0.2
A″	37.6	2.1

TABLE S73. Harmonic vibrational frequencies (ω in cm^{-1}) along with their corresponding irreducible representations and infrared intensities (IR in km mol^{-1}) computed for the $BeF_3^-(H_2O)_3$ local minimum (3J - C_2) at MP2/haTZ.

Irrep.	ω	IR
B	3902.0	71.0
B	3731.1	561.3
B	3471.1	194.1
B	1686.2	44.6
B	1015.6	398.1
B	781.2	169.0
B	719.2	172.4
B	525.3	33.4
B	510.7	109.8
B	398.6	22.9
B	356.9	1.1
B	200.6	63.9
B	174.0	134.7
B	140.2	17.4
B	38.1	3.3
B	33.9	2.4
B	9.2	5.6
A	3902.5	39.2
A	3651.9	159.8
A	3490.9	1342.7
A	1704.3	50.9
A	1683.5	91.9
A	1109.1	463.7
A	795.9	20.1
A	666.6	2.6
A	604.9	14.2
A	407.9	53.3
A	316.0	12.2
A	231.9	7.8
A	193.6	40.8
A	160.1	2.3
A	59.5	0.2
A	19.1	0.7

TABLE S74. Harmonic vibrational frequencies (ω in cm^{-1}) along with their corresponding irreducible representations and infrared intensities (IR in km mol^{-1}) computed for the $BeF_3{}^-(H_2O)_3$ local minimum (3K - C_s) at MP2/haTZ.

Irrep.	ω	IR
A′	3905.6	95.3
A′	3652.9	160.4
A′	3489.6	1404.1
A′	1705.8	36.7
A′	1686.1	142.4
A′	1108.3	462.7
A′	794.6	21.5
A′	707.8	165.8
A′	606.1	15.9
A′	510.3	110.2
A′	408.8	70.1
A′	314.9	15.2
A′	231.4	11.0
A′	187.9	46.4
A′	158.8	2.9
A′	62.8	0.3
A′	37.7	0.6
A′	18.2	3.6
A″	3905.0	13.5
A″	3733.3	564.0
A″	3468.6	151.2
A″	1682.3	4.5
A″	1016.1	404.7
A″	780.1	178.3
A″	661.2	6.5
A″	527.0	31.5
A″	392.2	3.6
A″	358.7	0.2
A″	198.7	51.9
A″	155.4	113.4
A″	138.9	35.5
A″	35.6	0.1
A″	21.7	3.1

VI. B3LYP-D3BJ OPTIMIZED STRUCTURES

TABLE S75. Cartesian coordinates (in angstroms) for the BeF_3^- monomer at B3LYP-D3BJ/aTZ.

Be	0.0000000000	0.0000000000	0.0000000000
F	0.0000000000	0.0000000000	1.4780952786
F	1.2800680605	0.0000000000	-0.7390476393
F	-1.2800680605	0.0000000000	-0.7390476393

TABLE S76. Cartesian coordinates (in angstroms) for the $BeF_3^-(H_2O)_1$ global minimum (1A - C_{2v}) at B3LYP-D3BJ/haTZ.

Be	0.0000000000	0.0000000000	0.7858516342
F	1.2704442017	0.0000000000	0.0168133209
F	0.0000000000	0.0000000000	2.2495737164
F	-1.2704442017	0.0000000000	0.0168133209
H	0.7381252868	0.0000000000	-1.8661990362
H	-0.7381252868	0.0000000000	-1.8661990362
O	0.0000000000	0.0000000000	-2.4972345200

TABLE S77. Cartesian coordinates (in angstroms) for the $BeF_3^-(H_2O)_2$ global minimum (2A - C_{2v}) at B3LYP-D3BJ/haTZ.

Be	0.0000000000	0.0000000000	0.6288999118
F	0.0000000000	1.2881164360	1.3413769771
F	0.0000000000	-1.2881164360	1.3413769771
F	0.0000000000	0.0000000000	-0.8596518548
H	0.0000000000	2.6528650857	-0.0509313356
H	0.0000000000	1.9773158960	-1.3706048422
O	0.0000000000	2.8706232039	-0.9970247277
O	0.0000000000	-2.8706232039	-0.9970247277
H	0.0000000000	-2.6528650857	-0.0509313356
H	0.0000000000	-1.9773158960	-1.3706048422

TABLE S78. Cartesian coordinates (in angstroms) for the $BeF_3{}^{-}(H_2O)_2$ local minimum (2B - C_1) at B3LYP-D3BJ/haTZ.

Be	-1.3335735183	-0.0308969294	-0.0010196737
F	-0.6005354622	-1.2943958871	-0.2242451846
F	-2.7895364912	0.0255903933	0.0400364379
F	-0.5267261714	1.2133298327	0.1677475773
O	2.1543086777	-1.4896801877	0.1482748796
O	2.0995148309	1.4915984714	-0.0541705520
H	1.1243837280	1.4248310704	0.0768716393
H	1.1897173789	-1.5394266297	0.0149534337
H	2.3054859708	-0.5382322387	0.2498827005
H	2.2111840570	1.3611131047	-0.9995412580

TABLE S79. Cartesian coordinates (in angstroms) for the $BeF_3{}^{-}(H_2O)_2$ local minimum (2D - C_{2v}) at B3LYP-D3BJ/haTZ.

Be	0.1508481151	0.0000000000	0.0000000000
F	1.6033799980	0.0000000000	0.0000000000
F	-0.6484472482	-1.2601389460	0.0000000000
F	-0.6484472482	1.2601389460	0.0000000000
O	-2.3778752175	0.0000000000	1.9405185550
H	-2.0079807954	0.7446257652	1.4440117759
H	-2.0079807954	-0.7446257652	1.4440117759
O	-2.3778752175	0.0000000000	-1.9405185550
H	-2.0079807954	0.7446257652	-1.4440117759
H	-2.0079807954	-0.7446257652	-1.4440117759

TABLE S80. Cartesian coordinates (in angstroms) for the $BeF_3{}^{-}(H_2O)_2$ local minimum (2E - C_s) at B3LYP-D3BJ/haTZ.

Be	0.6674833920	0.8041616978	0.0000000000
F	0.0311918111	1.2243669478	1.2681931307
F	0.0311918111	1.2243669478	-1.2681931307
F	1.8085625389	-0.1238291963	0.0000000000
O	-2.0465235998	-0.2123823288	0.0000000000
H	-1.6322950755	0.2470467968	0.7488699660
H	-1.6322950755	0.2470467968	-0.7488699660
O	0.0167939857	-2.4293528096	0.0000000000
H	0.7635891760	-1.8092064979	0.0000000000
H	-0.7599149643	-1.8479953544	0.0000000000

TABLE S81. Cartesian coordinates (in angstroms) for the $BeF_3^-(H_2O)_3$ global minimum (3A - C_3) at B3LYP-D3BJ/haTZ.

Be	-0.0005090815	-0.0002248537	-1.3694960438
F	1.4684697365	-0.1344704499	-1.3222977716
F	-0.6187192120	1.3390642998	-1.3219454323
F	-0.8512372902	-1.2052584413	-1.3215047271
O	1.6872943567	-0.0503231395	1.4566953022
O	-0.7994994285	1.4863506716	1.4571741603
O	-0.8868918148	-1.4356177421	1.4576123720
H	1.8262904652	-0.0994977124	0.4932486938
H	-0.8269268247	1.6313693094	0.4937524528
H	-0.9992723292	-1.5318388033	0.4942440260
H	1.0457020635	0.6747164084	1.5576956460
H	-1.1065397082	0.5681849785	1.5582786033
H	0.0618388967	-1.2424545253	1.5582290582

TABLE S82. Cartesian coordinates (in angstroms) for the $BeF_3^-(H_2O)_3$ local minimum (3B - C_1) at B3LYP-D3BJ/haTZ.

F	-0.3306203468	-0.0967251947	2.8655562221
Be	0.2464386280	0.1249990578	1.5521208505
F	0.7146673627	1.4546252066	1.0993408078
F	0.4030557450	-0.9990698521	0.5773986446
O	-0.1542382057	-0.3901730929	-2.1848611515
H	0.3617593374	0.4313539063	-2.0849527132
H	-0.2405125073	-0.6888083559	-1.2656575333
O	1.8178680207	1.5586406787	-1.3821221405
H	1.4360468380	1.6420577394	-0.4841844353
H	2.3433718663	0.7454900206	-1.3254884520
O	2.4878454826	-1.3515303155	-1.2370303677
H	1.9393013770	-1.3384628232	-0.4321979952
H	1.8060684021	-1.3116599749	-1.9241817360

TABLE S83. Cartesian coordinates (in angstroms) for the $BeF_3^-(H_2O)_3$ local minimum (3C - D_{3h}) at B3LYP-D3BJ/haTZ.

Be	0.0000000000	0.0000000000	0.0000000000
F	0.0000000043	1.4771529210	0.0000000000
F	1.2792519527	-0.7385764642	0.0000000000
F	-1.2792519570	-0.7385764567	0.0000000000
H	-1.9627477910	1.9919374721	0.0000000000
H	-2.7064423449	0.7038207281	0.0000000000
O	-2.8714946400	1.6578582147	0.0000000000
H	2.7064423490	0.7038207122	0.0000000000
H	1.9627478027	1.9919374605	0.0000000000
H	-0.7436945581	-2.6957581842	0.0000000000
H	0.7436945422	-2.6957581886	0.0000000000
O	-0.0000000097	-3.3157164124	0.0000000000
O	2.8714946498	1.6578581978	0.0000000000

TABLE S84. Cartesian coordinates (in angstroms) for the $BeF_3^-(H_2O)_3$ local minimum (3D - C_1) at B3LYP-D3BJ/haTZ.

O	-2.3854366644	-0.5694053261	-1.2309828657
F	1.2157203952	2.1140713693	1.2558253541
Be	0.4830745195	0.9899915386	0.6628847649
F	0.9342043654	-0.3977043287	0.9126146432
F	-0.7166245259	1.2174813280	-0.1720562718
O	-0.3145107931	-2.5539225683	-0.4442773678
H	-2.9297665451	-0.7870333295	-0.4697688875
H	-1.7742381243	0.1241485973	-0.8948630387
H	0.1965404938	-1.8991624369	0.0603951482
H	-0.9979484280	-2.0149361692	-0.8709462534
O	3.0870723458	0.5310670914	2.6558724601
H	2.5884938202	-0.1723731373	2.2185671260
H	2.6178631410	1.3111683714	2.3183771885

TABLE S85. Cartesian coordinates (in angstroms) for the $BeF_3^-(H_2O)_3$ local minimum (3E - C_s) at B3LYP-D3BJ/haTZ.

Be	-0.4706386355	-1.2727824404	0.0000000000
F	0.0251394121	-1.7659437038	1.2918131532
F	0.0251394121	-1.7659437038	-1.2918131532
F	-1.4634770952	-0.1551380587	0.0000000000
O	0.0150773474	0.9259783656	2.1414595727
H	0.2638982214	-0.0119165856	2.1764220400
H	-0.7312639101	0.8861025888	1.5234924088
O	0.0150773474	0.9259783656	-2.1414595727
H	0.2638982214	-0.0119165856	-2.1764220400
H	-0.7312639101	0.8861025888	-1.5234924088
O	1.6174691979	2.2415179209	0.0000000000
H	1.1866476955	1.8310476240	-0.7676940190
H	1.1866476955	1.8310476240	0.7676940190

TABLE S86. Cartesian coordinates (in angstroms) for the $BeF_3^-(H_2O)_3$ local minimum (3F - C_1) at B3LYP-D3BJ/haTZ.

O	-3.2231575487	-0.6019734584	-0.1076078761
F	1.8336873109	-1.3453843288	0.0714289968
Be	0.5379213036	-0.6562833829	0.0424100133
F	0.4134400411	0.7975042127	-0.0859519193
F	-0.7026794939	-1.4861299043	0.1311483399
O	-2.0533371545	2.1305518573	0.1430807803
H	-3.2880068648	-0.5000277179	-1.0608499700
H	-2.3184174301	-0.9579800647	0.0307024115
H	-1.1462653911	1.7871964798	0.0648944526
H	-2.5849382368	1.3223136613	0.1966825649
O	0.8671640612	-3.9764491551	0.1928171875
H	1.4678220930	-3.2134187201	0.1519665737
H	0.0139649102	-3.5243760789	0.2103932449

TABLE S87. Cartesian coordinates (in angstroms) for the $BeF_3^-(H_2O)_3$ local minimum (3G - C_{2v}) at B3LYP-D3BJ/haTZ.

O	0.0000000000	1.7904023783	-1.0693348301
F	-1.2542162276	0.0000000000	0.7731778718
Be	0.0000000000	0.0000000000	1.5841655967
F	0.0000000000	0.0000000000	3.0319818733
F	1.2542162276	0.0000000000	0.7731778718
O	0.0000000000	-1.7904023783	-1.0693348301
H	0.7466975156	1.3636869960	-0.6208937901
H	-0.7466975156	1.3636869960	-0.6208937901
H	0.7466975156	-1.3636869960	-0.6208937901
H	-0.7466975156	-1.3636869960	-0.6208937901
O	0.0000000000	0.0000000000	-3.3980948161
H	0.0000000000	0.7541849649	-2.7856627385
H	0.0000000000	-0.7541849649	-2.7856627385

TABLE S88. Cartesian coordinates (in angstroms) for the $BeF_3^-(H_2O)_3$ local minimum (3H - C_1) at B3LYP-D3BJ/haTZ.

O	-2.1663664630	-0.2533760072	-0.1623970974
F	-0.0366722947	1.1895576496	1.2161873214
Be	0.5738670261	0.7443324113	-0.0610440422
F	1.7129403682	-0.1932827150	-0.0212786709
F	-0.0541619805	1.1486769510	-1.3279443084
O	-0.1716147927	-2.4986408347	-0.2559784170
H	-1.8354349809	0.1708388278	0.6421473558
H	-1.6961650478	0.2437185715	-0.8534708332
H	0.6001230520	-1.9170074384	-0.1967683962
H	-0.9249565297	-1.8874917948	-0.2289067848
O	2.2295280175	0.3898660753	2.7620174073
H	2.4094138879	-0.0295401112	1.9084473308
H	1.3639437375	0.7857394148	2.5806311347

TABLE S89. Cartesian coordinates (in angstroms) for the $BeF_3^-(H_2O)_3$ local minimum (3I - C_s) at B3LYP-D3BJ/haTZ.

Be	0.1062110896	1.7778567942	-1.1550891623
F	-0.6583228687	1.2699710785	-0.0188657018
F	1.5553467271	2.0908445273	-1.0491458522
F	-0.5418319139	2.1147963677	-2.4495741531
O	0.2716229547	4.6587470788	-1.3535253164
H	-0.2065794165	4.1216076726	-2.0027435869
H	1.0459337882	4.1073027413	-1.1663556394
O	1.9318685860	1.9505143280	-3.8861068367
H	0.9702529803	2.0042688707	-3.7837975523
H	2.2066061617	1.9901485028	-2.9582007476
O	-1.3471231172	3.8741204990	1.0571703503
H	-1.2341473407	2.9284722717	0.8718124847
H	-0.8600796698	4.3001788343	0.3350968009

TABLE S90. Cartesian coordinates (in angstroms) for the $BeF_3^-(H_2O)_3$ local minimum (3K - C_s) at B3LYP-D3BJ/haTZ.

Be	0.2934414328	-1.9675635257	0.0000000000
F	0.3829194775	-3.4191312374	0.0000000000
F	0.2337722785	-1.2009090378	-1.2702491500
F	0.2337722785	-1.2009090378	1.2702491500
O	-0.0657454318	1.2616589892	-2.2069821018
O	-0.0657454318	1.2616589892	2.2069821018
H	0.1058079975	0.3604299431	1.8491780365
H	-1.0198294245	1.2929286292	2.3126135429
H	0.1058079975	0.3604299431	-1.8491780365
H	-1.0198294245	1.2929286292	-2.3126135429
O	0.1585862041	3.1557186697	0.0000000000
H	0.1755022232	2.5560853730	-0.7652927071
H	0.1755022232	2.5560853730	0.7652927071

VII. B3LYP-D3BJ VIBRATIONAL FREQUENCIES

TABLE S91. Harmonic vibrational frequencies (ω in cm^{-1}) along with their corresponding irreducible representations and infrared intensities (IR in km mol^{-1}) computed for the BeF_3^- monomer at B3LYP-D3BJ/haTZ.

Irrep.	ω	IR
A_1'	597.8	0.0
A_2''	531.7	112.6
E'	1055.8	358.6
E'	1055.8	358.6
E'	319.8	8.4
E'	319.8	8.4

TABLE S92. Harmonic vibrational frequencies (ω in cm^{-1}) along with their corresponding irreducible representations and infrared intensities (IR in km mol^{-1}) computed for the $BeF_3^-(H_2O)_1$ global minimum (1A - C_{2v}) at B3LYP-D3BJ/haTZ.

Irrep.	ω	IR
A_1	3685.4	236.5
A_1	1710.1	189.8
A_1	1092.0	431.0
A_1	597.2	0.8
A_1	338.0	24.2
A_1	189.5	9.7
A_2	437.0	0.0
B_1	708.0	181.0
B_1	515.7	70.0
B_1	36.5	1.1
B_2	3703.3	160.5
B_2	1022.3	280.8
B_2	367.0	66.4
B_2	321.5	7.2
B_2	104.1	2.0

TABLE S93. Harmonic vibrational frequencies (ω in cm^{-1}) along with their corresponding irreducible representations and infrared intensities (IR in km mol^{-1}) computed for the $BeF_3{}^-(H_2O)_2$ global minimum (2A - C_{2v}) at B3LYP-D3BJ/haTZ.

Irrep.	ω	IR
A_1	3753.3	81.7
A_1	3694.8	280.8
A_1	1711.7	79.7
A_1	1030.2	276.6
A_1	597.6	0.4
A_1	353.5	117.7
A_1	330.8	5.0
A_1	173.8	1.8
A_1	70.6	2.4
A_2	660.3	0.0
A_2	404.4	0.0
A_2	38.6	0.0
B_1	676.9	363.3
B_1	499.0	31.0
B_1	413.0	2.4
B_1	30.8	2.3
B_2	3745.7	196.9
B_2	3691.7	140.0
B_2	1696.8	296.5
B_2	1086.9	431.3
B_2	346.9	41.6
B_2	341.6	19.7
B_2	184.7	12.2
B_2	115.1	0.8

TABLE S94. Harmonic vibrational frequencies (ω in cm^{-1}) along with their corresponding irreducible representations and infrared intensities (IR in km mol^{-1}) computed for the $BeF_3{}^-(H_2O)_2$ local minimum (2B - C_1) at B3LYP-D3BJ/haTZ.

Irrep.	ω	IR
A	3861.4	34.7
A	3714.9	295.0
A	3603.5	438.7
A	3397.9	718.8
A	1708.7	107.6
A	1680.5	52.6
A	1108.6	445.3
A	1016.0	343.5
A	821.1	122.5
A	700.7	140.7
A	605.3	3.9
A	595.2	8.7
A	512.2	91.7
A	501.5	48.9
A	410.3	36.5
A	349.9	8.1
A	324.6	15.3
A	212.2	25.8
A	184.2	67.5
A	160.6	41.3
A	132.4	25.2
A	61.1	0.3
A	34.3	1.8
A	22.4	1.6

TABLE S95. Harmonic vibrational frequencies (ω in cm^{-1}) along with their corresponding irreducible representations and infrared intensities (IR in km mol^{-1}) computed for the $BeF_3^-(H_2O)_2$ local minimum (2D - C_{2v}) at B3LYP-D3BJ/haTZ.

Irrep.	ω	IR
A_1	3724.3	118.4
A_1	1713.9	116.0
A_1	1118.2	433.0
A_1	668.1	225.3
A_1	591.0	5.2
A_1	337.1	16.3
A_1	170.4	3.3
A_1	53.2	0.6
A_2	3752.5	0.0
A_2	377.3	0.0
A_2	315.9	0.0
A_2	63.3	0.0
B_1	3716.3	168.8
B_1	1681.5	233.5
B_1	608.2	215.0
B_1	467.4	15.9
B_1	169.4	7.7
B_1	43.9	0.7
B_2	3767.9	231.6
B_2	991.1	227.8
B_2	433.1	1.4
B_2	357.2	138.8
B_2	311.1	7.1
B_2	99.5	4.9

TABLE S96. Harmonic vibrational frequencies (ω in cm^{-1}) along with their corresponding irreducible representations and infrared intensities (IR in km mol^{-1}) computed for the $BeF_3^-(H_2O)_2$ local minimum (2E - C_s) at B3LYP-D3BJ/haTZ.

Irrep.	ω	IR
A′	3718.7	351.2
A′	3670.8	16.0
A′	3653.0	281.6
A′	1706.9	175.4
A′	1696.3	98.4
A′	1075.9	322.3
A′	725.8	239.4
A′	604.1	6.1
A′	534.4	138.5
A′	484.1	71.7
A′	325.1	11.0
A′	194.2	10.7
A′	165.1	7.8
A′	110.7	1.3
A′	71.8	0.3
A″	3706.3	159.7
A″	1041.1	284.3
A″	665.3	61.4
A″	536.0	57.3
A″	468.5	58.7
A″	361.2	13.1
A″	307.1	4.4
A″	91.0	0.0
A″	50.8	0.4

TABLE S97. Harmonic vibrational frequencies (ω in cm^{-1}) along with their corresponding irreducible representations and infrared intensities (IR in km mol^{-1}) computed for the $BeF_3{}^{-}(H_2O)_3$ global minimum (3A - C_3) at B3LYP-D3BJ/haTZ.

Irrep.	ω	IR
A	3651.1	375.0
A	3651.1	374.6
A	3650.2	810.1
A	3594.8	38.8
A	3594.8	38.7
A	3565.8	186.3
A	1714.1	137.1
A	1691.8	49.3
A	1691.8	49.3
A	1060.3	297.5
A	1060.3	297.5
A	913.0	3.4
A	726.0	6.8
A	722.0	272.3
A	721.9	272.3
A	605.6	2.0
A	577.0	106.8
A	577.0	107.0
A	572.0	128.7
A	501.1	9.1
A	501.1	9.1
A	493.7	105.0
A	312.6	6.4
A	312.6	6.4
A	202.5	16.2
A	194.3	8.5
A	194.2	8.5
A	173.8	4.6
A	136.9	9.0
A	136.8	9.0
A	68.0	0.1
A	68.0	0.1
A	56.4	0.3

TABLE S98. Harmonic vibrational frequencies (ω in cm^{-1}) along with their corresponding irreducible representations and infrared intensities (IR in km mol^{-1}) computed for the $BeF_3^-(H_2O)_3$ local minimum (3B - C_1) at B3LYP-D3BJ/haTZ.

Irrep.	ω	IR
A	3733.9	241.9
A	3702.6	287.7
A	3698.3	250.0
A	3626.6	207.7
A	3591.6	250.4
A	3516.3	426.6
A	1715.4	99.0
A	1696.9	94.5
A	1691.0	97.9
A	1123.0	453.9
A	1003.6	323.3
A	872.8	14.2
A	790.2	170.2
A	702.2	279.8
A	659.2	109.6
A	602.5	15.4
A	546.2	22.8
A	527.6	105.0
A	511.0	78.7
A	475.4	50.6
A	446.2	10.0
A	386.2	25.1
A	339.6	15.6
A	324.6	17.1
A	203.9	19.0
A	193.5	12.7
A	173.8	11.6
A	140.1	10.0
A	114.5	2.3
A	100.5	1.9
A	74.2	0.4
A	59.6	0.4
A	32.1	1.4

TABLE S99. Harmonic vibrational frequencies (ω in cm^{-1}) along with their corresponding irreducible representations and infrared intensities (IR in km mol^{-1}) computed for the $BeF_3{}^-(H_2O)_3$ local minimum (3C - D_{3h}) at B3LYP-D3BJ/haTZ.

Irrep.	ω	IR
A_1''	380.0	0.0
A_2''	647.3	544.0
A_2''	480.8	3.6
A_2''	24.0	3.7
E''	616.9	0.0
E''	616.9	0.0
E''	391.2	0.0
E''	391.2	0.0
E''	37.3	0.0
E''	37.3	0.0
A	3768.4	181.8
A	3768.4	181.8
A	3724.3	281.0
A	3724.3	281.0
A	1691.2	282.5
A	1691.2	282.5
A	1059.7	349.8
A	1059.7	349.7
A	343.4	96.6
A	343.4	96.6
A	333.1	40.7
A	333.1	40.7
A	173.3	5.4
A	173.3	5.4
A	66.9	2.6
A	66.9	2.6
B	3757.8	0.0
B	3729.1	0.0
B	1712.3	0.0
B	597.9	0.0
B	325.2	0.0
B	157.3	0.0
B	120.9	0.0

TABLE S100. Harmonic vibrational frequencies (ω in cm^{-1}) along with their corresponding irreducible representations and infrared intensities (IR in km mol^{-1}) computed for the $BeF_3{}^{-}(H_2O)_3$ local minimum (3D - C_1) at B3LYP-D3BJ/haTZ.

Irrep.	ω	IR
A	3777.2	91.6
A	3712.0	415.9
A	3690.2	224.4
A	3652.2	291.4
A	3443.0	678.5
A	1707.2	130.0
A	1697.1	145.6
A	1677.1	58.8
A	1084.0	433.6
A	1042.2	346.5
A	798.2	134.7
A	669.2	260.4
A	647.6	78.5
A	605.7	2.5
A	576.0	5.8
A	496.2	42.4
A	488.7	45.7
A	394.8	36.6
A	390.8	4.2
A	354.7	19.8
A	341.7	63.8
A	326.7	21.6
A	216.8	43.6
A	180.9	68.6
A	169.9	12.8
A	161.3	16.9
A	134.1	15.8
A	88.3	0.8
A	50.7	1.0
A	42.6	0.9
A	27.2	1.6
A	19.1	1.2

TABLE S101. Harmonic vibrational frequencies (ω in cm^{-1}) along with their corresponding irreducible representations and infrared intensities (IR in km mol^{-1}) computed for the $BeF_3^-(H_2O)_3$ local minimum (3E - C_s) at B3LYP-D3BJ/haTZ.

Irrep.	ω	IR
A′	3731.2	169.6
A′	3692.8	246.3
A′	3627.3	332.1
A′	1707.6	184.7
A′	1694.4	132.5
A′	1018.2	260.2
A′	735.1	205.5
A′	686.4	63.3
A′	598.0	5.8
A′	492.2	96.9
A′	459.4	64.9
A′	348.5	48.4
A′	321.4	16.2
A′	206.1	9.0
A′	166.5	7.4
A′	101.2	1.7
A′	64.7	0.5
A′	46.2	1.7
A″	3721.7	287.7
A″	3706.7	221.2
A″	3665.8	48.6
A″	1688.1	90.4
A″	1098.9	352.6
A″	706.3	329.9
A″	658.2	28.8
A″	521.2	0.5
A″	429.1	0.0
A″	340.5	32.2
A″	332.2	2.5
A″	188.8	11.3
A″	132.4	3.0
A″	101.4	0.1
A″	48.2	0.4

TABLE S102. Harmonic vibrational frequencies (ω in cm^{-1}) along with their corresponding irreducible representations and infrared intensities (IR in km mol^{-1}) computed for the $BeF_3^-(H_2O)_3$ local minimum (3F - C_1) at B3LYP-D3BJ/haTZ.

Irrep.	ω	IR
A	3862.8	41.7
A	3793.5	86.6
A	3716.3	343.9
A	3671.7	225.5
A	3637.5	360.9
A	3471.8	662.0
A	1706.5	28.4
A	1698.2	251.7
A	1675.7	61.8
A	1107.0	461.6
A	1018.0	315.6
A	781.0	124.9
A	676.9	212.4
A	654.8	116.5
A	603.8	2.1
A	580.7	5.8
A	497.3	47.7
A	488.2	45.6
A	387.9	40.7
A	373.6	4.9
A	355.9	19.4
A	338.7	86.7
A	323.5	6.9
A	204.4	22.8
A	189.9	37.2
A	167.9	34.7
A	158.2	34.4
A	132.1	15.9
A	87.7	2.1
A	49.2	0.7
A	37.8	2.1
A	32.8	0.3
A	16.7	3.1

TABLE S103. Harmonic vibrational frequencies (ω in cm^{-1}) along with their corresponding irreducible representations and infrared intensities (IR in km mol^{-1}) computed for the $BeF_3{}^-(H_2O)_3$ local minimum (3G - C_{2v}) at B3LYP-D3BJ/haTZ.

Irrep.	ω	IR
A_1	3704.6	128.4
A_1	3642.6	375.6
A_1	1713.1	189.3
A_1	1705.9	104.8
A_1	1131.5	478.5
A_1	732.3	188.8
A_1	594.5	0.0
A_1	342.0	19.7
A_1	201.9	1.4
A_1	179.7	15.1
A_1	73.6	0.1
A_2	3723.4	0.0
A_2	633.3	0.0
A_2	407.0	0.0
A_2	311.4	0.0
A_2	69.1	0.0
B_1	3742.0	260.2
B_1	982.9	218.8
B_1	752.5	115.1
B_1	468.3	46.7
B_1	360.8	97.6
B_1	313.2	6.0
B_1	109.8	0.8
B_1	49.9	4.3
B_2	3707.6	378.5
B_2	3665.3	33.7
B_2	1675.3	176.6
B_2	666.4	324.6
B_2	504.1	15.7
B_2	442.7	18.4
B_2	183.3	14.7
B_2	126.5	0.6
B_2	44.1	1.7

TABLE S104. Harmonic vibrational frequencies (ω in cm^{-1}) along with their corresponding irreducible representations and infrared intensities (IR in km mol^{-1}) computed for the $BeF_3^-(H_2O)_3$ local minimum (3H - C_1) at B3LYP-D3BJ/haTZ.

Irrep.	ω	IR
A	3764.8	179.5
A	3759.9	141.3
A	3745.8	133.0
A	3719.9	199.4
A	3672.6	163.7
A	3648.2	219.6
A	1706.7	129.8
A	1693.5	77.4
A	1687.8	236.1
A	1080.5	343.6
A	1037.3	260.0
A	711.8	204.0
A	658.2	188.5
A	629.3	119.5
A	598.2	8.4
A	513.7	115.8
A	481.9	81.0
A	466.2	26.4
A	443.7	64.3
A	388.8	2.2
A	346.7	15.5
A	334.8	59.4
A	328.2	19.8
A	313.1	4.1
A	190.3	9.1
A	170.8	4.4
A	159.5	5.8
A	105.3	0.9
A	96.5	2.1
A	75.5	0.3
A	65.1	1.3
A	46.5	0.4
A	30.6	1.1

TABLE S105. Harmonic vibrational frequencies (ω in cm^{-1}) along with their corresponding irreducible representations and infrared intensities (IR in km mol^{-1}) computed for the $BeF_3{}^-(H_2O)_3$ local minimum (3I - C_s) at B3LYP-D3BJ/haTZ.

Irrep.	ω	IR
A′	3731.1	320.7
A′	3719.7	129.9
A′	3701.5	38.2
A′	3668.3	260.5
A′	1708.9	46.6
A′	1702.1	159.1
A′	1675.6	263.8
A′	1100.4	378.1
A′	676.1	290.3
A′	632.6	162.8
A′	597.1	4.5
A′	506.8	94.8
A′	446.1	48.8
A′	336.6	17.8
A′	176.2	15.4
A′	169.7	1.2
A′	168.7	3.8
A′	121.5	0.7
A′	71.2	0.0
A′	36.5	1.8
A″	3765.3	237.2
A″	3750.3	1.3
A″	1012.1	232.3
A″	652.4	87.8
A″	529.8	35.4
A″	441.5	52.2
A″	410.2	12.5
A″	340.9	72.1
A″	313.8	5.4
A″	304.9	1.6
A″	102.0	2.2
A″	56.5	0.1
A″	39.7	2.2

TABLE S106. Harmonic vibrational frequencies (ω in cm^{-1}) along with their corresponding irreducible representations and infrared intensities (IR in km mol^{-1}) computed for the $BeF_3{}^-(H_2O)_3$ local minimum (3K - C_s) at B3LYP-D3BJ/haTZ.

Irrep.	ω	IR
A′	3868.0	68.8
A′	3614.5	186.1
A′	3436.7	1510.0
A′	1701.3	48.0
A′	1681.8	140.9
A′	1110.9	467.2
A′	795.9	21.6
A′	718.8	157.8
A′	613.4	16.5
A′	510.0	104.9
A′	408.9	67.8
A′	315.8	14.2
A′	238.1	11.7
A′	196.2	54.0
A′	165.6	4.1
A′	64.7	0.3
A′	35.7	0.4
A′	16.5	3.3
A″	3867.4	8.4
A″	3666.2	605.8
A″	3413.1	142.2
A″	1677.2	7.6
A″	1018.0	404.6
A″	782.3	176.6
A″	664.5	9.1
A″	531.2	26.9
A″	392.2	2.0
A″	362.2	0.1
A″	203.7	50.4
A″	161.4	105.4
A″	141.7	45.1
A″	37.5	0.0
A″	14.5	3.2

VIII. B3LYP OPTIMIZED STRUCTURES

TABLE S107. Cartesian coordinates (in angstroms) for the BeF_3^- monomer at B3LYP/aTZ.

Be	0.0000000000	0.0000000000	0.0000000000
F	0.0000000000	0.0000000000	1.4782987444
F	1.2802442671	0.0000000000	-0.7391493722
F	-1.2802442671	0.0000000000	-0.7391493722

TABLE S108. Cartesian coordinates (in angstroms) for the $BeF_3^-(H_2O)_1$ global minimum (1A - C_{2v}) at B3LYP/haTZ.

Be	0.0000000000	0.0000000000	0.7940781771
F	1.2700491136	0.0000000000	0.0237133234
F	0.0000000000	0.0000000000	2.2581275442
F	-1.2700491136	0.0000000000	0.0237133234
H	0.7384112161	0.0000000000	-1.8765698323
H	-0.7384112161	0.0000000000	-1.8765698323
O	0.0000000000	0.0000000000	-2.5070733036

TABLE S109. Cartesian coordinates (in angstroms) for the $BeF_3^-(H_2O)_2$ global minimum (2A - C_{2v}) at B3LYP/haTZ.

Be	0.0000000000	0.0000000000	-0.6265600000
F	0.0000000000	1.2890440000	-1.3386240000
F	0.0000000000	-1.2890440000	-1.3386240000
F	0.0000000000	0.0000000000	0.8620560000
H	0.0000000000	2.6684960000	0.0542650000
H	0.0000000000	2.0071100000	1.3819240000
O	0.0000000000	2.8958420000	0.9981610000
O	0.0000000000	-2.8958420000	0.9981610000
H	0.0000000000	-2.6684960000	0.0542650000
H	0.0000000000	-2.0071100000	1.3819240000

TABLE S110. Cartesian coordinates (in angstroms) for the $BeF_3^-(H_2O)_2$ local minimum (2B - C_1) at B3LYP/haTZ.

Be	-1.3516794200	-0.0395672486	0.0051845829
F	-0.6326784992	-1.3253724733	-0.1139600528
F	-2.8075967395	0.0407440174	0.0114428482
F	-0.5287577775	1.2030065862	0.1067776611
O	2.1593759967	-1.5179767995	0.1152029989
O	2.1110596936	1.4987397461	-0.0292159037
H	1.1330969453	1.4214635087	0.0701237287
H	1.1895056094	-1.5710606992	0.0334824889
H	2.3145504128	-0.5655184254	0.1997808773
H	2.2473467783	1.4793727878	-0.9800292293

TABLE S111. Cartesian coordinates (in angstroms) for the $BeF_3^-(H_2O)_2$ local minimum (2D - C_{2v}) at B3LYP/haTZ.

Be	0.1485494750	0.0000000000	0.0000000000
F	1.6018077594	0.0000000000	0.0000000000
F	-0.6524672453	-1.2591976265	0.0000000000
F	-0.6524672453	1.2591976265	0.0000000000
O	-2.3710252572	0.0000000000	1.9897625649
H	-2.0084280574	0.7450606570	1.4887175397
H	-2.0084280574	-0.7450606570	1.4887175397
O	-2.3710252571	0.0000000000	-1.9897625649
H	-2.0084280574	0.7450606570	-1.4887175397
H	-2.0084280574	-0.7450606570	-1.4887175397

TABLE S112. Cartesian coordinates (in angstroms) for the $BeF_3^-(H_2O)_2$ local minimum (2E - C_s) at B3LYP/haTZ.

Be	0.6707041873	0.8217121118	0.0000000000
F	0.0390412448	1.2490051402	1.2683951664
F	0.0390412448	1.2490051402	-1.2683951664
F	1.8001773943	-0.1219656981	0.0000000000
O	-2.0575060023	-0.2011082098	0.0000000000
H	-1.6445287505	0.2585067224	0.7491063706
H	-1.6445287505	0.2585067224	-0.7491063706
O	0.0290008762	-2.4639566579	0.0000000000
H	0.7688972814	-1.8353202584	0.0000000000
H	-0.7525147255	-1.8901620127	0.0000000000

TABLE S113. Cartesian coordinates (in angstroms) for the $BeF_3^-(H_2O)_3$ global minimum (3A - C_3) at B3LYP/haTZ.

Be	0.0007269561	-0.0002004147	-1.3882751347
F	1.4726906626	-0.1050832832	-1.3367901669
F	-0.6444796999	1.3270125303	-1.3375102228
F	-0.8261407907	-1.2225147573	-1.3373780402
O	1.7078601262	-0.0307802182	1.4631922626
O	-0.8278865839	1.4950848283	1.4621503189
O	-0.8812290827	-1.4639589431	1.4624942744
H	1.8431246866	-0.0752817329	0.4990886244
H	-0.8566148621	1.6338868074	0.4979458067
H	-0.9867772402	-1.5587477683	0.4983109140
H	1.0609140243	0.6876260362	1.5697403521
H	-1.1264790159	0.5756299208	1.5690961559
H	0.0642906491	-1.2626730055	1.5696211907

TABLE S114. Cartesian coordinates (in angstroms) for the $BeF_3^-(H_2O)_3$ local minimum (3B - C_1) at B3LYP/haTZ.

F	-0.3263089786	-0.0953933212	2.8873515033
Be	0.2463439958	0.1292183035	1.5719362304
F	0.7193955414	1.4596248620	1.1219592735
F	0.3943877348	-0.9899913547	0.5889177015
O	-0.1567830731	-0.3964147128	-2.2108356897
H	0.3535288973	0.4276510764	-2.1077222700
H	-0.2531902071	-0.6929427707	-1.2921366938
O	1.8137126181	1.5780406769	-1.3793135703
H	1.4352941224	1.6562653454	-0.4794088365
H	2.3486323725	0.7715509363	-1.3268461079
O	2.4945445167	-1.3770669515	-1.2369738043
H	1.9432986675	-1.3566305316	-0.4344342259
H	1.8181957924	-1.3331745581	-1.9287535103

TABLE S115. Cartesian coordinates (in angstroms) for the $BeF_3^-(H_2O)_3$ local minimum (3C - D_{3h}) at B3LYP/haTZ.

Be	0.0000000000	0.0000000000	0.0000000000
F	0.0000000043	1.4777070301	0.0000000000
F	1.2797318253	-0.7388535188	0.0000000000
F	-1.2797318296	-0.7388535113	0.0000000000
H	-1.9840856844	2.0046140895	0.0000000000
H	-2.7280895642	0.7159615772	0.0000000000
O	-2.8925434854	1.6700107713	0.0000000000
H	2.7280895684	0.7159615612	0.0000000000
H	1.9840856961	2.0046140778	0.0000000000
H	-0.7440038841	-2.7205756507	0.0000000000
H	0.7440038681	-2.7205756550	0.0000000000
O	-0.0000000098	-3.3400215256	0.0000000000
O	2.8925434952	1.6700107543	0.0000000000

TABLE S116. Cartesian coordinates (in angstroms) for the $BeF_3^-(H_2O)_3$ local minimum (3D - C_1) at B3LYP/haTZ.

O	-2.3824747350	-0.5637368927	-1.2796161495
F	1.1862284486	2.1100399644	1.3072865304
Be	0.4731441523	0.9853074423	0.6899153743
F	0.9576600351	-0.3979712139	0.8994518711
F	-0.7381173212	1.2080309263	-0.1316491654
O	-0.3351302325	-2.5871446295	-0.4178450097
H	-2.9999887687	-0.7408622045	-0.5652525042
H	-1.7803707565	0.1207003775	-0.9106281558
H	0.1885729652	-1.9306382193	0.0710737988
H	-1.0148525210	-2.0473642379	-0.8491287026
O	3.1447035827	0.5650733816	2.6472920596
H	2.6701742315	-0.1557139735	2.2123048583
H	2.6348949196	1.3276702793	2.3284371946

TABLE S117. Cartesian coordinates (in angstroms) for the $BeF_3^-(H_2O)_3$ local minimum (3E - C_s) at B3LYP/haTZ.

Be	-0.5007677250	-1.3049725481	0.0000000000
F	-0.0109421973	-1.8030766215	1.2926702305
F	-0.0109421973	-1.8030766215	-1.2926702305
F	-1.4731526552	-0.1688550180	0.0000000000
O	0.0288719859	0.9158963279	2.1482030097
H	0.2735034884	-0.0224325451	2.1893896136
H	-0.7193932179	0.8756079288	1.5325450117
O	0.0288719859	0.9158963279	-2.1482030097
H	0.2735034884	-0.0224325451	-2.1893896136
H	-0.7193932179	0.8756079288	-1.5325450117
O	1.6192463527	2.3149597199	0.0000000000
H	1.2064724546	1.8855058329	-0.7667589888
H	1.2064724546	1.8855058329	0.7667589888

TABLE S118. Cartesian coordinates (in angstroms) for the $BeF_3^-(H_2O)_3$ local minimum (3F - C_1) at B3LYP/haTZ.

O	-3.2405728583	-0.5885331286	-0.0753908201
F	1.8339854798	-1.3561756849	0.0930238346
Be	0.5440787042	-0.6535062424	0.0713494104
F	0.4336281643	0.8052076598	-0.0144354075
F	-0.7042212666	-1.4756891512	0.1249111151
O	-2.0471148450	2.1749312527	0.0872335315
H	-3.3567227976	-0.5725209934	-1.0290214983
H	-2.3321411235	-0.9404722355	0.0478706044
H	-1.1395510443	1.8263443390	0.0524944021
H	-2.5846611276	1.3708982094	0.1429349377
O	0.9003769704	-4.0064764517	0.1617156563
H	1.4775090817	-3.2241958098	0.1375598624
H	0.0326042626	-3.5842683634	0.1808691714

TABLE S119. Cartesian coordinates (in angstroms) for the $BeF_3^-(H_2O)_3$ local minimum (3G - C_{2v}) at B3LYP/haTZ.

O	0.0000000000	1.8083319282	-1.0729045128
F	-1.2535924227	0.0000000000	0.7945996242
Be	0.0000000000	0.0000000000	1.6067067093
F	0.0000000000	0.0000000000	3.0554619510
F	1.2535924227	0.0000000000	0.7945996242
O	0.0000000000	-1.8083319282	-1.0729045128
H	0.7469520597	1.3865529016	-0.6205228116
H	-0.7469520597	1.3865529016	-0.6205228116
H	0.7469520597	-1.3865529016	-0.6205228116
H	-0.7469520597	-1.3865529016	-0.6205228116
O	0.0000000000	0.0000000000	-3.4250975592
H	0.0000000000	0.7546608280	-2.8137659887
H	0.0000000000	-0.7546608280	-2.8137659887

TABLE S120. Cartesian coordinates (in angstroms) for the $BeF_3^-(H_2O)_3$ local minimum (3I - C_s) at B3LYP/haTZ.

Be	-1.3009605897	-1.0682041366	-0.0537428939
F	-2.2642530634	0.0309508885	-0.0195286649
F	-0.7440261038	-1.6641204932	1.1888779494
F	-0.7360773506	-1.5787412667	-1.3303231933
O	-0.3588037685	2.2346326225	0.0611776658
H	-1.1447876283	1.6657556578	0.0394133183
H	0.3716979411	1.5983887238	0.0419160972
O	1.4797819019	-0.2039605426	-0.0156776423
H	1.0785173229	-0.6889672468	0.7205810800
H	1.0832681430	-0.6379165157	-0.7856001405
O	-0.0412238507	-4.1263005625	-0.1534137532
H	-0.1963642316	-3.5507228481	0.6098269014
H	-0.1916703224	-3.5003300808	-0.8768925147

TABLE S121. Cartesian coordinates (in angstroms) for the $BeF_3{}^-(H_2O)_3$ local minimum (3J - C_2) at B3LYP/haTZ.

Be	0.0000000000	0.0000000000	-1.8709887316
F	0.0000000000	0.0000000000	-3.3257188883
F	-0.2007237854	1.2546837293	-1.1007037648
F	0.2007237854	-1.2546837293	-1.1007037648
O	0.0130300651	2.2364967207	1.3686107840
O	-0.0130300651	-2.2364967207	1.3686107840
H	0.1190379982	-1.8628851625	0.4675792874
H	-0.9528681213	-2.4318852284	1.4049932928
H	-0.1190379982	1.8628851625	0.4675792874
H	0.9528681213	2.4318852284	1.4049932928
O	0.0000000000	0.0000000000	3.2789181752
H	-0.0821547121	0.7609813304	2.6795025230
H	0.0821547121	-0.7609813304	2.6795025230

TABLE S122. Cartesian coordinates (in angstroms) for the $BeF_3{}^-(H_2O)_3$ local minimum (3K - C_s) at B3LYP/haTZ.

Be	0.2712486897	-1.9729880806	0.0000000000
F	0.3199376558	-3.4270235897	0.0000000000
F	0.2339971036	-1.2042783829	-1.2709974615
F	0.2339971036	-1.2042783829	1.2709974615
O	-0.0193910841	1.2633802080	-2.2438112564
O	-0.0193910841	1.2633802080	2.2438112564
H	0.1331074054	0.3643149915	1.8730748390
H	-0.9587338771	1.2777152908	2.4436225778
H	0.1331074054	0.3643149915	-1.8730748390
H	-0.9587338771	1.2777152908	-2.4436225778
O	0.0787509959	3.1671490440	0.0000000000
H	0.1230329816	2.5700050557	-0.7657992740
H	0.1230329816	2.5700050557	0.7657992740

IX. B3LYP VIBRATIONAL FREQUENCIES

TABLE S123. Harmonic vibrational frequencies (ω in cm^{-1}) along with their corresponding irreducible representations and infrared intensities (IR in km mol^{-1}) computed for the $BeF_3{}^-$ monomer at B3LYP/haTZ.

Irrep.	ω	IR
A_1'	597.4	0.0
A_2''	531.9	112.6
E'	1055.2	358.7
E'	1055.2	358.7
E'	319.6	8.4
E'	319.6	8.4

TABLE S124. Harmonic vibrational frequencies (ω in cm^{-1}) along with their corresponding irreducible representations and infrared intensities (IR in km mol^{-1}) computed for the $BeF_3{}^-(H_2O)_1$ global minimum (1A - C_{2v}) at B3LYP/haTZ.

Irrep.	ω	IR
A_1	3688.0	226.7
A_1	1707.7	188.0
A_1	1091.0	430.1
A_1	596.6	0.8
A_1	336.6	23.3
A_1	182.2	10.1
A_2	432.2	0.0
B_1	703.4	183.0
B_1	516.1	69.4
B_1	37.5	1.1
B_2	3707.6	156.1
B_2	1020.8	282.1
B_2	356.6	68.3
B_2	320.4	5.7
B_2	96.5	2.2

TABLE S125. Harmonic vibrational frequencies (ω in cm^{-1}) along with their corresponding irreducible representations and infrared intensities (IR in km mol^{-1}) computed for the $BeF_3{}^{-}(H_2O)_2$ global minimum (2A - C_{2v}) at B3LYP/haTZ.

Irrep.	ω	IR
A_1	3758.2	80.5
A_1	3694.6	278.4
A_1	1708.9	76.8
A_1	1029.0	277.5
A_1	596.9	0.3
A_1	343.5	124.0
A_1	327.9	0.0
A_1	166.2	1.9
A_1	64.5	2.6
A_2	656.2	0.0
A_2	398.5	0.0
A_2	39.9	0.0
B_1	672.6	366.3
B_1	499.8	30.3
B_1	407.1	3.3
B_1	31.9	2.2
B_2	3751.3	179.7
B_2	3691.6	134.8
B_2	1694.5	294.1
B_2	1084.8	431.2
B_2	339.4	45.2
B_2	336.4	13.7
B_2	176.8	13.0
B_2	102.8	0.6

TABLE S126. Harmonic vibrational frequencies (ω in cm^{-1}) along with their corresponding irreducible representations and infrared intensities (IR in km mol^{-1}) computed for the $BeF_3^-(H_2O)_2$ local minimum (2B - C_1) at B3LYP/haTZ.

Irrep.	ω	IR
A	3863.2	35.1
A	3718.0	294.1
A	3609.7	410.7
A	3401.3	715.2
A	1706.7	101.4
A	1680.3	55.8
A	1107.1	442.6
A	1012.9	348.1
A	815.5	121.0
A	689.6	151.1
A	603.6	5.0
A	580.0	10.1
A	512.8	89.2
A	497.3	49.0
A	408.9	33.9
A	346.8	7.5
A	323.2	15.8
A	206.8	27.6
A	177.8	81.8
A	153.8	29.6
A	125.3	10.2
A	62.9	0.1
A	37.6	1.6
A	27.4	1.7

TABLE S127. Harmonic vibrational frequencies (ω in cm^{-1}) along with their corresponding irreducible representations and infrared intensities (IR in km mol^{-1}) computed for the $BeF_3{}^{-}(H_2O)_2$ local minimum (2D - C_{2v}) at B3LYP/haTZ.

Irrep.	ω	IR
A_1	3725.8	107.0
A_1	1709.6	111.0
A_1	1116.2	431.0
A_1	658.9	226.9
A_1	590.2	7.5
A_1	335.3	15.0
A_1	159.1	3.4
A_1	51.5	0.6
A_2	3757.3	0.0
A_2	373.9	0.0
A_2	305.9	0.0
A_2	45.9	0.0
B_1	3718.8	162.4
B_1	1679.5	234.5
B_1	606.0	211.0
B_1	469.3	16.3
B_1	157.2	8.1
B_1	45.3	0.6
B_2	3771.2	224.3
B_2	990.2	230.9
B_2	424.0	1.5
B_2	342.0	142.1
B_2	309.7	4.3
B_2	86.8	5.5

TABLE S128. Harmonic vibrational frequencies (ω in cm^{-1}) along with their corresponding irreducible representations and infrared intensities (IR in km mol^{-1}) computed for the $BeF_3{}^-(H_2O)_2$ local minimum (2E - C_s) at B3LYP/haTZ.

Irrep.	ω	IR
A′	3727.0	311.7
A′	3674.2	18.4
A′	3661.3	272.7
A′	1704.0	174.9
A′	1694.1	98.6
A′	1073.1	325.7
A′	713.8	231.5
A′	603.4	7.0
A′	522.3	172.3
A′	479.0	40.9
A′	324.5	10.7
A′	184.2	10.7
A′	152.6	7.6
A′	95.9	1.2
A′	71.1	0.4
A″	3713.5	153.6
A″	1040.4	285.8
A″	646.3	72.5
A″	534.2	49.8
A″	457.1	58.0
A″	353.8	14.4
A″	307.2	4.6
A″	80.2	0.0
A″	49.9	0.4

TABLE S129. Harmonic vibrational frequencies (ω in cm^{-1}) along with their corresponding irreducible representations and infrared intensities (IR in km mol^{-1}) computed for the $BeF_3{}^-(H_2O)_3$ global minimum (3A - C_3) at B3LYP/haTZ.

Irrep.	ω	IR
A	3665.8	351.2
A	3665.8	351.4
A	3660.0	697.8
A	3600.6	23.2
A	3600.5	23.1
A	3581.3	259.9
A	1710.9	136.4
A	1690.0	50.9
A	1689.7	50.9
A	1057.6	300.2
A	1057.5	300.4
A	883.1	3.0
A	715.7	6.4
A	710.8	264.8
A	710.4	265.4
A	604.2	5.1
A	560.1	144.5
A	556.7	113.0
A	555.4	112.5
A	492.6	82.7
A	491.5	9.5
A	490.9	12.3
A	312.7	6.3
A	312.6	6.2
A	190.3	17.6
A	182.0	8.1
A	181.6	8.1
A	157.5	3.1
A	123.2	8.4
A	123.0	8.4
A	68.0	0.2
A	67.8	0.2
A	58.1	0.3

TABLE S130. Harmonic vibrational frequencies (ω in cm^{-1}) along with their corresponding irreducible representations and infrared intensities (IR in km mol^{-1}) computed for the $BeF_3^-(H_2O)_3$ local minimum (3B - C_1) at B3LYP/haTZ.

Irrep.	ω	IR
A	3740.5	221.2
A	3712.8	285.6
A	3707.9	202.3
A	3632.7	205.8
A	3602.3	237.5
A	3518.8	428.1
A	1712.1	98.6
A	1695.0	94.8
A	1688.7	96.5
A	1120.8	453.4
A	999.5	325.0
A	850.2	19.2
A	781.4	160.3
A	689.8	279.0
A	644.8	108.4
A	600.0	17.8
A	530.1	14.7
A	513.6	128.4
A	509.8	65.1
A	468.1	54.9
A	432.0	10.9
A	379.4	26.3
A	336.0	17.5
A	322.8	13.8
A	194.1	19.9
A	183.4	11.3
A	164.2	11.9
A	124.5	8.5
A	103.3	2.8
A	88.9	1.9
A	73.3	0.5
A	60.6	0.4
A	34.2	1.3

TABLE S131. Harmonic vibrational frequencies (ω in cm^{-1}) along with their corresponding irreducible representations and infrared intensities (IR in km mol^{-1}) computed for the $BeF_3{}^-(H_2O)_3$ local minimum (3C - D_{3h}) at B3LYP/haTZ.

Irrep.	ω	IR
A_1''	374.1	0.0
A_2''	642.3	550.0
A_2''	481.9	2.9
E″	611.7	0.0
E″	611.7	0.0
E″	385.2	0.0
E″	385.2	0.0
A	38.9	0.0
A	38.9	0.0
A	25.2	3.5
B	3771.5	177.9
B	3771.5	177.9
B	3726.1	263.2
B	3726.1	263.2
B	1688.6	278.1
B	1688.6	278.1
B	1057.5	350.1
B	1057.4	350.3
B	339.5	68.9
B	339.5	69.0
B	321.3	67.8
B	321.3	67.7
B	163.5	5.8
B	163.5	5.8
B	60.8	2.7
B	60.8	2.7

TABLE S132. Harmonic vibrational frequencies (ω in cm^{-1}) along with their corresponding irreducible representations and infrared intensities (IR in km mol^{-1}) computed for the $BeF_3{}^{-}(H_2O)_3$ local minimum (3D - C_1) at B3LYP/haTZ.

Irrep.	ω	IR
A	3864.2	40.1
A	3784.3	84.2
A	3717.7	396.0
A	3686.2	227.2
A	3655.8	269.7
A	3447.9	671.0
A	1705.0	123.2
A	1694.9	142.3
A	1676.5	63.8
A	1080.9	431.8
A	1040.0	349.4
A	791.0	134.1
A	658.7	323.9
A	642.6	24.7
A	603.9	2.7
A	564.0	4.7
A	497.4	41.2
A	483.0	46.5
A	394.4	34.6
A	381.5	6.0
A	351.5	19.6
A	330.9	67.0
A	324.3	18.4
A	210.7	46.3
A	177.1	87.1
A	163.2	2.0
A	152.2	6.3
A	122.9	8.5
A	80.9	0.8
A	49.6	1.6
A	45.6	0.9
A	30.1	1.7
A	21.0	1.0

TABLE S133. Harmonic vibrational frequencies (ω in cm^{-1}) along with their corresponding irreducible representations and infrared intensities (IR in km mol^{-1}) computed for the $BeF_3{}^{-}(H_2O)_3$ local minimum (3E - C_s) at B3LYP/haTZ.

Irrep.	ω	IR
A′	3734.7	183.5
A′	3698.7	208.2
A′	3637.3	307.3
A′	1705.2	164.2
A′	1692.4	156.0
A′	1016.3	263.7
A′	723.4	200.3
A′	675.2	75.6
A′	597.8	6.5
A′	493.6	92.2
A′	450.5	58.7
A′	337.2	52.0
A′	320.0	15.6
A′	195.5	9.1
A′	152.6	7.0
A′	90.0	1.8
A′	65.4	0.4
A′	48.2	1.9
A″	3725.5	232.4
A″	3715.6	268.0
A″	3675.6	19.3
A″	1685.9	87.4
A″	1097.3	354.7
A″	692.0	336.0
A″	638.5	18.9
A″	507.9	2.1
A″	420.7	0.0
A″	335.4	32.1
A″	323.9	2.0
A″	177.7	11.7
A″	116.4	2.7
A″	87.9	0.3
A″	48.2	0.3

TABLE S134. Harmonic vibrational frequencies (ω in cm^{-1}) along with their corresponding irreducible representations and infrared intensities (IR in km mol^{-1}) computed for the $BeF_3^-(H_2O)_3$ local minimum (3F - C_1) at B3LYP/haTZ.

Irrep.	ω	IR
A	3863.8	42.0
A	3802.6	74.7
A	3721.4	328.9
A	3663.3	235.5
A	3642.1	344.1
A	3474.0	650.6
A	1704.7	36.8
A	1695.5	230.1
A	1675.3	68.2
A	1104.1	461.1
A	1015.6	317.9
A	775.1	123.1
A	666.4	270.1
A	653.3	64.8
A	602.1	2.3
A	566.7	5.9
A	498.6	47.8
A	482.2	46.3
A	387.2	37.8
A	360.9	10.3
A	352.4	19.0
A	329.5	91.5
A	321.3	2.6
A	198.4	25.0
A	181.9	45.4
A	165.1	50.8
A	150.3	8.6
A	122.5	6.8
A	77.6	1.6
A	47.2	1.5
A	42.0	1.5
A	34.7	0.3
A	19.6	3.4

TABLE S135. Harmonic vibrational frequencies (ω in cm^{-1}) along with their corresponding irreducible representations and infrared intensities (IR in km mol^{-1}) computed for the $BeF_3^-(H_2O)_3$ local minimum (3G - C_{2v}) at B3LYP/haTZ.

Irrep.	ω	IR
A_1	3706.9	121.3
A_1	3649.2	347.4
A_1	1708.8	190.2
A_1	1703.1	102.1
A_1	1129.1	477.0
A_1	722.1	180.4
A_1	594.0	0.1
A_1	339.9	18.5
A_1	188.4	0.8
A_1	168.3	15.4
A_1	73.1	0.2
A_2	3729.3	0.0
A_2	619.1	0.0
A_2	401.9	0.0
A_2	294.6	0.0
A_2	48.1	0.0
B_1	3746.3	250.4
B_1	982.1	223.1
B_1	737.4	119.0
B_1	460.0	42.8
B_1	342.7	103.8
B_1	312.0	3.0
B_1	97.4	1.0
B_1	49.2	4.5
B_2	3712.4	369.7
B_2	3674.1	18.8
B_2	1672.9	171.3
B_2	657.5	334.3
B_2	502.7	15.0
B_2	438.0	17.6
B_2	169.2	14.3
B_2	114.2	0.7
B_2	45.0	1.6

TABLE S136. Harmonic vibrational frequencies (ω in cm^{-1}) along with their corresponding irreducible representations and infrared intensities (IR in km mol^{-1}) computed for the $BeF_3^-(H_2O)_3$ local minimum (3I - C_s) at B3LYP/haTZ.

Irrep.	ω	IR
A	3769.6	224.7
A	3755.7	5.7
A	3738.1	281.6
A	3721.7	119.6
A	3705.6	60.2
A	3674.0	234.6
A	1705.0	35.1
A	1699.0	167.7
A	1673.9	264.1
A	1096.9	381.1
A	1011.3	235.2
A	662.6	291.2
A	635.5	101.6
A	628.3	150.2
A	595.8	7.0
A	525.8	25.3
A	491.4	116.7
A	443.3	32.5
A	431.3	51.0
A	403.0	8.9
A	334.7	16.4
A	328.7	77.7
A	305.3	5.8
A	301.5	1.6
A	165.3	15.6
A	158.3	1.7
A	154.3	3.7
A	107.8	0.6
A	89.6	2.4
A	71.2	0.1
A	39.0	2.4
A	36.2	1.7
A	32.4	0.0

TABLE S137. Harmonic vibrational frequencies (ω in cm^{-1}) along with their corresponding irreducible representations and infrared intensities (IR in km mol^{-1}) computed for the $BeF_3^-(H_2O)_3$ local minimum (3J - C_2) at B3LYP/haTZ.

Irrep.	ω	IR
B	3865.5	51.8
B	3672.9	589.8
B	3422.7	193.8
B	1681.0	48.5
B	1014.6	402.5
B	777.5	175.4
B	711.4	172.5
B	528.8	26.9
B	511.0	103.9
B	398.3	19.6
B	357.2	0.7
B	198.0	86.2
B	178.2	116.7
B	137.4	11.9
B	41.8	0.9
B	36.1	4.6
B	14.3	5.5
A	3865.8	28.1
A	3618.9	172.3
A	3444.1	1399.0
A	1699.2	54.4
A	1678.3	93.2
A	1109.3	464.6
A	791.5	19.9
A	654.4	2.2
A	610.6	15.2
A	407.8	51.6
A	315.8	12.4
A	230.0	7.8
A	195.9	40.4
A	156.6	2.6
A	63.9	0.1
A	24.4	0.7

TABLE S138. Harmonic vibrational frequencies (ω in cm^{-1}) along with their corresponding irreducible representations and infrared intensities (IR in km mol^{-1}) computed for the $BeF_3{}^-(H_2O)_3$ local minimum (3K - C_s) at B3LYP/haTZ.

Irrep.	ω	IR
A′	3867.7	65.9
A′	3620.2	173.1
A′	3443.5	1439.5
A′	1700.9	42.1
A′	1681.0	148.0
A′	1108.8	463.4
A′	790.1	25.3
A′	699.2	167.1
A′	611.1	16.3
A′	510.8	103.6
A′	409.6	66.2
A′	315.1	14.6
A′	230.2	9.8
A′	193.8	44.9
A′	156.1	3.2
A′	65.8	0.2
A′	38.1	0.7
A′	18.8	3.7
A″	3867.2	12.4
A″	3675.0	588.5
A″	3421.3	162.0
A″	1677.1	4.0
A″	1014.8	405.5
A″	776.4	180.9
A″	650.3	3.8
A″	529.0	26.6
A″	392.3	3.9
A″	358.0	0.1
A″	196.4	62.2
A″	161.8	124.7
A″	137.0	14.7
A″	41.0	0.0
A″	24.7	2.9

X. ωB97XD OPTIMIZED STRUCTURES

TABLE S139. Cartesian coordinates (in angstroms) for the $BeF_3{}^-$ monomer at ωB97XD/aTZ.

Be	0.0000000000	0.0000000000	0.0000000000
F	0.0000000000	0.0000000000	1.4805233177
F	1.2821708040	0.0000000000	-0.7402616588
F	-1.2821708040	0.0000000000	-0.7402616588

TABLE S140. Cartesian coordinates (in angstroms) for the $BeF_3{}^-(H_2O)_1$ global minimum (1A - C_{2v}) at ωB97XD/haTZ.

Be	0.0000000000	0.0000000000	0.7865072852
F	1.2726650784	0.0000000000	0.0154482821
F	0.0000000000	0.0000000000	2.2523522896
F	-1.2726650784	0.0000000000	0.0154482821
H	0.7350657553	0.0000000000	-1.8673398486
H	-0.7350657553	0.0000000000	-1.8673398486
O	0.0000000000	0.0000000000	-2.4956570418

TABLE S141. Cartesian coordinates (in angstroms) for the $BeF_3{}^-(H_2O)_2$ global minimum (2A - C_{2v}) at ωB97XD/haTZ.

Be	0.0000000000	0.0000000000	0.6279474732
F	0.0000000000	1.2903966175	1.3416065371
F	0.0000000000	-1.2903966175	1.3416065371
F	0.0000000000	0.0000000000	-0.8638040280
H	0.0000000000	2.6512595416	-0.0525946356
H	0.0000000000	1.9838256119	-1.3697141933
O	0.0000000000	2.8717807853	-0.9939293307
O	0.0000000000	-2.8717807853	-0.9939293307
H	0.0000000000	-2.6512595416	-0.0525946356
H	0.0000000000	-1.9838256119	-1.3697141933

TABLE S142. Cartesian coordinates (in angstroms) for the $BeF_3^-(H_2O)_2$ local minimum (2C - C_1) at ωB97XD/haTZ.

Be	-1.2091825756	0.0984404131	-0.0242625551
F	-0.2901033555	-0.7870346934	-0.7928321958
F	-2.6384144841	-0.1713099290	0.0737931411
F	-0.5910837754	1.2919672395	0.6200110418
O	2.1476587081	-1.5143601943	0.3373461464
O	1.9455844703	1.4172783171	-0.2925641876
H	1.0691380619	1.4738100970	0.1345644315
H	1.2628859438	-1.4460959887	-0.0547997256
H	2.3769697794	-0.5871058602	0.4600568170
H	1.7607702271	0.8482415988	-1.0425229139

TABLE S143. Cartesian coordinates (in angstroms) for the $BeF_3^-(H_2O)_2$ local minimum (2D - C_{2v}) at ωB97XD/haTZ.

Be	0.1822210341	0.0000000000	0.0000000000
F	1.6368327200	0.0000000000	0.0000000000
F	-0.6193901623	-1.2628554176	0.0000000000
F	-0.6193901623	1.2628554176	0.0000000000
O	-2.4078608665	0.0000000000	1.8771832930
H	-2.0237229241	0.7413780652	1.3946100290
H	-2.0237229241	-0.7413780652	1.3946100290
O	-2.4078608665	0.0000000000	-1.8771832930
H	-2.0237229241	0.7413780652	-1.3946100290
H	-2.0237229241	-0.7413780652	-1.3946100290

TABLE S144. Cartesian coordinates (in angstroms) for the $BeF_3^-(H_2O)_2$ local minimum (2E - C_s) at ωB97XD/haTZ.

Be	0.6706531463	0.7952959864	0.0000000000
F	0.0291015605	1.2107418289	1.2703407588
F	0.0291015605	1.2107418289	-1.2703407588
F	1.8232782339	-0.1224121202	0.0000000000
O	-2.0513798858	-0.2116185374	0.0000000000
H	-1.6351985134	0.2424833100	0.7458842443
H	-1.6351985134	0.2424833100	-0.7458842443
O	0.0129853521	-2.4129442353	0.0000000000
H	0.7614722450	-1.8012813224	0.0000000000
H	-0.7570311856	-1.8292670491	0.0000000000

TABLE S145. Cartesian coordinates (in angstroms) for the $BeF_3^-(H_2O)_3$ global minimum (3A - C_3) at ωB97XD/haTZ.

Be	-0.0000000131	0.0000000000	-1.3626321317
F	1.4716896460	-0.1345212067	-1.3166986061
F	-0.6193460603	1.3417812346	-1.3166986061
F	-0.8523436250	-1.2072600279	-1.3166986061
O	1.6887608061	-0.0381335161	1.4541447935
O	-0.8113558290	1.4815765284	1.4541447935
O	-0.8774050164	-1.4434430123	1.4541447935
H	1.8226869903	-0.0915822239	0.4941900643
H	-0.8320309824	1.6242843601	0.4941900643
H	-0.9906560473	-1.5327021362	0.4941900643
H	1.0455476357	0.6797739891	1.5531365713
H	-1.1114753809	0.5655838302	1.5531365713
H	0.0659277058	-1.2453578193	1.5531365713

TABLE S146. Cartesian coordinates (in angstroms) for the $BeF_3^-(H_2O)_3$ local minimum (3B - C_1) at ωB97XD/haTZ.

F	-0.3267705294	-0.1035046533	2.8647262978
Be	0.2446538058	0.1282995490	1.5488483657
F	0.7002235788	1.4634546407	1.0929688036
F	0.4107179302	-0.9953598300	0.5679549262
O	-0.1663585897	-0.3847962766	-2.1722506232
H	0.3542041798	0.4293971195	-2.0858420373
H	-0.2283844401	-0.6809156481	-1.2539406024
O	1.8271340729	1.5526762574	-1.3756538845
H	1.4350649011	1.6328430029	-0.4865384219
H	2.3433498653	0.7385589865	-1.3171532298
O	2.4877372334	-1.3525089073	-1.2349571924
H	1.9275372004	-1.3263110700	-0.4427711162
H	1.8219427914	-1.3210961706	-1.9316512856

TABLE S147. Cartesian coordinates (in angstroms) for the $BeF_3^-(H_2O)_3$ local minimum (3C - D_{3h}) at ωB97XD/haTZ.

Be	0.0000000000	0.0000000000	0.0000000000
F	0.0000000043	1.4800224029	0.0000000000
F	1.2817369969	-0.7400112052	0.0000000000
F	-1.2817370013	-0.7400111977	0.0000000000
H	-1.9659284667	1.9903650609	0.0000000000
H	-2.7066709347	0.7073614796	0.0000000000
O	-2.8708152419	1.6574659640	0.0000000000
H	2.7066709389	0.7073614637	0.0000000000
H	1.9659284784	1.9903650493	0.0000000000
H	-0.7407424722	-2.6977265246	0.0000000000
H	0.7407424563	-2.6977265290	0.0000000000
O	-0.0000000097	-3.3149319111	0.0000000000
O	2.8708152517	1.6574659471	0.0000000000

TABLE S148. Cartesian coordinates (in angstroms) for the $BeF_3^-(H_2O)_3$ local minimum (3E - C_s) at ωB97XD/haTZ.

Be	-0.0033323969	-1.3566674366	0.0000000000
F	0.6295671015	-1.6518136117	1.2950118264
F	0.6295671015	-1.6518136117	-1.2950118264
F	-1.3240394359	-0.6467574476	0.0000000000
O	-0.3241500733	0.8651221349	2.1495414361
H	0.2348812685	0.0769971198	2.1833236230
H	-1.0025252285	0.5717501521	1.5275364582
O	-0.3241500733	0.8651221349	-2.1495414361
H	0.2348812685	0.0769971198	-2.1833236230
H	-1.0025252285	0.5717501521	-1.5275364582
O	0.7622614740	2.6095739462	0.0000000000
H	0.4833485612	2.0872929239	-0.7647808140
H	0.4833485612	2.0872929239	0.7647808140

TABLE S149. Cartesian coordinates (in angstroms) for the $BeF_3^-(H_2O)_3$ local minimum (3G - C_{2v}) at ωB97XD/haTZ.

O	0.0000000000	1.7495049550	-1.0738924210
F	-1.2565919145	0.0000000000	0.7931759157
Be	0.0000000000	0.0000000000	1.6076945950
F	0.0000000000	0.0000000000	3.0573002039
F	1.2565919145	0.0000000000	0.7931759157
O	0.0000000000	-1.7495049550	-1.0738924210
H	0.7434524705	1.3245871856	-0.6266818487
H	-0.7434524705	1.3245871856	-0.6266818487
H	0.7434524705	-1.3245871856	-0.6266818487
H	-0.7434524705	-1.3245871856	-0.6266818487
O	0.0000000000	0.0000000000	-3.4165857922
H	0.0000000000	0.7492624966	-2.8047052507
H	0.0000000000	-0.7492624966	-2.8047052507

TABLE S150. Cartesian coordinates (in angstroms) for the $BeF_3^-(H_2O)_3$ local minimum (3H - C_1) at ωB97XD/haTZ.

O	-2.1727287370	-0.2512610855	-0.1621588408
F	-0.0370438806	1.1797127700	1.2150728396
Be	0.5767126857	0.7383648099	-0.0657200260
F	1.7270516412	-0.1900492342	-0.0274588836
F	-0.0587576116	1.1372922838	-1.3335581970
O	-0.1710547224	-2.4815686541	-0.2493919518
H	-1.8380316710	0.1675101354	0.6389206216
H	-1.7018875641	0.2402552054	-0.8510921223
H	0.6001989152	-1.9055954159	-0.1930030323
H	-0.9195477460	-1.8708119721	-0.2248571337
O	2.2254918672	0.3852184068	2.7630388375
H	2.4121564496	-0.0298373916	1.9134435020
H	1.3618843739	0.7741611421	2.5784063868

TABLE S151. Cartesian coordinates (in angstroms) for the $BeF_3{}^-(H_2O)_3$ local minimum (3I - C_s) at ωB97XD/haTZ.

Be	0.0883476060	1.7435822682	-1.1289243252
F	-0.6807820202	1.2519817129	0.0144597989
F	1.5433034094	2.0468166587	-1.0264672506
F	-0.5588592603	2.0708254215	-2.4302237252
O	0.2969674370	4.6199109747	-1.3921435757
H	-0.1853812646	4.0798491583	-2.0297987260
H	1.0621410481	4.0656012280	-1.1967435353
O	1.9140800169	2.0582907201	-3.8576246573
H	0.9550678744	2.0668984950	-3.7539830423
H	2.1858765904	2.0528414503	-2.9320886528
O	-1.3228924784	3.8940804068	1.0212257029
H	-1.2270049708	2.9460593929	0.8614173923
H	-0.8311060289	4.2920916835	0.2915696864

TABLE S152. Cartesian coordinates (in angstroms) for the $BeF_3{}^-(H_2O)_3$ local minimum (3K - C_s) at ωB97XD/haTZ.

Be	0.3057588812	-1.9756385756	0.0000000000
F	0.4357510785	-3.4261076032	0.0000000000
F	0.2236013207	-1.2078550853	-1.2719594422
F	0.2236013207	-1.2078550853	1.2719594422
O	-0.0995405020	1.2604134924	-2.1845583607
O	-0.0995405020	1.2604134924	2.1845583607
H	0.0891138242	0.3635448610	1.8380711521
H	-1.0546593673	1.2972081095	2.2178211032
H	0.0891138242	0.3635448610	-1.8380711521
H	-1.0546593673	1.2972081095	-2.2178211032
O	0.2112513492	3.1603125501	0.0000000000
H	0.2120852700	2.5621112868	-0.7612616857
H	0.2120852700	2.5621112868	0.7612616857

XI. ωB97XD VIBRATIONAL FREQUENCIES

TABLE S153. Harmonic vibrational frequencies (ω in cm^{-1}) along with their corresponding irreducible representations and infrared intensities (IR in km mol^{-1}) computed for the BeF_3^- monomer at ωB97XD/haTZ.

Irrep.	ω	IR
A_1'	588.4	0.0
A_2''	527.7	116.6
E'	1046.1	366.3
E'	1046.1	366.3
E'	317.1	8.5
E'	317.1	8.5

TABLE S154. Harmonic vibrational frequencies (ω in cm^{-1}) along with their corresponding irreducible representations and infrared intensities (IR in km mol^{-1}) computed for the $BeF_3^-(H_2O)_1$ global minimum (1A - C_{2v}) at ωB97XD/haTZ.

Irrep.	ω	IR
A_1	3760.8	234.0
A_1	1715.7	187.8
A_1	1087.9	439.4
A_1	587.4	1.1
A_1	334.0	24.5
A_1	184.4	10.5
A_2	433.4	0.0
B_1	710.2	183.5
B_1	511.0	73.3
B_1	36.4	1.1
B_2	1009.0	288.8
B_2	363.8	70.5
B_2	317.0	4.2
B_2	94.2	2.9

TABLE S155. Harmonic vibrational frequencies (ω in cm^{-1}) along with their corresponding irreducible representations and infrared intensities (IR in km mol^{-1}) computed for the $BeF_3^-(H_2O)_2$ global minimum (2A - C_{2v}) at ωB97XD/haTZ.

Irrep.	ω	IR
A_1	3833.1	86.6
A_1	3769.8	269.9
A_1	1718.2	77.7
A_1	1017.1	285.6
A_1	587.7	0.5
A_1	355.3	123.5
A_1	325.9	3.2
A_1	171.7	1.9
A_1	65.8	2.6
A_2	662.1	0.0
A_2	400.2	0.0
A_2	36.9	0.0
B_1	678.4	367.9
B_1	492.6	33.9
B_1	408.7	2.7
B_1	30.0	2.2
B_2	3825.8	179.0
B_2	3766.8	151.2
B_2	1703.6	290.6
B_2	1080.9	441.1
B_2	349.3	36.7
B_2	337.5	25.3
B_2	182.5	12.5
B_2	105.4	1.0

TABLE S156. Harmonic vibrational frequencies (ω in cm^{-1}) along with their corresponding irreducible representations and infrared intensities (IR in km mol^{-1}) computed for the $BeF_3{}^-(H_2O)_2$ local minimum (2C - C_1) at ωB97XD/haTZ.

Irrep.	ω	IR
A	3907.8	40.3
A	3835.4	137.6
A	3698.1	374.4
A	3581.9	494.1
A	1719.7	140.8
A	1694.8	97.1
A	1105.9	457.5
A	995.6	307.1
A	778.2	169.9
A	706.9	90.5
A	590.8	3.6
A	558.8	45.2
A	503.0	82.1
A	434.5	56.8
A	398.3	52.6
A	339.9	19.4
A	323.6	21.1
A	290.4	81.5
A	207.1	15.6
A	183.9	8.8
A	111.9	5.0
A	64.2	2.7
A	44.0	0.4
A	34.9	0.9

TABLE S157. Harmonic vibrational frequencies (ω in cm^{-1}) along with their corresponding irreducible representations and infrared intensities (IR in km mol^{-1}) computed for the $BeF_3^-(H_2O)_2$ local minimum (2D - C_{2v}) at ωB97XD/haTZ.

Irrep.	ω	IR
A_1	3799.6	127.5
A_1	1720.0	122.7
A_1	1111.6	442.5
A_1	673.4	224.5
A_1	580.9	2.5
A_1	334.2	18.3
A_1	170.1	3.8
A_1	54.4	0.5
A_2	3832.1	0.0
A_2	362.9	0.0
A_2	302.8	0.0
A_2	41.8	0.0
B_1	3791.3	158.9
B_1	1685.4	221.3
B_1	603.4	237.0
B_1	460.0	12.1
B_1	164.7	6.5
B_1	43.1	0.8
B_2	3847.3	223.0
B_2	977.2	236.1
B_2	430.5	0.3
B_2	350.7	144.8
B_2	308.7	5.1
B_2	94.5	6.1

TABLE S158. Harmonic vibrational frequencies (ω in cm^{-1}) along with their corresponding irreducible representations and infrared intensities (IR in km mol^{-1}) computed for the $BeF_3{}^{-}(H_2O)_2$ local minimum (2E - C_s) at ωB97XD/haTZ.

Irrep.	ω	IR
A′	3801.2	337.0
A′	3748.1	18.7
A′	3730.4	279.7
A′	1714.5	186.2
A′	1704.4	82.6
A′	1068.6	329.0
A′	734.4	252.1
A′	594.0	5.9
A′	536.8	131.8
A′	479.7	83.5
A′	322.4	11.4
A′	201.3	9.6
A′	172.0	7.9
A′	118.8	1.5
A′	69.3	0.3
A″	3788.5	154.6
A″	1027.0	293.8
A″	670.5	54.9
A″	530.6	66.2
A″	470.0	60.3
A″	357.7	11.9
A″	303.6	4.2
A″	109.8	0.1
A″	49.2	0.4

TABLE S159. Harmonic vibrational frequencies (ω in cm^{-1}) along with their corresponding irreducible representations and infrared intensities (IR in km mol^{-1}) computed for the $BeF_3{}^{-}(H_2O)_3$ global minimum (3A - C_3) at ωB97XD/haTZ.

Irrep.	ω	IR
E	3737.6	364.0
E	3737.6	364.1
E	3670.7	24.0
E	3670.7	23.9
E	1697.9	49.2
E	1697.9	49.2
E	1051.8	305.9
E	1051.6	306.5
E	724.8	274.7
E	724.8	274.9
E	493.2	8.1
E	493.2	8.1
E	308.7	6.3
E	308.7	6.3
E	194.5	8.0
E	194.5	8.0
E	133.3	10.2
E	133.3	10.2
A	3731.9	720.7
A	3649.2	271.6
A	1720.4	138.9
A	912.5	2.9
A	727.0	5.3
A	596.3	0.8
A	485.2	103.2
A	203.2	17.2
A	172.4	3.7
A	567.1	143.5
A	566.9	114.3
A	566.9	114.3
A	63.0	0.1
A	63.0	0.1
A	52.4	0.3

TABLE S160. Harmonic vibrational frequencies (ω in cm^{-1}) along with their corresponding irreducible representations and infrared intensities (IR in km mol^{-1}) computed for the $BeF_3^{-}(H_2O)_3$ local minimum (3B - C_1) at ωB97XD/haTZ.

Irrep.	ω	IR
A	3826.0	174.8
A	3780.5	299.3
A	3774.7	305.7
A	3696.5	237.1
A	3689.7	181.9
A	3602.7	411.4
A	1723.7	93.3
A	1705.2	105.0
A	1697.5	90.3
A	1115.8	461.2
A	988.0	339.7
A	873.8	13.3
A	790.7	176.7
A	712.3	275.3
A	668.3	102.7
A	592.2	14.4
A	541.9	19.5
A	522.6	109.0
A	504.7	85.2
A	467.9	61.6
A	439.2	19.0
A	400.2	20.9
A	339.3	14.0
A	321.1	19.1
A	213.9	16.8
A	199.0	12.6
A	181.4	9.8
A	140.9	10.9
A	117.3	3.8
A	113.8	1.3
A	70.4	0.4
A	56.1	0.4
A	32.2	1.3

TABLE S161. Harmonic vibrational frequencies (ω in cm^{-1}) along with their corresponding irreducible representations and infrared intensities (IR in km mol^{-1}) computed for the $BeF_3^-(H_2O)_3$ local minimum (3C - D_{3h}) at ωB97XD/haTZ.

Irrep.	ω	IR
A_1''	374.8	0.0
A_2''	647.1	553.4
A_2''	473.6	4.7
E''	616.9	0.0
E''	616.9	0.0
E''	385.8	0.0
E''	385.8	0.0
A	36.0	0.0
A	36.0	0.0
A	23.4	3.6
B	3848.4	177.6
B	3848.4	177.7
B	3799.0	276.6
B	3799.0	277.3
B	1695.8	278.7
B	1695.8	278.1
B	1050.0	359.7
B	1049.9	359.5
B	340.0	104.5
B	340.0	104.5
B	323.6	36.4
B	323.6	36.3
B	168.9	5.7
B	168.9	5.7
B	63.1	3.3
B	63.1	3.3

TABLE S162. Harmonic vibrational frequencies (ω in cm^{-1}) along with their corresponding irreducible representations and infrared intensities (IR in km mol^{-1}) computed for the $BeF_3^-(H_2O)_3$ local minimum (3E - C_s) at ωB97XD/haTZ.

Irrep.	ω	IR
A′	3811.4	178.5
A′	3771.1	218.9
A′	3707.0	322.9
A′	1716.0	201.6
A′	1699.9	107.0
A′	1005.2	268.7
A′	740.1	213.5
A′	687.1	64.7
A′	588.4	5.5
A′	486.4	104.5
A′	461.8	73.6
A′	356.0	42.0
A′	319.0	18.2
A′	211.9	8.7
A′	170.9	6.7
A′	113.3	1.6
A′	60.9	0.7
A′	42.5	1.4
A″	3802.5	255.8
A″	3789.7	272.6
A″	3746.6	20.5
A″	1697.0	90.7
A″	1097.2	362.5
A″	713.7	346.3
A″	660.7	21.0
A″	516.6	0.2
A″	429.9	0.3
A″	346.4	24.2
A″	332.5	11.9
A″	194.1	9.9
A″	140.5	3.8
A″	118.7	0.0
A″	49.6	0.4

TABLE S163. Harmonic vibrational frequencies (ω in cm^{-1}) along with their corresponding irreducible representations and infrared intensities (IR in km mol^{-1}) computed for the $BeF_3^-(H_2O)_3$ local minimum (3G - C_{2v}) at ωB97XD/haTZ.

Irrep.	ω	IR
A_1	3778.6	114.5
A_1	3727.6	387.5
A_1	1719.2	122.2
A_1	1714.9	179.0
A_1	1122.8	487.0
A_1	728.7	202.2
A_1	582.9	0.0
A_1	341.0	20.7
A_1	215.2	3.1
A_1	179.2	12.1
A_1	67.6	0.1
A_2	3801.0	0.0
A_2	626.9	0.0
A_2	391.3	0.0
A_2	279.8	0.0
A_2	79.7	0.0
B_1	3819.3	253.9
B_1	967.2	225.7
B_1	759.3	125.5
B_1	458.7	25.3
B_1	347.4	118.5
B_1	309.9	1.9
B_1	109.8	1.8
B_1	49.7	4.0
B_2	3788.1	387.3
B_2	3747.3	4.0
B_2	1680.2	173.8
B_2	658.4	341.8
B_2	498.3	13.3
B_2	433.6	16.7
B_2	191.9	13.1
B_2	138.0	0.8
B_2	43.8	1.8

TABLE S164. Harmonic vibrational frequencies (ω in cm^{-1}) along with their corresponding irreducible representations and infrared intensities (IR in km mol^{-1}) computed for the $BeF_3^-(H_2O)_3$ local minimum (3H - C_1) at ωB97XD/haTZ.

Irrep.	ω	IR
A	3846.1	167.3
A	3838.4	136.6
A	3824.5	148.0
A	3796.7	195.2
A	3750.5	148.2
A	3726.6	219.1
A	1714.2	126.5
A	1699.9	102.6
A	1695.4	205.8
A	1075.2	352.3
A	1025.6	267.6
A	717.9	214.1
A	659.8	185.1
A	629.8	130.2
A	589.0	4.3
A	513.0	113.0
A	475.2	95.2
A	460.4	24.1
A	441.5	67.4
A	382.9	3.4
A	343.3	31.4
A	341.7	45.6
A	328.9	15.2
A	309.6	5.9
A	193.6	8.4
A	177.1	3.9
A	161.6	6.2
A	105.6	1.7
A	101.7	0.6
A	78.5	0.5
A	63.2	1.1
A	44.6	0.4
A	29.0	1.1

TABLE S165. Harmonic vibrational frequencies (ω in cm^{-1}) along with their corresponding irreducible representations and infrared intensities (IR in km mol^{-1}) computed for the $BeF_3{}^{-}(H_2O)_3$ local minimum (3I - C_s) at ωB97XD/haTZ.

Irrep.	ω	IR
A′	3810.2	319.4
A′	3794.4	122.0
A′	3777.8	43.0
A′	3743.0	256.9
A′	1716.1	58.9
A′	1708.9	155.0
A′	1680.7	248.2
A′	1094.2	385.2
A′	682.8	288.7
A′	631.6	188.5
A′	587.1	1.2
A′	509.1	95.3
A′	437.0	53.8
A′	334.3	19.3
A′	178.5	12.0
A′	172.8	2.4
A′	168.8	4.8
A′	123.1	0.9
A′	69.0	0.1
A′	35.8	1.7
A″	3845.3	227.0
A″	3830.1	3.7
A″	997.1	241.7
A″	655.3	85.0
A″	525.2	38.9
A″	439.5	50.4
A″	407.7	20.2
A″	339.2	73.5
A″	307.4	0.7
A″	301.5	1.6
A″	108.5	2.6
A″	54.8	0.1
A″	39.3	2.0

TABLE S166. Harmonic vibrational frequencies (ω in cm^{-1}) along with their corresponding irreducible representations and infrared intensities (IR in km mol^{-1}) computed for the $BeF_3{}^-(H_2O)_3$ local minimum (3K - C_s) at ωB97XD/haTZ.

Irrep.	ω	IR
A′	3948.9	74.8
A′	3694.6	183.5
A′	3525.4	1452.3
A′	1707.6	53.0
A′	1692.2	140.3
A′	1105.3	473.8
A′	796.9	26.3
A′	727.6	151.9
A′	602.7	17.3
A′	505.2	109.1
A′	404.4	70.2
A′	313.0	13.5
A′	237.0	12.5
A′	202.6	61.2
A′	166.1	7.2
A′	62.7	0.3
A′	35.0	0.3
A′	16.9	2.8
A″	3948.2	5.9
A″	3750.1	564.7
A″	3503.5	124.6
A″	1686.9	11.1
A″	1003.4	415.4
A″	781.6	175.4
A″	665.2	13.5
A″	523.0	29.6
A″	387.4	0.8
A″	359.4	0.1
A″	201.6	42.6
A″	166.5	109.7
A″	135.3	44.8
A″	36.0	0.1
A″	10.3	3.3

XII. M06-2X OPTIMIZED STRUCTURES

TABLE S167. Cartesian coordinates (in angstroms) for the $BeF_3{}^-$ monomer at M06-2X/aTZ.

Be	0.0000000000	0.0000000000	0.0000000000
F	0.0000000000	0.0000000000	1.4678523741
F	1.2711974450	0.0000000000	-0.7339261870
F	-1.2711974450	0.0000000000	-0.7339261870

TABLE S168. Cartesian coordinates (in angstroms) for the $BeF_3{}^-(H_2O)_1$ global minimum (1A - C_{2v}) at M06-2X/haTZ.

Be	0.0000000000	0.0000000000	0.7757157136
F	1.2611787180	0.0000000000	0.0114420295
F	0.0000000000	0.0000000000	2.2302082967
F	-1.2611787180	0.0000000000	0.0114420295
H	0.7369905693	0.0000000000	-1.8537964014
H	-0.7369905693	0.0000000000	-1.8537964014
O	0.0000000000	0.0000000000	-2.4817958665

TABLE S169. Cartesian coordinates (in angstroms) for the $BeF_3{}^-(H_2O)_2$ global minimum (2A - C_{2v}) at M06-2X/haTZ.

Be	0.0000000000	0.0000000000	0.6275555372
F	0.0000000000	1.2803891573	1.3336861823
F	0.0000000000	-1.2803891573	1.3336861823
F	0.0000000000	0.0000000000	-0.8516064789
H	0.0000000000	2.6351833192	-0.0539065712
H	0.0000000000	1.9367360923	-1.3592542474
O	0.0000000000	2.8338864134	-1.0010597929
O	0.0000000000	-2.8338864134	-1.0010597929
H	0.0000000000	-2.6351833192	-0.0539065712
H	0.0000000000	-1.9367360923	-1.3592542474

TABLE S170. Cartesian coordinates (in angstroms) for the $BeF_3^-(H_2O)_2$ local minimum (2C - C_1) at M06-2X/haTZ.

Be	-0.0117892606	-0.0183762603	-0.0323665657
F	0.0429075793	-0.0176192361	1.4134944677
F	1.2232469110	-0.0663010982	-0.8517827585
F	-1.2854324294	0.0095086141	-0.7725193848
O	1.6121218823	1.8718381561	-2.8628973953
H	1.6746825155	1.3568139796	-2.0448884213
H	0.8757776321	1.4344180438	-3.3079125357
O	-0.2590680388	-0.3953046801	-3.2776460300
H	-0.8678829875	-0.2078601821	-2.5425629220
H	0.5446178753	-0.5991546144	-2.7831850003

TABLE S171. Cartesian coordinates (in angstroms) for the $BeF_3^-(H_2O)_2$ local minimum (2D - C_{2v}) at M06-2X/haTZ.

Be	0.1707387156	0.0000000000	0.0000000000
F	1.6147080817	0.0000000000	0.0000000000
F	-0.6228442935	-1.2518436379	0.0000000000
F	-0.6228442935	1.2518436379	0.0000000000
O	-2.4030599992	0.0000000000	1.8408474839
H	-2.0159945530	0.7438709267	1.3613613106
H	-2.0159945530	-0.7438709267	1.3613613106
O	-2.4030599992	0.0000000000	-1.8408474839
H	-2.0159945530	0.7438709267	-1.3613613106
H	-2.0159945530	-0.7438709267	-1.3613613106

TABLE S172. Cartesian coordinates (in angstroms) for the $BeF_3^-(H_2O)_2$ local minimum (2E - C_s) at M06-2X/haTZ.

Be	0.6600914137	0.7661715127	0.0000000000
F	0.0220189310	1.1735081944	1.2603120499
F	0.0220189310	1.1735081944	-1.2603120499
F	1.8187078558	-0.1249642627	0.0000000000
O	-2.0513869477	-0.2006799181	0.0000000000
H	-1.6352861644	0.2529610296	0.7482913488
H	-1.6352861644	0.2529610296	-0.7482913488
O	0.0213750879	-2.3863575484	0.0000000000
H	0.7757279897	-1.7798239013	0.0000000000
H	-0.7501969328	-1.8030613302	0.0000000000

TABLE S173. Cartesian coordinates (in angstroms) for the $BeF_3{}^-(H_2O)_3$ global minimum (3A - C_3) at M06-2X/haTZ.

Be	-0.0006245340	-0.0001051814	-1.3490919462
F	1.4584782643	-0.1426110999	-1.3096636432
F	-0.6067207855	1.3347798994	-1.3098023721
F	-0.8535619838	-1.1924524161	-1.3089592716
O	1.6817848666	-0.0400206968	1.4506230456
O	-0.8057668876	1.4763255333	1.4508533935
O	-0.8750415662	-1.4361637376	1.4513389243
H	1.8309223331	-0.0933628277	0.4920869840
H	-0.8344688913	1.6323654670	0.4923672536
H	-0.9961580339	-1.5389508500	0.4928695975
H	1.0415191396	0.6826589622	1.5461731263
H	-1.1113241401	0.5604369508	1.5463563576
H	0.0709620485	-1.2429000033	1.5465348910

TABLE S174. Cartesian coordinates (in angstroms) for the $BeF_3{}^-(H_2O)_3$ local minimum (3B - C_1) at M06-2X/haTZ.

F	-0.3162109393	-0.1110304645	2.8509866292
Be	0.2455711256	0.1547179663	1.5484540346
F	0.6678811635	1.4952539899	1.1188639967
F	0.4355501137	-0.9359937151	0.5564422241
O	-0.1418447525	-0.4069679204	-2.1761532333
H	0.3598528072	0.4204860814	-2.0935891553
H	-0.2145464357	-0.6907967544	-1.2529415977
O	1.8212332430	1.5311598805	-1.3511563611
H	1.4260647450	1.6318983528	-0.4652189591
H	2.3327261365	0.7129587156	-1.2853229677
O	2.4784466456	-1.3659751629	-1.2702228846
H	1.9454288163	-1.3435514898	-0.4589384456
H	1.7908993310	-1.3114224795	-1.9474632802

TABLE S175. Cartesian coordinates (in angstroms) for the $BeF_3^-(H_2O)_3$ local minimum (3C - D_{3h}) at M06-2X/haTZ.

Be	0.0000000000	0.0000000000	0.0000000000
F	0.0000000043	1.4677322241	0.0000000000
F	1.2710933899	-0.7338661158	0.0000000000
F	-1.2710933942	-0.7338661083	0.0000000000
H	-1.9351964202	1.9751366832	0.0000000000
H	-2.6781167497	0.6883609354	0.0000000000
O	-2.8409609400	1.6402295746	0.0000000000
H	2.6781167537	0.6883609196	0.0000000000
H	1.9351964318	1.9751366718	0.0000000000
H	-0.7429203335	-2.6634976028	0.0000000000
H	0.7429203178	-2.6634976072	0.0000000000
O	-0.0000000096	-3.2804591325	0.0000000000
O	2.8409609497	1.6402295579	0.0000000000

TABLE S176. Cartesian coordinates (in angstroms) for the $BeF_3^-(H_2O)_3$ local minimum (3E - C_s) at M06-2X/haTZ.

Be	0.0046787317	-1.3057355548	0.0000000000
F	0.6238880617	-1.6200686942	1.2843343259
F	0.6238880617	-1.6200686942	-1.2843343259
F	-1.2886192227	-0.5769467155	0.0000000000
O	-0.3159801301	0.8465796438	2.1894371905
H	0.2346779913	0.0499268759	2.2079593374
H	-1.0009511560	0.5757197477	1.5623109555
O	-0.3159801301	0.8465796438	-2.1894371905
H	0.2346779913	0.0499268759	-2.2079593374
H	-1.0009511560	0.5757197477	-1.5623109555
O	0.7365703129	2.5771611684	0.0000000000
H	0.4706167722	2.0530262278	-0.7688216791
H	0.4706167722	2.0530262278	0.7688216791

TABLE S177. Cartesian coordinates (in angstroms) for the $BeF_3^-(H_2O)_3$ local minimum (3G - C_{2v}) at M06-2X/haTZ.

O	0.0000000000	1.7025126012	-1.0691105851
F	-1.2459523497	0.0000000000	0.8105377397
Be	0.0000000000	0.0000000000	1.6158460810
F	0.0000000000	0.0000000000	3.0554291496
F	1.2459523497	0.0000000000	0.8105377397
O	0.0000000000	-1.7025126012	-1.0691105851
H	0.7462823335	1.2866366081	-0.6147274145
H	-0.7462823335	1.2866366081	-0.6147274145
H	0.7462823335	-1.2866366081	-0.6147274145
H	-0.7462823335	-1.2866366081	-0.6147274145
O	0.0000000000	0.0000000000	-3.4496048983
H	0.0000000000	0.7497329914	-2.8373884417
H	0.0000000000	-0.7497329914	-2.8373884417

TABLE S178. Cartesian coordinates (in angstroms) for the $BeF_3^-(H_2O)_3$ local minimum (3H - C_1) at M06-2X/haTZ.

O	-2.1704079395	-0.2299356929	-0.1478443381
F	-0.0691060711	1.0947417526	1.2196950625
Be	0.5689839737	0.6874830537	-0.0475203501
F	1.7235219738	-0.2168872483	0.0022814319
F	-0.0324539524	1.1063106930	-1.3103191221
O	-0.1690469892	-2.4552095688	-0.2684105979
H	-1.8050763613	0.1961187189	0.6393390284
H	-1.7193544263	0.2491631544	-0.8590740695
H	0.6105371065	-1.8895217336	-0.1981434808
H	-0.9169683435	-1.8431090177	-0.2275184952
O	2.2298471782	0.4230557494	2.7367431377
H	2.4103297634	-0.0005434061	1.8879191726
H	1.3436380879	0.7717245453	2.5744946206

TABLE S179. Cartesian coordinates (in angstroms) for the $BeF_3^-(H_2O)_3$ local minimum (3I - C_s) at M06-2X/haTZ.

Be	-1.3570432894	-1.0245744247	-0.0524390667
F	-2.2867170841	0.0907344440	-0.0175704958
F	-0.8311504701	-1.6403443448	1.1832219717
F	-0.8232407377	-1.5553792341	-1.3236176814
O	-0.2839671958	2.1404968550	0.0582179699
H	-1.1067494876	1.6317352127	0.0383795130
H	0.3998326643	1.4576836818	0.0372340019
O	1.3843258630	-0.3381365349	-0.0205238735
H	0.9831769196	-0.8186637106	0.7160804441
H	0.9879307235	-0.7676009712	-0.7904874665
O	0.1575155094	-3.9312461846	-0.1461772308
H	-0.0967484129	-3.3922695527	0.6141619298
H	-0.0920666022	-3.3419710360	-0.8698658157

TABLE S180. Cartesian coordinates (in angstroms) for the $BeF_3^-(H_2O)_3$ local minimum (3K - C_s) at M06-2X/haTZ.

e	0.3113352377	-1.9926848928	0.0000000000
F	0.4512751325	-3.4318745381	0.0000000000
F	0.2241856886	-1.2301022310	-1.2595290986
F	0.2241856886	-1.2301022310	1.2595290986
O	-0.1202599068	1.2531739087	-2.1522942769
O	-0.1202599068	1.2531739087	2.1522942769
H	0.0896157450	0.3535069394	1.8256343742
H	-1.0778496228	1.2900331584	2.1287207432
H	0.0896157450	0.3535069394	-1.8256343742
H	-1.0778496228	1.2900331584	-2.1287207432
O	0.2252002284	3.1990070717	0.0000000000
H	0.2373839967	2.6008702542	-0.7615898742
H	0.2373839967	2.6008702542	0.7615898742

XIII. M06-2X VIBRATIONAL FREQUENCIES

TABLE S181. Harmonic vibrational frequencies (ω in cm^{-1}) along with their corresponding irreducible representations and infrared intensities (IR in km mol^{-1}) computed for the $BeF_3{}^-$ monomer at M06-2X/haTZ.

Irrep.	ω	IR
A_1'	620.7	0.0
A_2''	540.8	125.5
E'	1107.2	358.8
E'	1107.1	358.5
E'	328.0	9.8
E'	328.0	9.9

TABLE S182. Harmonic vibrational frequencies (ω in cm^{-1}) along with their corresponding irreducible representations and infrared intensities (IR in km mol^{-1}) computed for the $BeF_3{}^-(H_2O)_1$ global minimum (1A - C_{2v}) at M06-2X/haTZ.

Irrep.	ω	IR
A_1	3770.3	237.3
A_1	1705.2	189.1
A_1	1141.5	433.4
A_1	619.7	0.6
A_1	347.7	27.3
A_1	205.3	7.1
A_2	416.6	0.0
B_1	700.3	214.6
B_1	521.9	69.3
B_1	35.4	1.2
B_2	3792.2	181.2
B_2	1073.2	284.2
B_2	352.9	56.8
B_2	330.5	23.9
B_2	131.1	0.9

TABLE S183. Harmonic vibrational frequencies (ω in cm^{-1}) along with their corresponding irreducible representations and infrared intensities (IR in km mol^{-1}) computed for the $BeF_3{}^-(H_2O)_2$ global minimum (2A - C_{2v}) at M06-2X/haTZ.

Irrep.	ω	IR
A_1	3831.9	130.2
A_1	3783.1	253.0
A_1	1706.7	82.9
A_1	1077.0	283.1
A_1	619.6	0.3
A_1	349.3	49.8
A_1	339.4	80.6
A_1	189.9	1.2
A_1	88.6	1.1
A_2	650.5	0.0
A_2	390.2	0.0
A_2	36.8	0.0
B_1	672.4	422.0
B_1	501.9	23.6
B_1	399.8	0.9
B_1	31.3	2.4
B_2	3823.7	191.9
B_2	3780.4	177.1
B_2	1691.4	294.0
B_2	1136.7	436.1
B_2	353.3	29.4
B_2	336.0	41.2
B_2	201.1	7.5
B_2	151.8	0.4

TABLE S184. Harmonic vibrational frequencies (ω in cm^{-1}) along with their corresponding irreducible representations and infrared intensities (IR in km mol^{-1}) computed for the $BeF_3{}^-(H_2O)_2$ local minimum (2C - C_1) at M06-2X/haTZ.

Irrep.	ω	IR
A	3843.6	222.5
A	3825.2	42.6
A	3760.9	247.9
A	3695.3	316.5
A	1702.1	184.3
A	1686.3	124.0
A	1160.7	447.2
A	1055.8	296.2
A	734.7	265.8
A	682.9	117.8
A	619.6	3.9
A	541.5	75.1
A	501.3	27.5
A	386.5	47.3
A	374.8	72.3
A	346.6	23.4
A	336.1	1.1
A	312.9	38.5
A	210.9	14.9
A	191.6	7.6
A	123.8	0.7
A	102.9	1.8
A	43.4	0.8
A	37.3	1.1

TABLE S185. Harmonic vibrational frequencies (ω in cm^{-1}) along with their corresponding irreducible representations and infrared intensities (IR in km mol^{-1}) computed for the $BeF_3{}^-(H_2O)_2$ local minimum (2D - C_{2v}) at M06-2X/haTZ.

Irrep.	ω	IR
A_1	3802.9	136.7
A_1	1712.2	124.5
A_1	1166.1	440.0
A_1	678.0	234.7
A_1	611.8	9.4
A_1	347.0	19.7
A_1	190.6	2.1
A_1	54.5	0.6
A_2	3832.7	0.0
A_2	358.9	0.0
A_2	306.3	0.0
A_2	100.4	0.0
B_1	3794.0	171.8
B_1	1671.6	229.2
B_1	602.3	271.6
B_1	458.2	3.9
B_1	190.2	5.1
B_1	40.3	1.0
B_2	3849.9	271.7
B_2	1040.7	230.7
B_2	438.4	0.2
B_2	360.3	137.1
B_2	320.1	22.5
B_2	132.7	1.7

TABLE S186. Harmonic vibrational frequencies (ω in cm^{-1}) along with their corresponding irreducible representations and infrared intensities (IR in km mol^{-1}) computed for the $BeF_3^-(H_2O)_2$ local minimum (2E - C_s) at M06-2X/haTZ.

Irrep.	ω	IR
A′	3808.2	364.1
A′	3751.3	23.0
A′	3736.0	287.3
A′	1698.8	232.0
A′	1687.6	46.4
A′	1124.6	322.1
A′	727.4	275.6
A′	624.2	5.1
A′	533.6	174.6
A′	480.6	57.4
A′	332.4	13.4
A′	217.0	7.8
A′	181.4	6.7
A′	132.3	2.0
A′	59.0	0.5
A″	3787.2	184.7
A″	1090.1	286.1
A″	651.7	58.3
A″	511.4	78.6
A″	465.6	71.8
A″	341.0	6.0
A″	311.7	3.8
A″	130.8	0.6
A″	42.7	0.4

TABLE S187. Harmonic vibrational frequencies (ω in cm^{-1}) along with their corresponding irreducible representations and infrared intensities (IR in km mol^{-1}) computed for the $BeF_3^-(H_2O)_3$ global minimum (3A - C_3) at M06-2X/haTZ.

Irrep.	ω	IR
A	3748.4	368.5
A	3748.3	368.9
A	3746.3	771.6
A	3688.9	33.7
A	3688.7	33.3
A	3662.7	215.0
A	1700.7	152.6
A	1676.0	54.2
A	1675.8	54.2
A	1108.8	298.7
A	1108.6	298.4
A	904.6	3.3
A	704.6	10.1
A	699.1	305.1
A	698.4	313.1
A	623.9	3.8
A	553.2	112.4
A	551.3	133.2
A	550.2	160.3
A	487.5	80.4
A	465.8	11.2
A	465.3	11.4
A	317.5	7.1
A	317.4	7.1
A	215.5	11.5
A	207.4	5.6
A	207.1	5.7
A	191.0	6.7
A	154.3	10.2
A	154.0	10.3
A	43.6	0.3
A	41.5	0.0
A	41.4	0.1

TABLE S188. Harmonic vibrational frequencies (ω in cm^{-1}) along with their corresponding irreducible representations and infrared intensities (IR in km mol^{-1}) computed for the $BeF_3^-(H_2O)_3$ local minimum (3B - C_1) at M06-2X/haTZ.

Irrep.	ω	IR
A	3814.4	262.2
A	3789.3	364.0
A	3777.0	270.8
A	3721.5	151.1
A	3699.1	189.4
A	3622.2	398.7
A	1706.1	102.7
A	1684.3	149.9
A	1675.1	59.7
A	1175.1	449.7
A	1054.0	333.3
A	867.3	17.4
A	749.4	215.6
A	682.6	335.5
A	655.2	85.4
A	624.2	19.4
A	521.6	12.0
A	514.9	101.0
A	503.8	116.9
A	449.5	49.4
A	407.3	24.4
A	372.2	13.7
A	348.2	12.8
A	330.6	22.0
A	221.3	15.3
A	200.2	13.9
A	181.9	9.0
A	156.1	11.7
A	133.2	0.5
A	131.1	2.5
A	58.7	0.2
A	50.1	0.8
A	32.5	1.1

TABLE S189. Harmonic vibrational frequencies (ω in cm^{-1}) along with their corresponding irreducible representations and infrared intensities (IR in km mol^{-1}) computed for the $BeF_3^-(H_2O)_3$ local minimum (3C - D_{3h}) at M06-2X/haTZ.

Irrep.	ω	IR
A_1''	379.0	0.0
A_2''	654.4	610.2
A_2''	481.6	1.7
E''	617.9	0.0
E''	617.9	0.0
E''	391.2	0.0
E''	391.2	0.0
A	39.0	0.0
A	38.9	0.0
A	29.0	3.8
B	3850.7	212.9
B	3850.7	213.1
B	3840.0	0.0
B	3808.9	0.0
B	3803.7	302.0
B	3803.7	301.8
B	1706.9	0.0
B	1685.3	283.9
B	1685.3	284.0
B	1107.4	357.1
B	1107.0	357.4
B	619.7	0.0
B	353.4	21.6
B	353.2	21.6
B	333.5	126.8
B	333.5	126.7
B	316.6	0.0
B	190.7	3.3
B	190.6	3.2
B	173.6	0.0
B	165.7	0.0
B	81.9	1.2
B	81.9	1.2

TABLE S190. Harmonic vibrational frequencies (ω in cm^{-1}) along with their corresponding irreducible representations and infrared intensities (IR in km mol^{-1}) computed for the $BeF_3{}^-(H_2O)_3$ local minimum (3E - C_s) at M06-2X/haTZ.

Irrep.	ω	IR
A′	3814.8	199.3
A′	3773.6	229.8
A′	3724.8	336.9
A′	1700.7	242.2
A′	1673.7	69.2
A′	1068.3	266.5
A′	729.2	236.2
A′	665.7	77.3
A′	618.4	6.3
A′	491.9	97.5
A′	442.6	94.9
A′	335.4	6.5
A′	328.9	48.6
A′	223.7	7.1
A′	174.5	4.6
A′	128.2	1.6
A′	51.7	1.0
A′	31.5	1.2
A″	3811.0	538.9
A″	3798.9	32.6
A″	3754.4	2.4
A″	1682.1	95.3
A″	1148.2	358.5
A″	700.4	380.7
A″	642.4	21.2
A″	492.4	1.7
A″	413.7	0.0
A″	345.2	31.2
A″	324.7	10.4
A″	207.6	6.6
A″	154.7	3.3
A″	137.3	2.0
A″	43.8	0.5

TABLE S191. Harmonic vibrational frequencies (ω in cm^{-1}) along with their corresponding irreducible representations and infrared intensities (IR in km mol^{-1}) computed for the $BeF_3^-(H_2O)_3$ local minimum (3G - C_{2v}) at M06-2X/haTZ.

Irrep.	ω	IR
A_1	3785.3	103.6
A_1	3747.0	402.7
A_1	1710.7	174.6
A_1	1697.0	144.2
A_1	1176.4	487.4
A_1	733.0	212.1
A_1	615.6	0.5
A_1	352.9	24.1
A_1	222.2	0.3
A_1	196.7	13.9
A_1	79.3	0.2
A_2	3804.5	0.0
A_2	594.7	0.0
A_2	376.6	0.0
A_2	301.8	0.0
A_2	107.8	0.0
B_1	3825.2	300.7
B_1	1031.0	223.0
B_1	746.0	135.0
B_1	476.0	68.2
B_1	368.3	69.9
B_1	322.0	21.9
B_1	142.9	0.0
B_1	47.3	3.7
B_2	3800.1	386.5
B_2	3756.8	1.6
B_2	1663.6	172.6
B_2	636.6	388.6
B_2	491.2	3.2
B_2	404.1	17.9
B_2	202.7	11.3
B_2	141.4	0.2
B_2	40.2	2.1

TABLE S192. Harmonic vibrational frequencies (ω in cm^{-1}) along with their corresponding irreducible representations and infrared intensities (IR in km mol^{-1}) computed for the $BeF_3^-(H_2O)_3$ local minimum (3H - C_1) at M06-2X/haTZ.

Irrep.	ω	IR
A	3847.2	242.2
A	3834.2	208.8
A	3818.6	93.7
A	3803.7	229.2
A	3766.4	107.4
A	3743.4	229.7
A	1700.3	119.4
A	1683.0	304.9
A	1680.8	31.3
A	1128.9	351.4
A	1079.2	256.6
A	705.5	269.7
A	651.1	186.2
A	630.2	63.0
A	610.7	73.7
A	513.9	126.4
A	466.1	103.0
A	456.6	5.1
A	449.7	89.6
A	376.3	3.0
A	339.1	19.5
A	329.4	8.3
A	322.9	36.3
A	316.4	32.2
A	211.5	6.2
A	188.9	3.6
A	174.9	4.9
A	138.0	1.5
A	127.5	0.8
A	102.8	0.7
A	55.4	1.1
A	41.5	0.3
A	28.9	1.3

TABLE S193. Harmonic vibrational frequencies (ω in cm^{-1}) along with their corresponding irreducible representations and infrared intensities (IR in km mol^{-1}) computed for the $BeF_3{}^-(H_2O)_3$ local minimum (3I - C_s) at M06-2X/haTZ.

Irrep.	ω	IR
A	3846.5	281.3
A	3829.9	0.2
A	3818.6	328.6
A	3798.8	129.6
A	3779.1	39.4
A	3756.6	285.0
A	1704.9	76.8
A	1693.4	149.0
A	1665.4	256.5
A	1147.4	379.6
A	1060.9	234.8
A	681.8	284.2
A	645.1	110.5
A	629.3	223.1
A	616.8	4.6
A	517.9	37.3
A	488.8	118.6
A	452.9	58.1
A	432.3	53.4
A	417.8	16.7
A	345.5	19.9
A	324.4	66.4
A	315.8	0.7
A	293.8	5.7
A	195.5	9.3
A	193.8	1.9
A	186.7	2.8
A	141.8	0.8
A	135.7	0.3
A	104.6	0.9
A	57.9	0.2
A	38.2	1.8
A	35.7	1.7

TABLE S194. Harmonic vibrational frequencies (ω in cm^{-1}) along with their corresponding irreducible representations and infrared intensities (IR in km mol^{-1}) computed for the $BeF_3{}^-(H_2O)_3$ local minimum (3K - C_s) at M06-2X/haTZ.

Irrep.	ω	IR
A′	3932.7	98.4
A′	3721.8	184.8
A′	3547.1	1427.1
A′	1689.2	85.5
A′	1673.2	139.5
A′	1159.9	467.3
A′	763.8	34.6
A′	711.4	165.3
A′	632.8	14.8
A′	519.1	113.2
A′	375.0	73.5
A′	324.1	13.8
A′	233.3	13.0
A′	181.2	60.3
A′	156.1	19.9
A′	57.0	0.5
A′	34.5	0.4
A′	17.2	2.0
A″	3931.8	3.9
A″	3775.4	524.3
A″	3525.6	111.1
A″	1666.5	15.2
A″	1066.6	395.6
A″	740.4	215.2
A″	634.5	16.7
A″	485.7	34.1
A″	364.1	0.5
A″	355.2	0.0
A″	203.0	38.7
A″	147.9	9.5
A″	98.9	141.1
A″	32.2	0.6
A″	-5.0	7.4
